# Cardiovascular outcomes in adults with hypertension with evening versus morning dosing of usual antihypertensives in the UK (TIME study): a prospective, randomised, open-label, blinded-endpoint clinical trial

**DOI:** 10.1016/S0140-6736(22)01786-X

**Published:** 2022-10-22

**Authors:** Isla S Mackenzie, Amy Rogers, Neil R Poulter, Bryan Williams, Morris J Brown, David J Webb, Ian Ford, David A Rorie, Greg Guthrie, J W Kerr Grieve, Filippo Pigazzani, Peter M Rothwell, Robin Young, Alex McConnachie, Allan D Struthers, Chim C Lang, Thomas M MacDonald, TIME Study Group

**Affiliations:** a MEMO Research, Ninewells Hospital and Medical School, University of Dundee, Dundee, UK; b Division of Molecular and Clinical Medicine, Ninewells Hospital and Medical School, University of Dundee, Dundee, UK; c School of Public Health, Imperial College London, London, UK; d NIHR University College London Hospitals Biomedical Research Centre and University College London, London, UK; e Queen Mary University of London, London, UK; f British Heart Foundation/University Centre for Cardiovascular Science, University of Edinburgh, Edinburgh, UK; g The Robertson Centre for Biostatistics, University of Glasgow, Glasgow, UK; h Department of Neurology, Aberdeen Royal Infirmary, Aberdeen, UK; i Wolfson Centre for Prevention of Stroke and Dementia, Nuffield Department of Clinical Neurosciences, University of Oxford, Oxford, UK

## Abstract

**Background:**

Studies have suggested that evening dosing with antihypertensive therapy might have better outcomes than morning dosing. The Treatment in Morning versus Evening (TIME) study aimed to investigate whether evening dosing of usual antihypertensive medication improves major cardiovascular outcomes compared with morning dosing in patients with hypertension.

**Methods:**

The TIME study is a prospective, pragmatic, decentralised, parallel-group study in the UK, that recruited adults (aged ≥18 years) with hypertension and taking at least one antihypertensive medication. Eligible participants were randomly assigned (1:1), without restriction, stratification, or minimisation, to take all of their usual antihypertensive medications in either the morning (0600–1000 h) or in the evening (2000–0000 h). Participants were followed up for the composite primary endpoint of vascular death or hospitalisation for non-fatal myocardial infarction or non-fatal stroke. Endpoints were identified by participant report or record linkage to National Health Service datasets and were adjudicated by a committee masked to treatment allocation. The primary endpoint was assessed as the time to first occurrence of an event in the intention-to-treat population (ie, all participants randomly assigned to a treatment group). Safety was assessed in all participants who submitted at least one follow-up questionnaire. The study is registered with EudraCT (2011-001968-21) and ISRCTN (18157641), and is now complete.

**Findings:**

Between Dec 17, 2011, and June 5, 2018, 24 610 individuals were screened and 21 104 were randomly assigned to evening (n=10 503) or morning (n=10 601) dosing groups. Mean age at study entry was 65·1 years (SD 9·3); 12 136 (57·5%) participants were men; 8968 (42·5%) were women; 19 101 (90·5%) were White; 98 (0·5%) were Black, African, Caribbean, or Black British (ethnicity was not reported by 1637 [7·8%] participants); and 2725 (13·0%) had a previous cardiovascular disease. By the end of study follow-up (March 31, 2021), median follow-up was 5·2 years (IQR 4·9–5·7), and 529 (5·0%) of 10 503 participants assigned to evening treatment and 318 (3·0%) of 10 601 assigned to morning treatment had withdrawn from all follow-up. A primary endpoint event occurred in 362 (3·4%) participants assigned to evening treatment (0·69 events [95% CI 0·62–0·76] per 100 patient-years) and 390 (3·7%) assigned to morning treatment (0·72 events [95% CI 0·65–0·79] per 100 patient-years; unadjusted hazard ratio 0·95 [95% CI 0·83–1·10]; p=0·53). No safety concerns were identified.

**Interpretation:**

Evening dosing of usual antihypertensive medication was not different from morning dosing in terms of major cardiovascular outcomes. Patients can be advised that they can take their regular antihypertensive medications at a convenient time that minimises any undesirable effects.

**Funding:**

British Heart Foundation.

## Introduction

Hypertension, or high blood pressure, is a key risk factor for cardiovascular disease worldwide.^1^ Adequately controlling blood pressure reduces the risk of major cardiovascular events, including stroke, ischaemic heart disease, and cardiovascular death.^2^ Clinical trials supporting the cardiovascular benefits of antihypertensive therapy primarily use conventional morning dosing. When measured using 24 h ambulatory monitoring, normal blood pressure exhibits a diurnal rhythm, with lower pressures during night-time sleep (referred to as dipping), followed by a morning increase or surge in blood pressure. The risk of adverse cardiovascular outcomes is increased in people whose blood pressure does not have the typical diurnal variation, such as reduced, reversed, or extreme dipping patterns, and high night-to-day blood pressure ratios.^3^, ^4^ Additionally, cardiovascular events are temporally associated with the morning blood pressure surge.^5^ Evening dosing of antihypertensive medication has been suggested to potentially be more effective at normalising the diurnal rhythm, lowering 24 h blood pressure, and preventing the long-term cardiovascular sequelae of hypertension than morning dosing.

**Research in context**

**Evidence before this study**

A 2022 systematic review for the International Society of Hypertension identified eight studies testing the effect of bedtime dosing of antihypertensive drugs on outcomes. All eight studies were determined to have a high risk of bias and only two were completed randomised studies that compared morning and bedtime dosing of antihypertensive medication for cardiovascular outcomes. The MAPEC study and the Hygia Chronotherapy studies were both prospective, randomised, open-label, blinded-endpoint design studies done by a single research group in Spain. The 2010 MAPEC study (2156 participants) reported a substantial reduction in major cardiovascular events (cardiovascular deaths, myocardial infarction, ischaemic stroke, and haemorrhagic stroke) in the bedtime treatment group compared with the morning treatment group (adjusted relative risk 0·33 [95% CI 0·19–0·55]). The subsequent and larger Hygia Chronotherapy study (19 084 participants) also reported a substantial reduction in cardiovascular events (cardiovascular death, myocardial infarction, coronary revascularisation, heart failure, and stroke) in the bedtime treatment group compared with the morning treatment group (adjusted hazard ratio 0·55 [95% CI 0·50–0·61]). Several expert commentators have questioned the methods and plausibility of the effect sizes reported in both of these studies. There was a clear need for an independent, large, randomised trial testing the hypothesis that bedtime, or evening, dosing of antihypertensives would be better than morning dosing in terms of major cardiovascular outcomes.

**Added value of this study**

The Treatment in Morning versus Evening (TIME) study was a large, pragmatic, decentralised, prospective, randomised, open-label, blinded-endpoint, superiority trial conducted in the UK, comparing cardiovascular outcomes in adults with hypertension randomly assigned to evening versus morning dosing of their usual antihypertensive medications. We found no difference between the evening and morning dosing groups for the primary composite outcome of vascular death or hospitalisation for non-fatal myocardial infarction or non-fatal stroke over a median follow-up time of 5·2 years (IQR 4·9–5·7). Additionally, we found no difference in all-cause mortality between the evening and morning dosing groups.

**Implications of all the available evidence**

These findings are an important addition to the hitherto limited and controversial randomised clinical trial evidence available comparing the effects of dosing times of antihypertensive medication with regard to cardiovascular outcomes. Given the continued controversy around MAPEC and the Hygia Chronotherapy trial, the evidence from the TIME trial suggests that dosing time should not be a significant consideration when advising most patients on managing their blood pressure. Instead, clinicians should focus on selecting appropriate medications and supporting adherence to agreed treatment plans.

A 2005 study comparing the effect of dosing of a single antihypertensive on awakening and at bedtime reported restoration of night-time dipping status with bedtime dosing.^6^ However, the HARMONY trial reported no difference of morning versus evening dosing time, on either 24 h ambulatory blood pressure or clinic-measured blood pressure.^7^ A recent systematic review^8^ identified only two completed randomised studies that have compared cardiovascular outcomes with morning and bedtime dosing of antihypertensive medication in adults with hypertension: the MAPEC study^9^ and the subsequent larger study from the same research group, the Hygia Chronotherapy Trial.^10^ Both studies reported a reduction in all major cardiovascular events with bedtime treatment compared with morning treatment. The effect size in each of these studies was considered by many to be implausibly large.^8^, ^11^ Additionally, there is a paucity of evidence on the potential harms of bedtime dosing related to excessive night-time blood pressure lowering (eg, potential increased risk of falls, glaucoma, and cerebrovascular events).^12^, ^13^, ^14^ Dosing time might also affect medication adherence. Although previous research has found that evening dosing is generally associated with worse medication adherence,^15^, ^16^ the convenience of evening dosing might enhance adherence in some patients.

The relative pros and cons of evening dosing have been debated, and controversy persists, with some researchers suggesting that the Hygia Chronotherapy Trial might not have been a true randomised controlled trial.^8^, ^17^, ^18^ In the Treatment in Morning versus Evening (TIME) study, we aimed to investigate whether evening dosing of antihypertensive medication improves major cardiovascular outcomes compared with morning dosing in patients with hypertension treated with their usual antihypertensive medications.

## Methods

### Study design and participants

In this prospective, randomised, open-label, blinded-endpoint, controlled, parallel-group superiority trial (TIME), patients with treated hypertension were recruited via various methods, predominantly by screening UK National Health Service (NHS) primary care practice lists and writing to potentially suitable participants to invite them to register on the TIME study portal.^19^, ^20^ Patients were eligible if they were UK residents, aged at least 18 years, with diagnosed hypertension, and taking at least one antihypertensive medication daily. Participants were required to have an email address and be registered with a UK general practitioner. People undertaking regular overnight shift work or taking antihypertensive medications at more than one dosing time daily were excluded. Recruitment took place in a rolling pilot, which then continued into the main trial. Participants who had registered on the study website after the time that registration closed were able to complete their enrolment at a later date. All participants provided written informed consent.

The protocol has been published^21^ and is in the appendix (pp 19–46). The study was approved by the East of Scotland Research Ethics Committee (11/AL/0309). The University of Dundee (Dundee, zUK) was the study sponsor and study data were managed by the University of Dundee and analysed by statisticians based at the Robertson Centre for Biostatistics, University of Glasgow (Glasgow, UK).

### Randomisation and masking

Enrolled and consenting participants were randomly assigned (1:1), with no restriction, stratification, or minimisation, using a computer algorithm, to take their usual prescribed antihypertensive therapy either in the morning (0600–1000 h) or the evening (2000–0000 h) and were advised of their dosing time allocation via email. The randomisation algorithm used randomly generated bits (0s and 1s, where 0 = morning and 1 = evening), which were allocated to participants as they completed enrolment. Patients and investigators were not masked to group allocation due to the nature of the intervention, but endpoint assessors were masked to group allocation.

### Procedures

The study was designed as a decentralised trial, which typically has no requirement for in-person study visits and all study activities are undertaken in or near the participant's home.^22^ All screening, consent, randomisation, and follow-up were done through an online study portal and by email.

Participants randomly assigned to evening dosing and taking diuretics as one of their medications were instructed to attempt evening dosing of diuretic along with other medication, with instructions to move their dosing time of only the diuretic to early evening (1800 h), then morning, if troubled by persistent nocturia. All participants were asked to remain on their randomised dosing time for the duration of the study.

Participants were invited to complete online follow-up questionnaires at regular intervals (1 month after randomisation and every 3 months thereafter). The follow-up questionnaires asked if the participant was currently taking their blood pressure lowering medication at their assigned time and if they had experienced any events of interest since their last follow-up submission (ie, potential endpoint events and prespecified side-effects). Any participants who indicated that they were not currently taking their medication at their study-assigned time were asked to indicate whether this was due to experiencing side-effects, medical advice, or inconvenience, and they were continued in the study. Participants who reported non-adherence to assigned dosing time were free to return to their assigned dosing time at a later date.

We also requested record-linked NHS hospitalisation and death data from NHS Digital, the Secure Anonymised Information Linkage (known as SAIL) databank, Public Health Scotland, and Health and Social Care (HSC) Northern Ireland at annual intervals and after the end of the study.^23^ Participant-nominated alternative contacts were approached if participants did not respond as expected to consecutive follow-up invitations. Potential endpoint events were identified, and packages of de-identified clinical information were created by interrogating medical records.

Participants were asked at baseline if they owned a home blood pressure monitor and this subset of participants were asked whether they would be willing to provide home blood pressure measurements.^24^, ^25^, ^26^ Those willing to do this were invited to provide sets of home blood pressure measurements in the morning and evening at 3-monthly intervals via an online portal throughout the study.

Safety was assessed by participant reporting via the online follow-up questionnaires (hospitalised and non-hospitalised falls and fractures, and other prespecified symptoms [ie, dizziness or light-headedness, upset stomach or indigestion, diarrhoea, muscle aches, excessive visits to the toilet during the day or night, sleep problems, feeling generally less well, and other]) and linked hospitalisation data (glaucoma events).

Additionally, several substudies were done addressing sleep quality,^27^ cognitive function, mood, and chronotype, which will be reported elsewhere.

### Outcomes

The primary outcome was the composite cardiovascular endpoint of vascular death or hospitalisation for non-fatal myocardial infarction or non-fatal stroke, analysed as the time to first event. Secondary outcomes were hospitalisation for non-fatal myocardial infarction, hospitalisation for non-fatal stroke, vascular death, all-cause mortality, hospitalisation or death from congestive heart failure, participant-reported adherence to randomised dosing schedule (with particular reference to patients also taking diuretic therapy), and prespecified participant-reported adverse events (falls, fractures, and other symptoms). Hospitalisations for glaucoma were added as a secondary outcome during the trial because of concerns expressed by the ophthalmological community that nocturnal hypotension might worsen glaucoma outcomes. All cardiovascular outcomes are defined in the Endpoint Committee Charter (appendix pp 47–97).

An independent clinical endpoint committee, based at the University of Dundee and comprising specialist cardiology and stroke physicians who were masked to dosing time allocation, adjudicated all components of the primary composite outcome and selected secondary outcomes (all-cause mortality, and hospitalisations or death due to heart failure).

### Statistical analysis

We calculated that at least 631 participants would be required to have an adjudicated first primary endpoint event to detect a 20% superiority of evening versus morning dosing between the randomised groups with 80% power. The trial was initially planned to randomly assign 10 269 participants and follow them up for 5 years. However, because of lower-than-expected cardiovascular event rates in other trials with similar participant characteristics,^28^, ^29^ the sample size was increased to at least 20 000 to ensure the study would be powered to report reliable results. The primary endpoint, and secondary cardiovascular and mortality endpoints, were assessed in the intention-to-treat population, which comprised all participants randomly assigned to treatment. Safety was assessed in all participants who submitted at least one follow-up questionnaire, except for glaucoma, which was assessed in the intention-to-treat population.

We assessed the primary endpoint as the time to the occurrence of the first primary endpoint event on an intention-to-treat basis using an unadjusted Cox proportional-hazards model. We also assessed the secondary cardiovascular and mortality outcomes using this approach. We present time-to-event curves for the primary composite outcome as a cumulative incidence function, censoring for the competing risk of deaths not included in the endpoint, and we present a Kaplan-Meier curve for all-cause mortality. We calculated event rates for the cardiovascular and mortality endpoints using Poisson tests. To compare differences between groups for the remaining secondary outcomes, we used Yates' χ^2^ test for categorical outcomes and the independent samples *t* test for continuous outcomes; p values of less than 0·05 were considered to be significant. We calculated between-group differences with 95% confidence intervals for prespecified symptoms using Yates' χ^2^ test.

We did prespecified subgroup analyses of the primary outcome by age (≤median *vs* >median age), sex (female *vs* male), BMI (≤median *vs* >median BMI), smoking (never *vs* former *vs* current), previous heart attack (yes *vs* no), previous stroke (yes *vs* no), previous cardiovascular disease (yes *vs* no), diabetes (yes *vs* no), taking angiotensin-converting enzyme inhibitor (ACE; yes *vs* no), taking angiotensin II receptor blocker (ARB; yes *vs* no), taking ACE or ARB (yes *vs* no), taking beta blocker (yes *vs* no), taking calcium channel blocker (yes *vs* no), number of antihypertensives (≤three *vs* >three), taking alpha blocker (yes *vs* no), and taking diuretics (yes *vs* no).

We did all analyses using R (version 4.1.1). The statistical analysis plan is available in the appendix (pp 111–21). A steering committee oversaw the trial. Additionally, an independent data monitoring committee (charter in the appendix [pp 98–110]) met regularly to monitor the safety of participants and review the unblinded trial data, including a prespecified early stopping guideline of a p value of less than 0·001 for the primary composite outcome to recommend stopping for overwhelming benefit of either dosing intervention. However, this committee did not recommend early stopping, and the study reached its planned conclusion.

The study is registered with EudraCT (2011-001968-21) and ISRCTN (18157641).

### Role of the funding source

The funder and sponsor had no role in the study design, data collection, data analysis, data interpretation, writing of the report, or decision to submit for publication.

## Results

**Figure 1:**
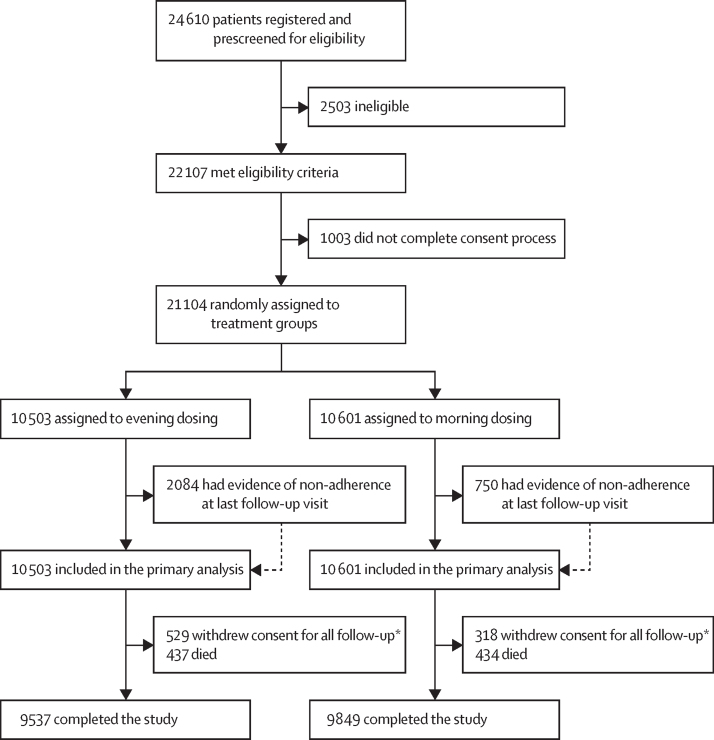
Study profile *Reasons for withdrawal of consent to follow-up are listed in the appendix (p 4); participants who withdrew consent for all follow-up were included in the time-to-event analysis up to the point of withdrawal.

Between Dec 17, 2011, and June 5, 2018 (with the main trial recruitment period running between June 10, 2014, and March 31, 2017), 24 610 patients were screened and 21 104 were enrolled and randomly assigned to evening (n=10 503) or morning (n=10 601) dosing groups (figure 1). Baseline characteristics were balanced between the two study groups (table 1; appendix p 3). The mean age of participants at study entry was 65·1 years (SD 9·3); 8968 (42·5%) participants were women; 12 136 (57·5%) were men; 19 101 (90·5%) were White; 98 (0·5%) were Black, African, Caribbean, or Black British (ethnicity was not reported by 1637 [7·8%] participants); and 2725 (12·9%) had previous cardiovascular disease.

**Table 1:** Baseline demographic and clinical characteristics

		**Evening dosing group (n=10 503)**	**Morning dosing group (n=10 601)**
Age, years		65·0 (9·3)	65·2 (9·2)
Sex			
	Male	6041 (57·5%)	6095 (57·5%)
	Female	4462 (42·5%)	4506 (42·5%)
Place of residence			
	England	9243 (88·0%)	9289 (87·6%)
	Scotland	873 (8·3%)	943 (8·9%)
	Wales	384 (3·7%)	366 (3·5%)
	Northern Ireland	3 (<0·1%)	3 (<0·1%)
Ethnicity			
	White	9476 (90·2%)	9625 (90·8%)
	Black, African, Caribbean, or Black British	45 (0·4%)	53 (0·5%)
	Asian or Asian British	74 (0·7%)	81 (0·8%)
	Mixed or multiple	34 (0·3%)	52 (0·5%)
	Other	12 (0·1%)	15 (0·1%)
	Not reported	862 (8·2%)	775 (7·3%)
Smoking history			
	Never	6066 (57·8%)	6012 (56·7%)
	Former	3944 (37·6%)	4063 (38·3%)
	Current	428 (4·1%)	457 (4·3%)
	Missing	65 (0·6%)	69 (0·7%)
Systolic blood pressure, mm Hg*			
	n	5052	5026
	Mean	135·0 (13·3)	134·8 (13·3)
Diastolic blood pressure, mm Hg*			
	n	5044	5023
	Mean	79·1 (9·2)	78·8 (9·3)
BMI, kg/m^2^†			
	n	9713	9791
	Mean	28·4 (4·8)	28·4 (4·9)
Cardiovascular history‡			
	Evidence of cardiovascular disease§	1364 (13·0%)	1361 (12·8%)
	Previous myocardial infarction	516 (4·9%)	469 (4·4%)
	Angina, requiring medical treatment	302 (2·9%)	334 (3·2%)
	Previous stroke	260 (2·5%)	237 (2·2%)
	Previous transient ischaemic attack	429 (4·1%)	448 (4·2%)
	Peripheral vascular disease	164 (1·6%)	160 (1·5%)
Other medical history‡			
	Diabetes, any	1354 (12·9%)	1413 (13·3%)
	Diabetes, requiring medical treatment	995 (9·5%)	1074 (10·1%)
	Asthma	1050 (10·0%)	1034 (9·8%)
	Arthritis, requiring medical treatment	685 (6·5%)	735 (6·9%)
	Impaired kidney function	327 (3·1%)	355 (3·3%)
	Chronic obstructive pulmonary disease	316 (3·0%)	300 (2·8%)
	Antihypertensive use at study entry, number of medications‡¶	1·49 (0·68)	1·50 (0·71)

Data are mean (SD), n, or n (%).

* Self-reported last known measurement.

† Derived from self-reported height and bodyweight.

‡ Self-reported medical history.

§ Defined as self-reported history of any angina, myocardial infarction, stroke, transient ischaemic attack, or peripheral vascular disease.

¶ Baseline antihypertensive medications are summarised in the appendix (p 3).

Study follow-up ended on March 31, 2021 (data cutoff), by which time we estimated that at least 631 participants had experienced a first primary outcome event. 437 (4·2%) of 10 503 participants assigned to the evening dosing group and 434 (4·1%) of 10 601 assigned to the morning dosing group had died before the end of the study. Median follow-up was 5·2 years (IQR 4·9–5·7) and the maximum follow-up was 9·3 years. Of 11 314 (53·6%) participants who retrospectively reported their pre-study dosing time, 9961 (85·4%) had previously taken all their antihypertensive medications in the morning.

All 21 104 randomised participants were included in the analysis set. 2453 (11·6%) participants withdrew from active questionnaire-based follow-up, of whom 1539 (62·7%) were in the evening dosing group and 914 (37·3%) were in the morning dosing group and so they were not included in the participant-reported safety analysis after the point of withdrawal. Consent to access record-linked data and medical records was withdrawn by 529 (5·0%) participants in the evening dosing group and 318 (3·0%) in the morning dosing group; a list of reasons for withdrawal is in the appendix (p 4). For those participants who withdrew consent, only events that occurred before withdrawal of consent could be included in the analysis.

**Figure 2:**
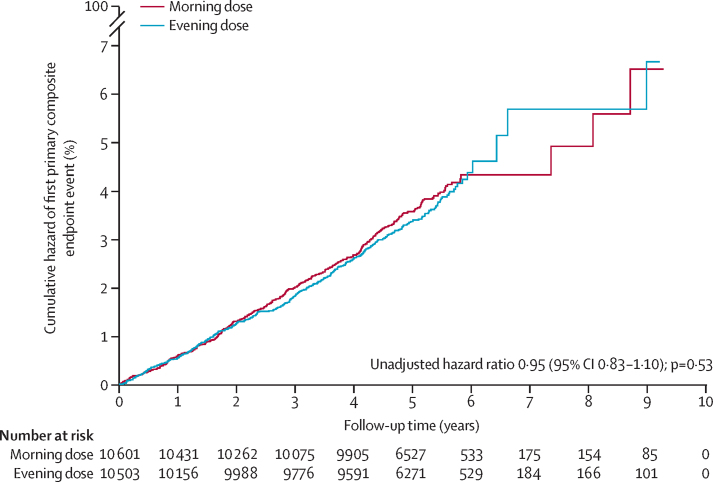
Cumulative hazard of the first primary composite endpoint event, accounting for the competing risk of deaths not included in the endpoint (intention-to-treat population; n=21 104) The primary composite endpoint was vascular death or hospitalisation for non-fatal myocardial infarction or non-fatal stroke.

Primary endpoint events occurred in 362 (3·4%) participants assigned to evening dosing (0·69 events [95% CI 0·62–0·76] per 100 patient-years) and 390 (3·7%) assigned to morning dosing (0·72 events [95% CI 0·65–0·79] per 100-patient years; unadjusted hazard ratio 0·95 [95% CI 0·83–1·10]; p=0·53; figure 2, table 2). This finding did not vary for prespecified subgroup analyses (appendix p 6). Similarly, secondary cardiovascular and mortality endpoints were not different between timing groups (table 2; appendix pp 7–11).

**Table 2:** Primary composite outcome and secondary cardiovascular and mortality outcomes (intention-to-treat population; n=21 104)

		**Evening dosing group (n=10 503)**		**Morning dosing group (n=10 601)**		**Hazard ratio (95% CI)**	**p value**
		Participants, n (%)	Rate per 100 patient-years (95% CI)	Participants, n (%)	Rate per 100 patient-years (95% CI)		
Primary composite endpoint		362 (3·4%)	0·69 (0·62–0·76)	390 (3·7%)	0·72 (0·65–0·79)	0·95 (0·83–1·10)	0·53
Secondary cardiovascular and mortality endpoints							
	Hospitalisation for non-fatal myocardial infarction	134 (1·3%)	0·25 (0·21–0·30)	150 (1·4%)	0·27 (0·23–0·32)	0·92 (0·73–1·16)	0·48
	Hospitalisation for non-fatal stroke	129 (1·2%)	0·24 (0·20–0·29)	143 (1·3%)	0·26 (0·22–0·31)	0·93 (0·73–1·18)	0·54
	Vascular death	115 (1·1%)	0·22 (0·18–0·26)	108 (1·0%)	0·20 (0·16–0·24)	1·10 (0·84–1·43)	0·49
	All-cause death	437 (4·2%)	0·82 (0·74–0·90)	434 (4·1%)	0·79 (0·72–0·87)	1·04 (0·91–1·18)	0·59
	Hospitalisation or death from congestive heart failure	76 (0·7%)	0·14 (0·11–0·18)	99 (0·9%)	0·18 (0·15–0·22)	0·79 (0·59–1·07)	0·12

14 629 (69·3%) participants reported adherence to their assigned dosing time throughout the trial. Non-adherence to randomised dosing time at any time occurred in 6475 (30·7%) participants overall, with a mean time to first reported non-adherence to dosing time of 1·7 years (SD 1·6). Reported non-adherence to allocated dose timing at any point in the study was more common in those assigned to evening treatment than to morning treatment (4091 [39·0%] *vs* 2384 [22·5%]; p<0·0001). However, the last known status was reported non-adherence with allocated dosing time for only 2834 (13·4%) participants overall, 2084 (19·8%) in the evening dosing group and 750 (7·1%) in the morning dosing group. 617 (3·2%) participants reported that they had to change the time of day that a diuretic was administered (546 [5·2%] in the evening group *vs* 71 [0·7%] in the morning group; p<0·0001).

For the secondary endpoints of prespecified participant-reported adverse events, 1476 participants did not return a completed follow-up questionnaire and so were not included in these analyses. Participants in the evening dosing group were slightly less likely to report falls than those in the morning dosing group (2016 [21·1%] of 9574 *vs* 2235 [22·2%] of 10 054; p=0·048). Furthermore, we found no difference between the evening and morning dosing groups in the number of participants reporting no fractures (8930 [93·3%] *vs* 9369 [93·2%]), non-hospitalised fractures (572 [6·0%] *vs* 606 [6·0%]), or fractures that required a stay in hospital (72 [0·8%] *vs* 79 [0·8%] p=0·95). Additionally, in the intention-to-treat population, we found no difference in the number of participants reporting any glaucoma that required admission to hospital between evening and morning groups (44 [0·4%] *vs* 60 [0·6%]; p=0·15). In the intention-to-treat population, fewer participants in the evening dosing group reported one or more prespecified symptom adverse events during the study than did participants in the morning dosing group (7268 [69·2%] *vs* 7474 [70·5%]; p=0·041). Reported side effects are summarised in table 3. Dizziness or light-headedness, upset stomach or indigestion, diarrhoea, and muscle aches were all reported more commonly with morning dosing than with evening dosing. However, excessive visits to the toilet during the day or night and other non-specified adverse events were more commonly reported with evening dosing.

**Table 3:** Prespecified adverse events (symptoms) in safety analysis population (n=19 628)

	Evening dosing group (n=9574)*	Morning dosing group (n=10 054)*	**Between-group difference (95% CI)**†
Dizziness or light-headedness	3511 (36·7%)	4007 (39·9%)	−3·2% (−4·6 to −1·8)
Excessive visits to the toilet during the day or night	3825 (40·0%)	3660 (36·4%)	3·6% (2·2 to 4·9)
Sleep problems	4017 (42·0%)	4125 (41·0%)	0·9% (−0·5 to 2·3)
Upset stomach or indigestion	2639 (27·6%)	3050 (30·3%)	−2·8% (−4·1 to −1·5)
Diarrhoea	1803 (18·8%)	2170 (21·6%)	−2·8% (−3·9 to −1·6)
Feeling generally less well	3079 (32·2%)	3311 (32·9%)	−0·8% (−2·1 to 0·6)
Muscle aches	3724 (38·9%)	4352 (43·3%)	−4·4% (−5·8 to −3·0)
Other (not specified)	2970 (31·0%)	2686 (26·7%)	4·3% (3·0 to 5·6)

Numbers reported are the number of participants who indicated that they had experienced each prespecified symptom.

* Number of participants who submitted at least one completed study follow-up form.

† Difference in percentage: evening dosing group minus morning dosing group.

Of 11 470 participants who reported owning a home blood pressure monitor, 3844 (82·0%) of 5735 in the evening dosing group and 3813 (79·8%) of 5735 in the morning dosing group submitted at least one set of measurements. The modal times of blood pressure measurement were 0800–0900 h and 2200–2300 h (appendix p 12). Participants assigned to the evening dosing group had lower morning home blood pressure and higher evening home blood pressure than did those assigned to the morning dosing (appendix pp 5, 13–16). At all timepoints after randomisation, mean morning-assessed blood pressure was lower in the evening dosing group than in the morning dosing group (systolic blood pressure difference of 1·8 mm Hg lower [p<0·0001]; diastolic blood pressure difference of 0·4 mm Hg lower [p=0·023]). Conversely, evening-assessed blood pressure was lower in the morning dosing group than in the evening dosing group (systolic blood pressure difference of 1·1 mm Hg [p<0·0001]; diastolic blood pressure difference of 0·9 mm Hg [p<0·0001]; appendix p 5).

## Discussion

Nocturnal hypertension is an important predictor of adverse outcomes in people with hypertension, which has led to the hypothesis that taking antihypertensive medication in the evening might improve cardiovascular outcomes. However, this subject is not without controversy and has resulted in heated discussions.^8^, ^30^ The present study was sufficiently well powered to show a clinically important cardiovascular benefit with evening dosing compared with morning dosing; however, we found no such benefit. We found no advantage of evening versus morning dosing of antihypertensive medication with regard to major cardiovascular outcomes or mortality.

TIME was a large-scale pragmatic study, and so the findings probably reflect what would happen if patients in usual care were allocated to evening or morning dosing. All study participants were literate in information technology and had access to the internet. As voluntary clinical trial participants, they were also likely to be interested in their health and exhibit more positive health behaviours than the general population, as illustrated by the relatively low smoking rate. However, the baseline characteristics of the study population showed an incidence of comorbidities similar to patients with hypertension in a previous UK population-based study.^31^ Our overall primary event rate was lower than expected, which might be due to the healthy-participant effect but might also reflect decreasing rates of cardiovascular events in the general population.

Blood pressure measurements by home blood pressure machines showed significant but small differences between the randomised dosing groups. Antihypertensive treatment regimens were prescribed for participants by their usual treating clinician, and we have no reason to believe that the choice of treatment was affected by participation in the trial. Therefore, these findings show that most antihypertensive agents prescribed in UK usual care do not lower blood pressure evenly over 24 h.

Because event accrual was non-linear, due to the receipt of linked data in batches, predicting the date of achieving our target number of participants with primary outcome events was not simple. This fact, combined with delays in accessing linked data from NHS Digital and difficulty accessing NHS clinical records during periods of strain on the NHS due to the COVID-19 pandemic, meant that the number of accrued endpoints exceeded the minimum required for statistical purposes. The resulting 95% CI for the primary outcome excludes a benefit of more than 17%. Additionally, we calculated the endpoint target for the primary outcome only; therefore, comparisons in subgroups and secondary outcomes might be insufficiently powered to detect clinically meaningful differences between dosing times.

This trial design has some limitations that should be considered when interpreting our findings. First, the study used a prospective, randomised, open-label, blinded-endpoint design. All participants were aware of their allocated dosing time, which might have influenced behaviour and reporting. Moreover, participant-reported adverse events might be incomplete and subject to recall and reporting bias. Linked data and clinical source documentation corroborated participant-reported endpoint events. However, because prespecified adverse events were participant-reported only, they might be more subject to bias. In particular, higher rates of withdrawal from questionnaire follow-up in the evening dosing group than in the morning dosing group might have resulted in an underestimation of actual adverse event rates in between-group comparisons. Therefore, we believe that these self-reported prespecified adverse event data should be interpreted with caution. Additionally, home blood pressure measurements submitted manually to the study website might have been susceptible to recall bias and data entry errors.

Data have previously been published on the types of home blood pressure monitors owned, the factors associated with ownership, and the factors associated with long-term commitment to submitting home blood pressure measurements to the TIME study.^24^, ^25^, ^26^ Older participants, those with a positive family history of hypertension, those taking a higher number of antihypertensive medications, and those with less social deprivation were more likely to participate in providing home blood pressure measurements, whereas those with a higher BMI and who were smokers were less likely to provide home blood pressure measurements. Therefore, the home blood pressure data are not necessarily fully representative of the randomised population in the TIME study.

Differential non-adherence to dosing time might also affect our findings, but we have no reason to believe that overall medication non-adherence in the study would differ from that observed in other studies. Because most participants reported taking their antihypertensive medication in the morning before participating in the study, it is perhaps not surprising to note that those allocated to evening dosing were less likely to report remaining adherent to their allocated dosing time throughout the study period. The observed difference between the number of participants reporting ever non-adherence to allocated dosing time and final adherence status reflects a degree of switching back and forth between dosing times by some participants. This switching between adherence and non-adherence to dosing time might have been exacerbated by participants randomly assigned to morning dosing changing to evening dosing in response to high-profile media coverage of the results of the Hygia Chronotherapy Trial in the UK in October, 2019.^32^ However, the study team were aware that many of these participants reverted to morning dosing shortly afterwards (data not shown), after being informed that the TIME study independent data monitoring committee had recommended that the TIME study should continue after reviewing the study safety data.

Notably, the Canadian BedMed trial,^33^ which is assessing whether bedtime antihypertensive administration reduces major adverse cardiovascular events compared with conventional morning use, and the associated BedMed-frail trial, which includes several secondary safety outcomes of relevance to a frail older population, are both continuing (NCT04054648).

Finally, the TIME study was not a study of nocturnal hypertension or other disorders of diurnal blood pressure variation, and further research is needed to advise on dosing time in those populations.

In this pragmatic study, reflecting usual care, allocation to evening dosing of usual antihypertensive medication did not improve the primary composite endpoint of vascular death or hospitalisation for non-fatal myocardial infarction or non-fatal stroke compared with morning dosing. Taking medication in the evening was not harmful but provided no additional benefit versus morning dosing. Therefore, patients should be advised that they need not change their antihypertensive medication dosing time but might choose to take their medication at a time that suits them best, because the timing makes no difference to cardiovascular outcomes.

## Data sharing

Access to a de-identified participant dataset and data dictionary is available upon reasonable request to researchers who provide a methodologically sound proposal, with no prespecified restrictions on data use. Any such requests should be sent to the corresponding author for consideration by the trial steering committee. There might be restrictions on sharing data derived from record-linkage to NHS datasets. A period of 18 months after publication of the main study results should elapse before requests are made, to allow the authors to publish substudies and further analyses.

## Declaration of interests

ISM reports research grants from Menarini, EMA, Sanofi, HDR UK, National Institute for Health and Care Research (NIHR) Health Technology Assessment (HTA), and Innovative Medicines Initiative outside of the submitted work; institutional consultancy income from AstraZeneca outside of the submitted work; and personal income from AstraZeneca and Amgen outside of the submitted work. TMM reports grants from Menarini/Ipsen/Teijin, NIHR HTA, and MSD outside of the submitted work; personal income for consultancy from Novartis and AstraZeneca outside of the submitted work; and is a trustee of the Scottish Heart Arterial Risk Prevention (SHARP) Society. NRP reports receiving financial support from several pharmaceutical companies that manufacture blood pressure lowering agents for consulting (Servier and Aktiia), research projects and staffing (Servier and Pfizer), and for arranging and speaking at educational meetings (Servier, Sanofi, Eva Pharma, Pfizer, Emcure India, and Dr Reddy's Laboratories); he holds no stocks or shares in any of these companies. BW is supported by the NIHR University College London Hospitals Biomedical Research Centre; reports research grants from Omron, MRC, and NIHR; and reports honoraria from Daiichi Sankyo, Omron, Servier, Pfizer, Novartis, and Menarini outside of the submitted work. MJB is supported by the NIHR Barts Health Biomedical Research Centre and reports research grants from the British Heart Foundation, MRC, and NIHR. PMR declares consultancy income from Abbot and Bayer, and honoraria from Sanofi; he is also a member of the AXIOMATIC trial Data Monitoring Committee. CCL reports membership of a data safety monitoring board or steering committee for Novo Nordisk. AR reports an unpaid membership of an NIHR steering committee. AM reports a British Heart Foundation grant to his university to support salary costs. All other authors declare no competing interests.
