# Supplementary material for: Cardiovascular outcomes in adults with hypertension with evening versus morning dosing of usual antihypertensives in the UK (TIME study): a prospective, randomised, open-label, blinded-endpoint clinical trial
Source: Lancet. 2022 Oct 22;400(10361):1417–25. doi: 10.1016/S0140-6736(22)01786-X (PMC9631239; doi:10.1016/S0140-6736(22)01786-X)
Supplement: Supplementary appendix [file mmc1.pdf]

THE LANCET

Supplementary appendix

This appendix formed part of the original submission and has been peer reviewed. We post it as supplied by the authors.

Supplement to: Mackenzie IS, Rogers A, Poulter NR, et al. Cardiovascular outcomes in adults with hypertension with evening versus morning dosing of usual antihypertensives in the UK (TIME study): a prospective, randomised, open-label, blinded-endpoint clinical trial. *Lancet* 2022; published online Oct 11. https://doi.org/10.1016/S0140-6736(22)01786-X.

THE LANCET

This appendix formed part of the original submission and has been peer reviewed. We post it as supplied by the authors.

Supplementary Material

Cardiovascular outcomes in adults with hypertension allocated to evening or morning dosing of usual antihypertensives: a prospective, randomised, open-label, blinded-endpoint clinical trial. The TIME study.

Mackenzie IS, Rogers A, Poulter N, Williams B, Brown MJ, Webb DJ, Ford I, Rorie D, Guthrie G, Grieve JWK, Pigazzani F, Rothwell PM, Young R, McConnachie A, Struthers AD, Lang C, MacDonald TM on behalf of the TIME study group.

Contents

Supplementary Table S1: Antihypertensive use at study entry by dosing time group 3

Supplementary Table S2: Reasons for withdrawal....... 4

Supplementary Table S3: Study averaged blood pressure measurements....... 5

Supplementary Figure S1: Prespecified subgroup analyses for the primary outcome. 6

Supplementary Figure S2: Cumulative incidence function for hospitalisation due to non-fatal myocardial infarction. Intention-to-treat analysis (n = 21 104)....... 7

Supplementary Figure S3: Cumulative incidence function for hospitalisation due to non-fatal stroke. Intention-to-treat analysis (n = 21 104). 8

Supplementary Figure S4: Cumulative incidence function for vascular death. Intention-to-treat analysis (n = 21 104).9

Supplementary Figure S5: Cumulative incidence function for all-cause mortality. Intention-to-treat analysis (n = 21 104)....... 10

Supplementary Figure S6: Cumulative incidence function for hospitalisation or death due to congestive heart failure. Intention-to-treat analysis (n = 21 104)....... 11

Supplementary Figure S7: Distribution of participant-reported time of home blood pressure measurement 12

Supplementary Figure S8: Morning-assessed Systolic Blood Pressure....... 13

Supplementary Figure S9: Morning-assessed Diastolic Blood Pressure....... 14

Supplementary Figure S10: Evening-assessed Systolic Blood Pressure....... 15

Supplementary Figure S11: Evening-assessed Diastolic Blood Pressure 16

TIME Study Committees and Contributors 17

Study Protocol....... 19

Endpoint Committee Charter 47

Independent Data Monitoring Committee Charter....... 98

Statistical Analysis Plan....... 111

Supplementary Table S1: Antihypertensive use at study entry by dosing time group

	Evening (n=3693)	Morning (n=4163)
Angiotensin-converting enzyme inhibitors	1739 (47·1%)	1972 (47·3%)
Calcium-channel blockers	1395 (37·8%)	1601 (38·4%)
Angiotensin receptor blockers	940 (25·5%)	976 (23·4%)
Thiazide and related diuretics	712 (19·3%)	858 (20·6%)
Beta-adrenoceptor blockers	356 (9·6%)	392 (9·4%)
Alpha-adrenoceptor blockers	184 (5·0%)	227 (5·5%)
Other diuretics	89 (2·4%)	107 (2·6%)
Other	117 (3·2%)	141 (3·4%)

The data are derived from a medication questionnaire introduced during the study recruitment; it was not available for completion at baseline for all participants.

Supplementary Table S2: Reasons for withdrawal

	All (n = 2453)	Evening (n = 1539)	Morning (n = 914)
Reason given for withdrawal*			
Found taking tablets at randomised time inconvenient	304 (12·4%)	253 (16·4%)	51 (5·6%)
Forgot to take tablets at randomised time	195 (7·9%)	163 (10·6%)	32 (3·5%)
Found blood pressure to be less well controlled	82 (3·3%)	66 (4·3%)	16 (1·8%)
Bored/fed up taking tablets at randomised time	26 (1·1%)	17 (1·1%)	9 (1·0%)
Taking a lot of tablets for other conditions and found difficult to take blood pressure tablets at other times	149 (6·1%)	119 (7·7%)	30 (3·3%)
No reason given	547 (22·3%)	450 (29·2%)	97 (10·6%)
Withdrawal preference*			
Withdrawal from follow-up questionnaires	1232 (50·9%)	748 (49·4%)	484 (53·4%)
Complete withdrawal (including record-linkage)	847 (34·5%)	529 (34·4%)	318 (34·8%)

*Categories not mutually exclusive

Supplementary Table S3: Study averaged blood pressure measurements

	Evening dosing (n=3844)	Morning dosing (n=3813)	Difference*	t-test p-value
**Morning-assessed blood pressure**				
SBP, mmHg	130·54 (11·02)	132·31 (11·06)	-1·77	<0·0001
DBP, mmHg	77·15 (8·12)	77·58 (8·18)	-0·43	0·02
**Evening-assessed blood pressure**				
SBP, mmHg	133·19 (11·23)	132·13 (11·28)	+1·06	<0·0001
DBP, mmHg	77·11 (7·85)	76·26 (8·03)	+0·85	<0·0001

*Difference in means, Evening dosing group minus Morning dosing group, mmHg

Supplementary Figure S1: Prespecified subgroup analyses for the primary outcome.

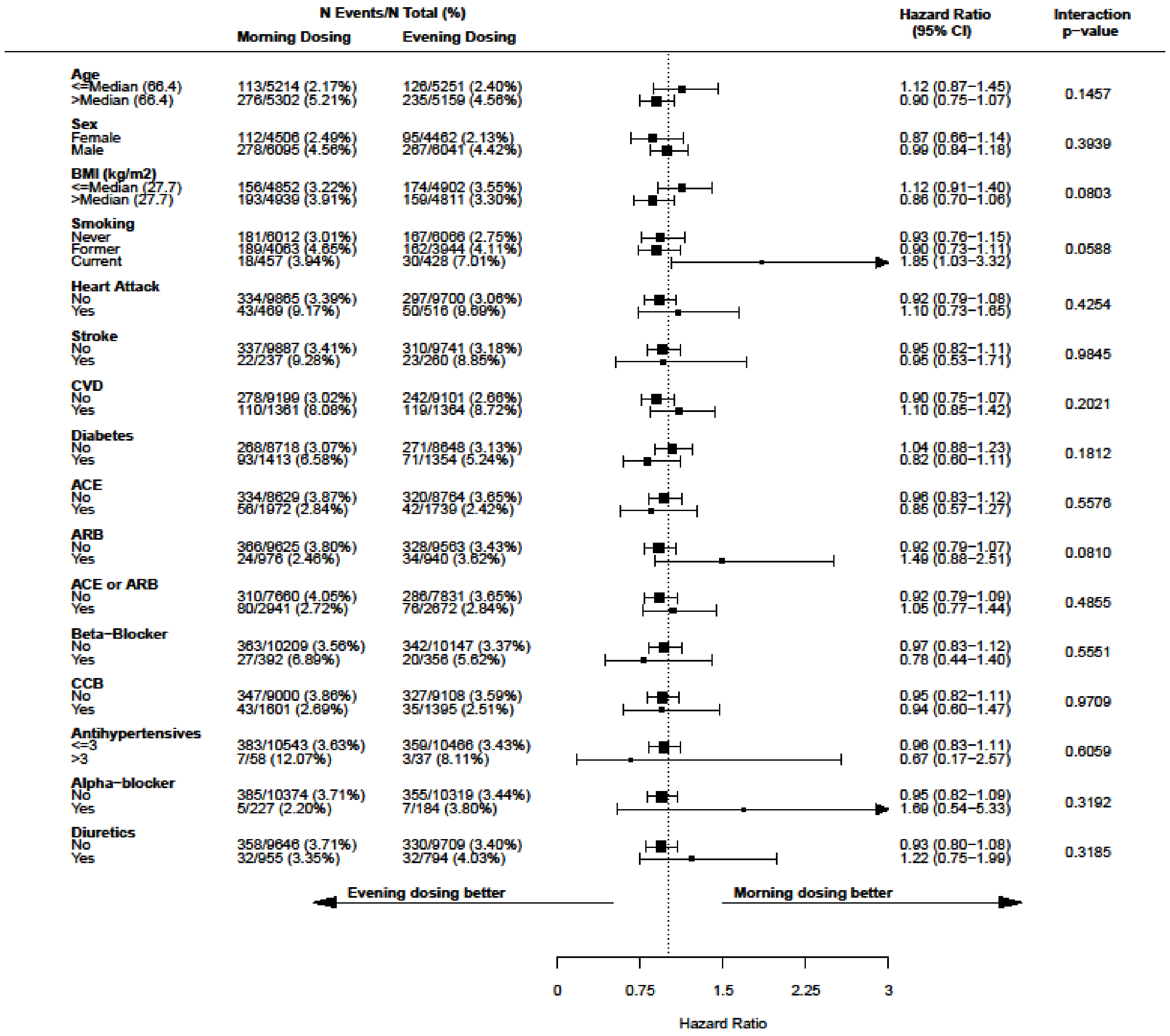

Intention-to-treat analysis (n= 21 104). Forest plot shows the HRs and 95% CIs separately for each outcome. HR=hazard ratio. C BMI = body mass index. CVD = cardiovascular disease. ACE = angiotensin-converting enzyme inhibitor. ARB = angiotensin II receptor blocker. CCB = calcium channel blocker.

Supplementary Figure S2: Cumulative incidence function for hospitalisation due to non-fatal myocardial infarction. Intention-to-treat analysis (n = 21 104).

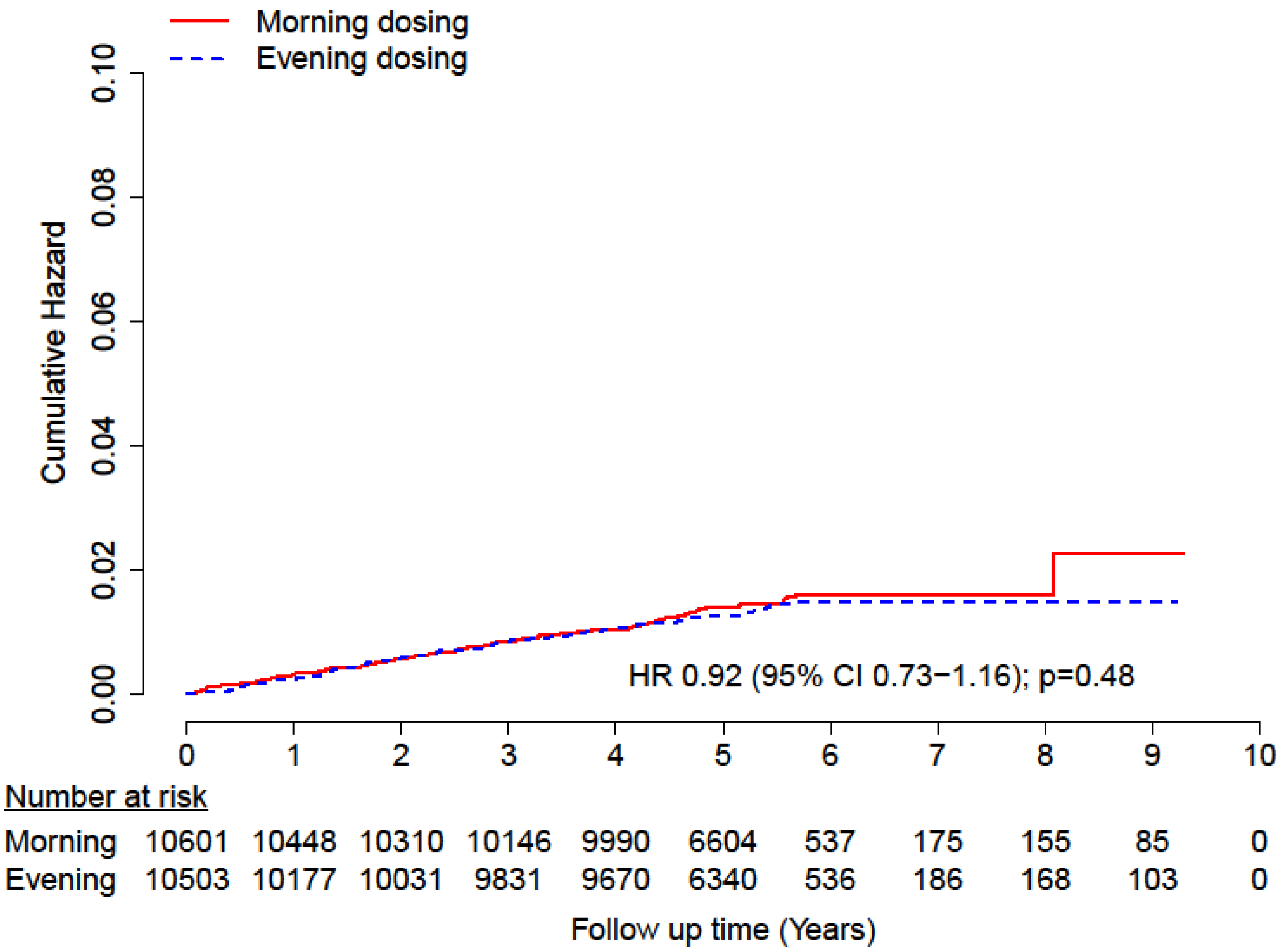

Supplementary Figure S3: Cumulative incidence function for hospitalisation due to non-fatal stroke. Intention-to-treat analysis (n = 21 104).

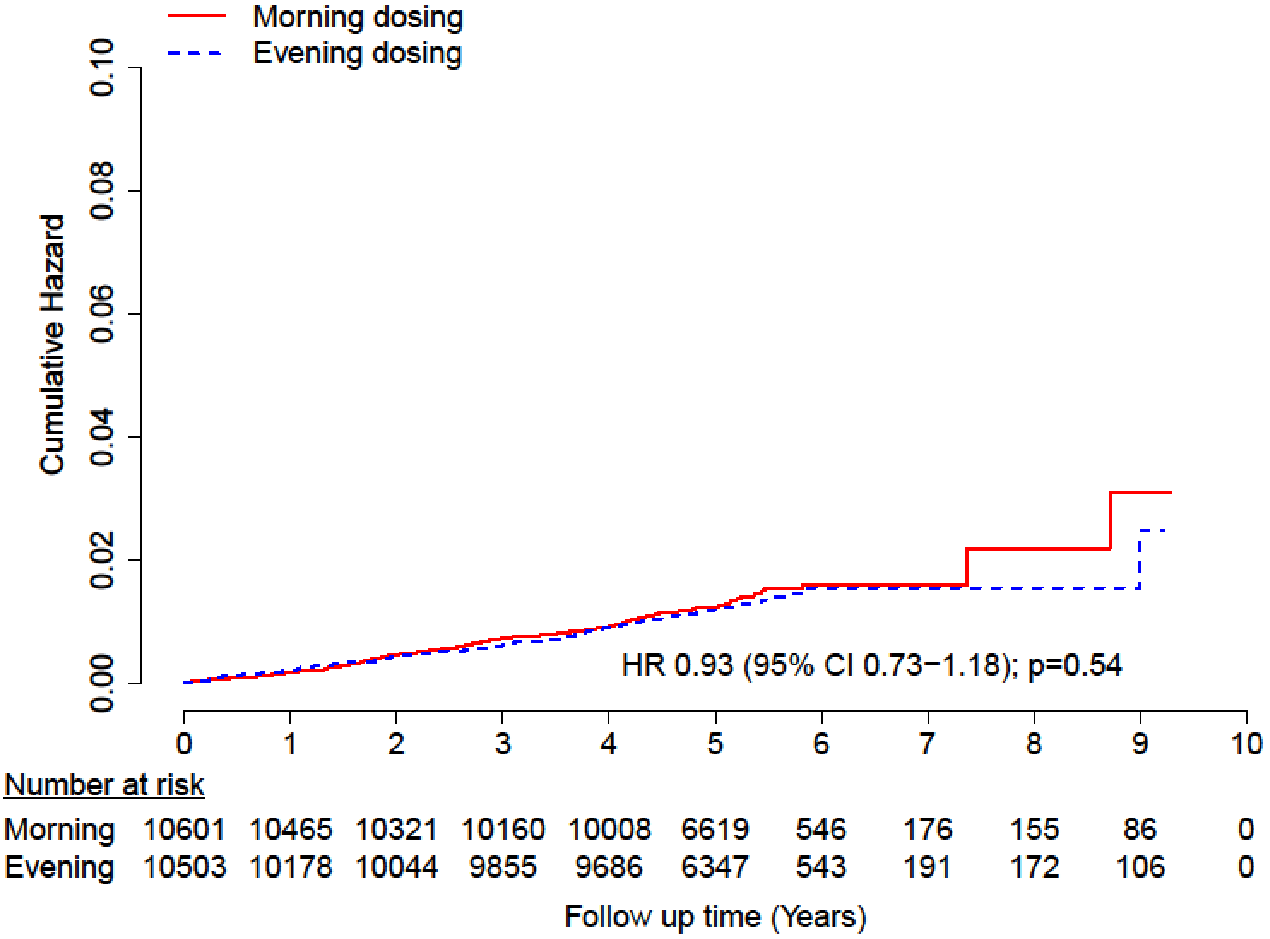

Number at risk	0	1	2	3	4	5	6	7	8	9	10
Morning	10601	10465	10321	10160	10008	6619	546	176	155	86	0
Evening	10503	10178	10044	9855	9686	6347	543	191	172	106	0

Supplementary Figure S4: Cumulative incidence function for vascular death. Intention-to-treat analysis (n = 21 104).

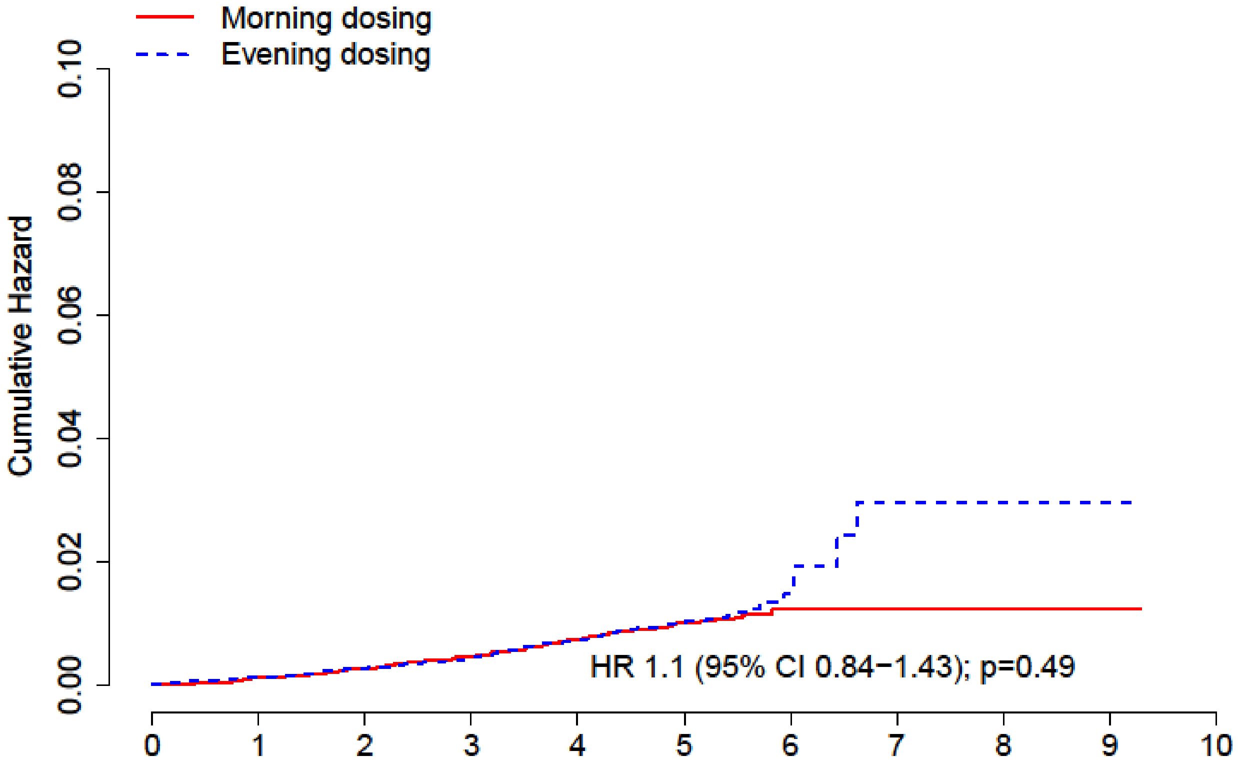

Number at risk	0	1	2	3	4	5	6	7	8	9	10
Morning	10601	10482	10369	10232	10095	6698	551	176	156	86	0
Evening	10503	10199	10088	9912	9767	6418	550	193	174	108	0

Follow up time (Years)

Supplementary Figure S5: Cumulative incidence function for all-cause mortality. Intention-to-treat analysis (n = 21 104).

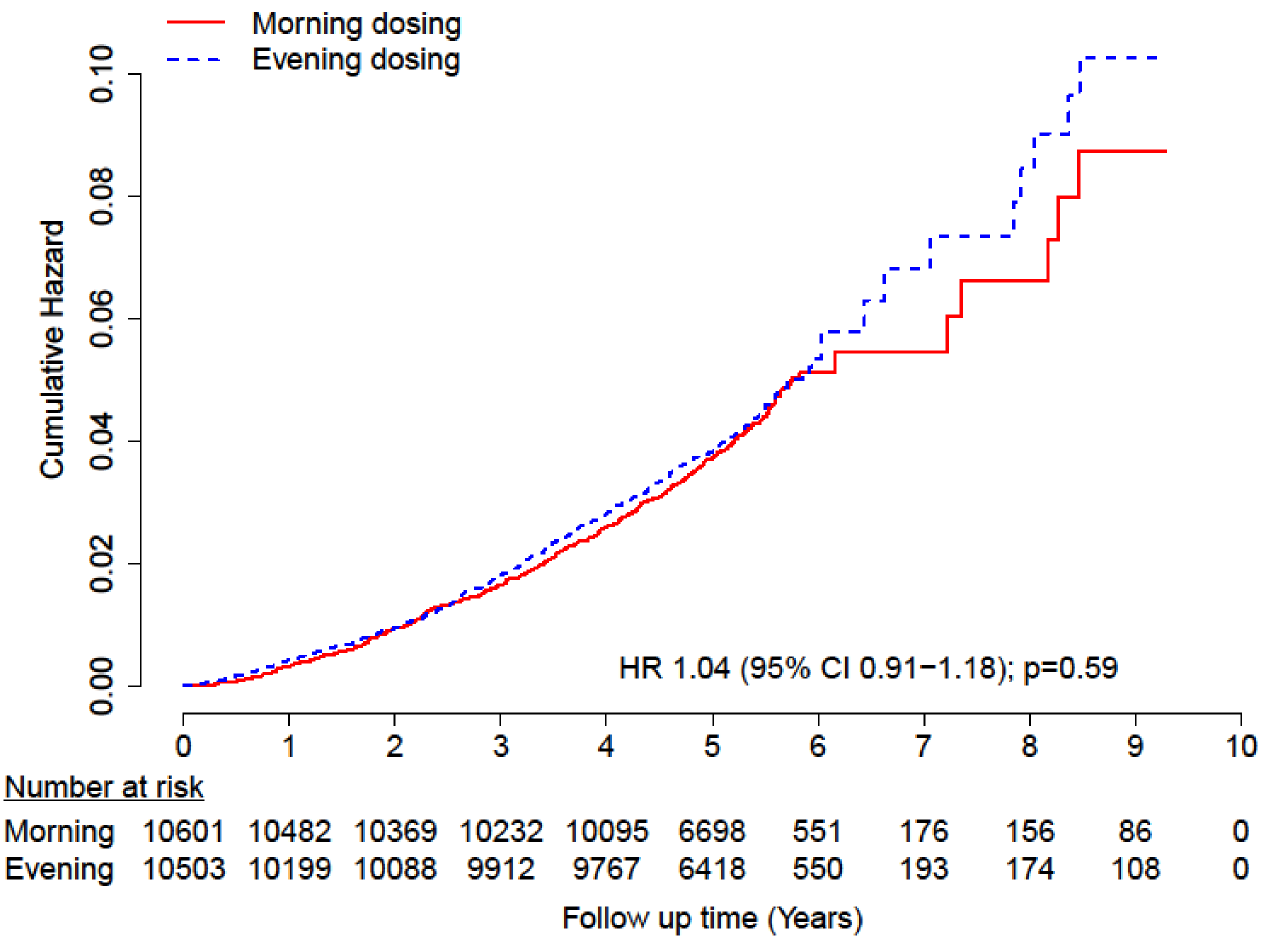

Supplementary Figure S6: Cumulative incidence function for hospitalisation or death due to congestive heart failure. Intention-to-treat analysis (n = 21 104).

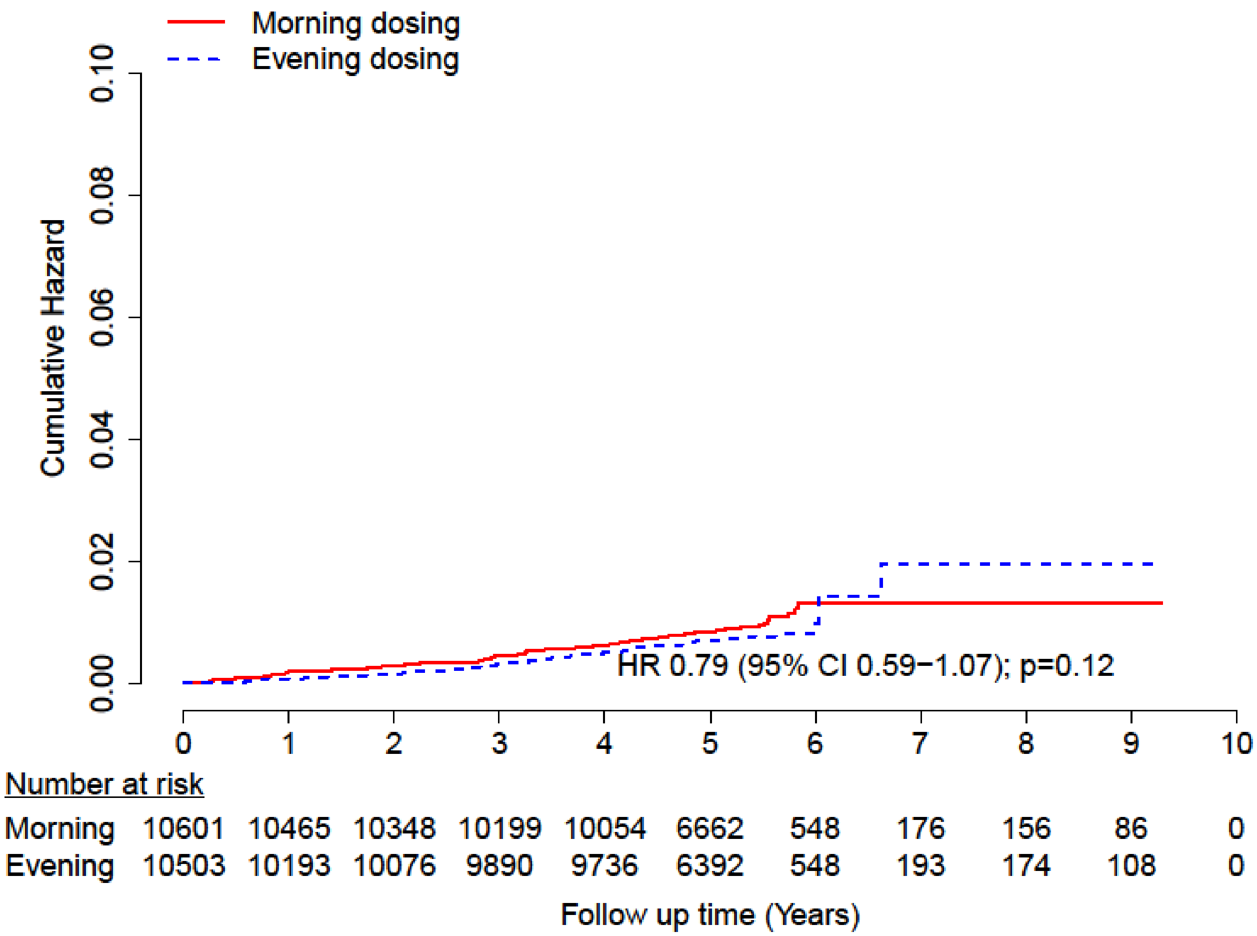

Supplementary Figure S7: Distribution of participant-reported time of home blood pressure measurement

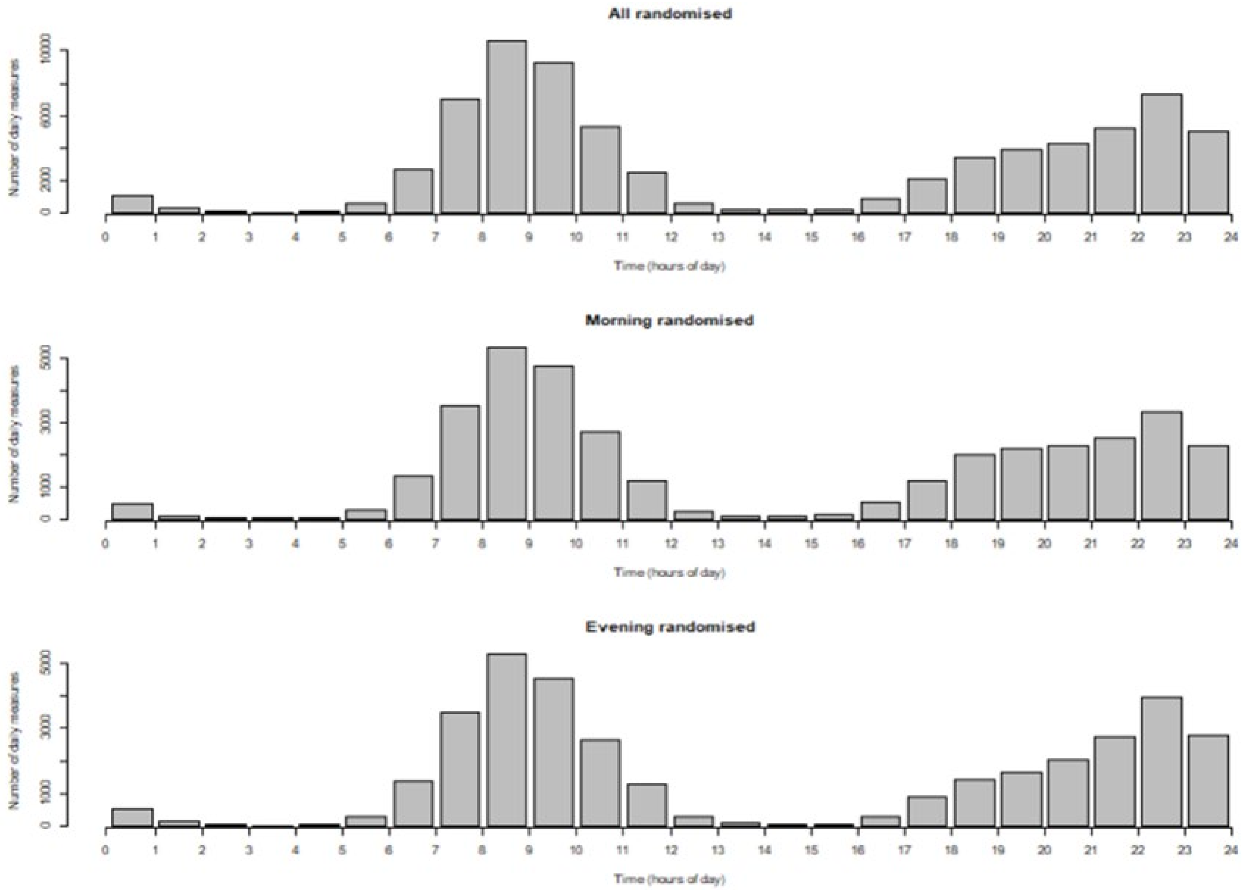

Supplementary Figure S8: Morning-assessed Systolic Blood Pressure

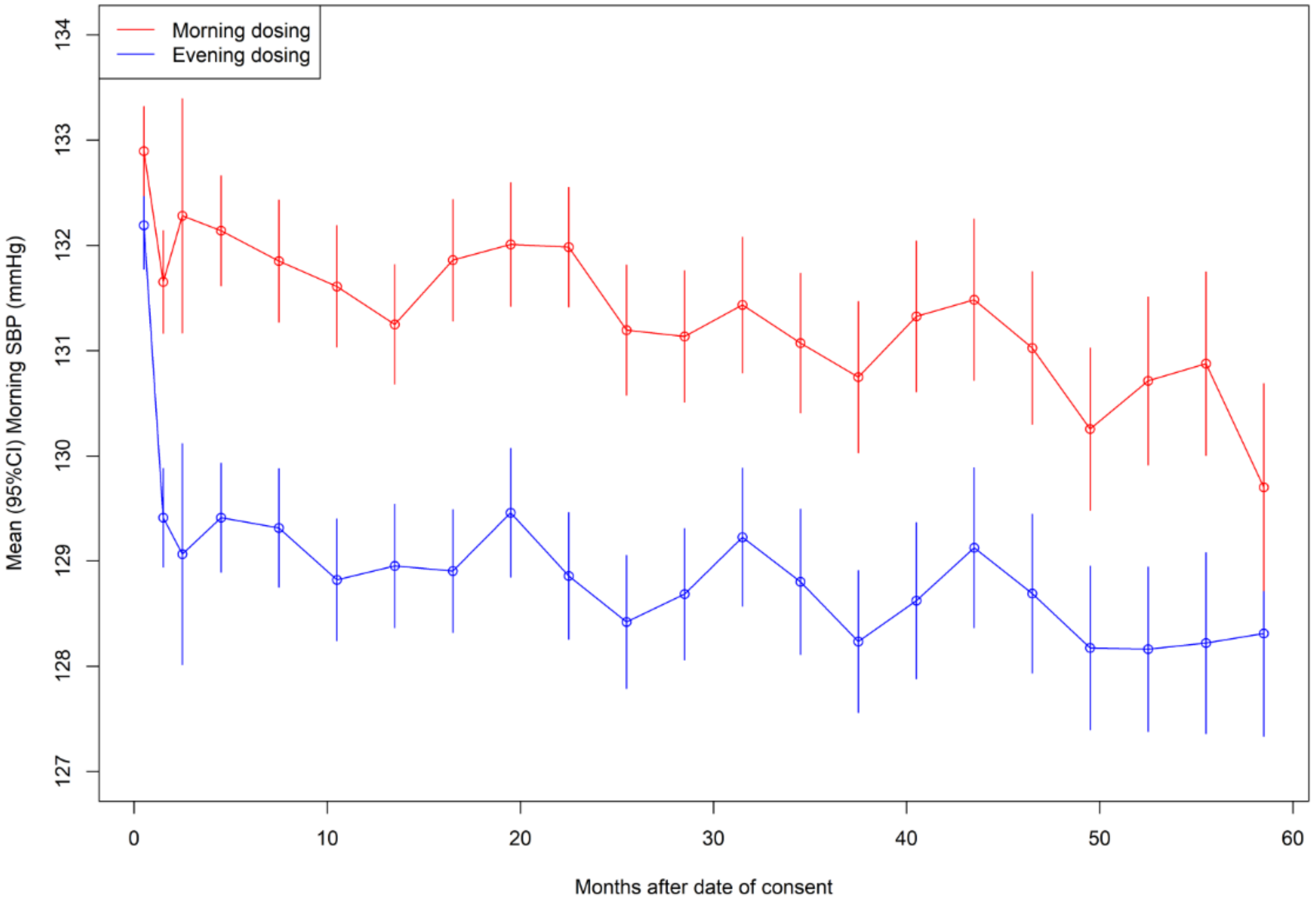

Supplementary Figure S9: Morning-assessed Diastolic Blood Pressure

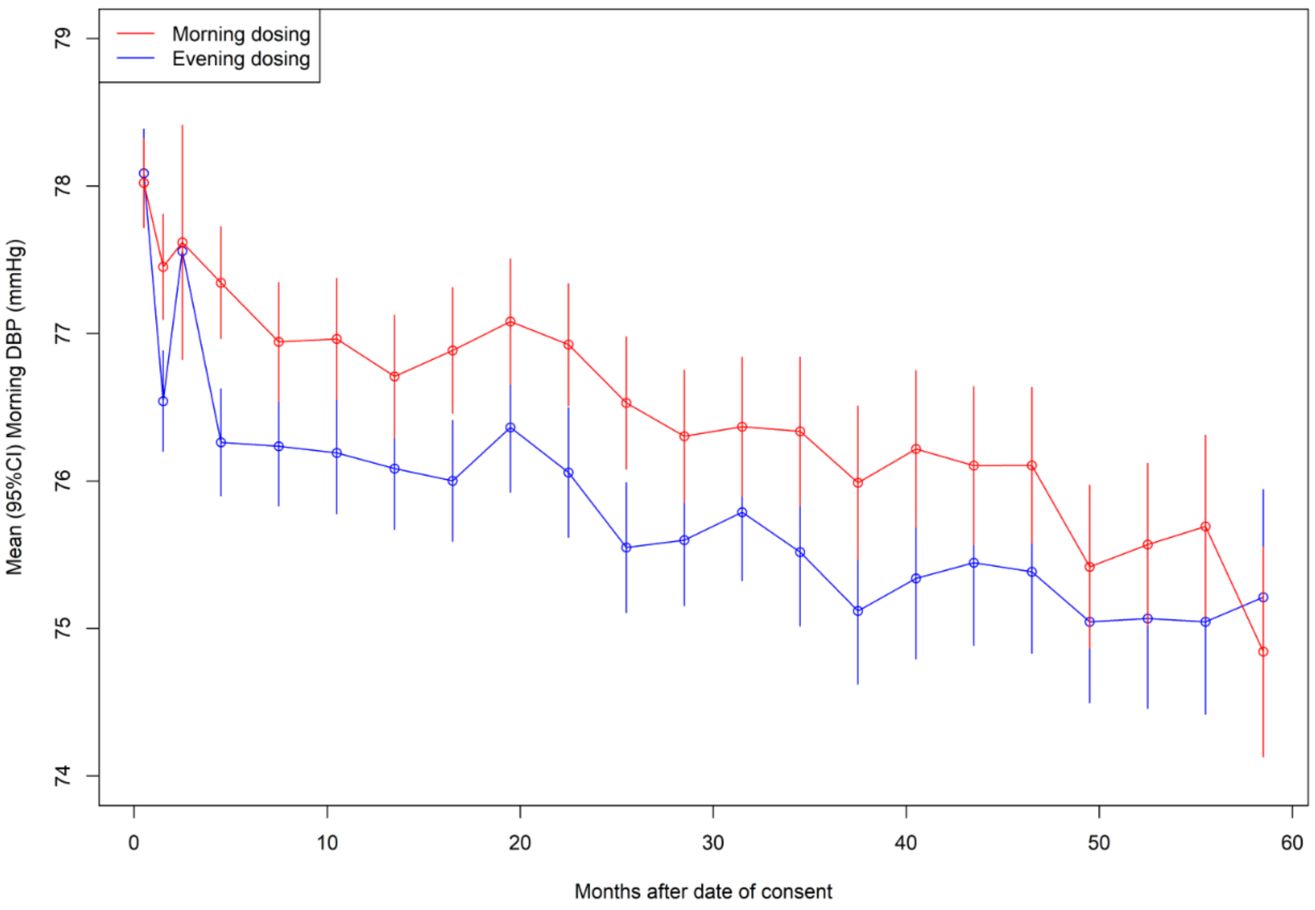

Supplementary Figure S10: Evening-assessed Systolic Blood Pressure

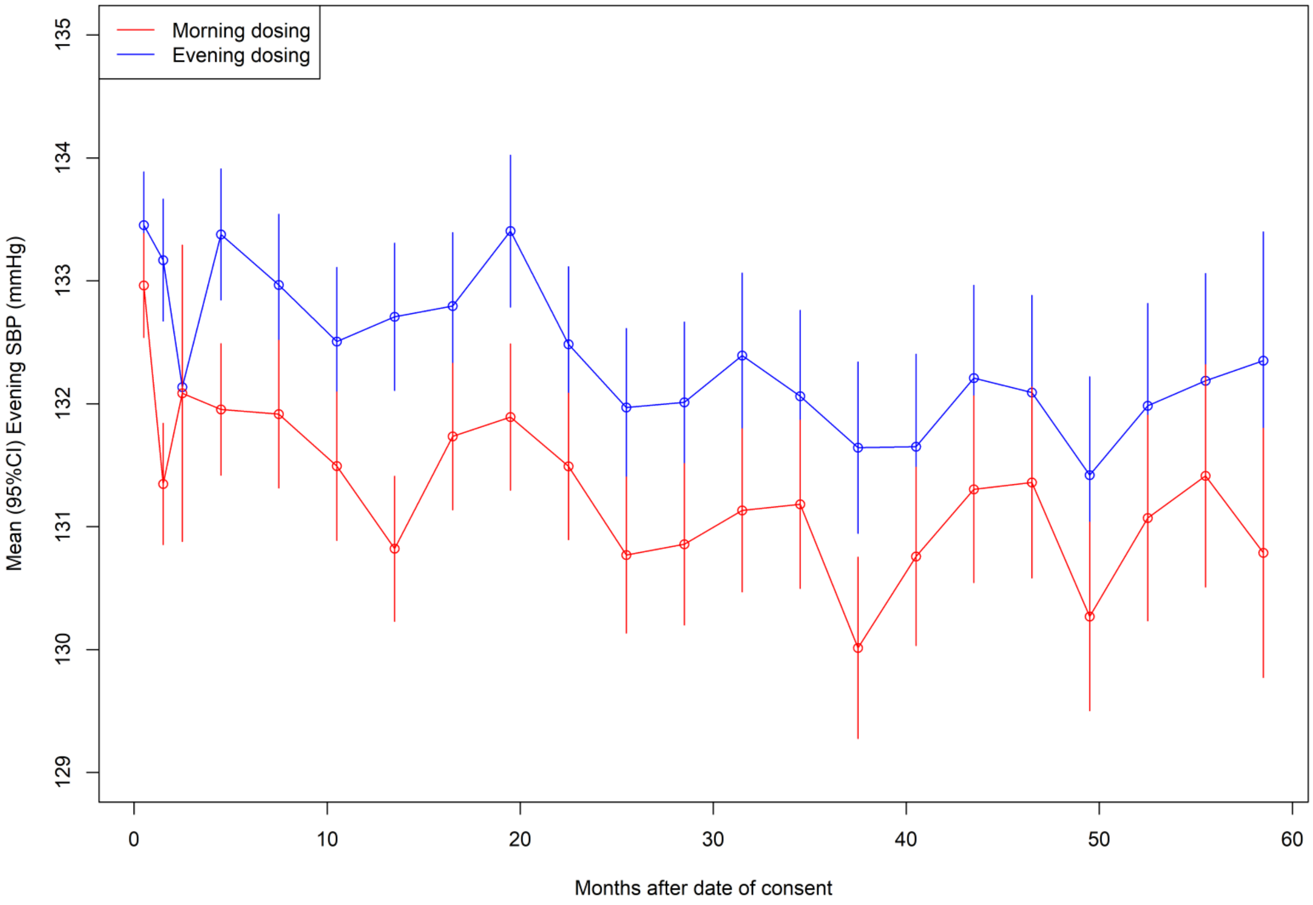

Supplementary Figure S11: Evening-assessed Diastolic Blood Pressure

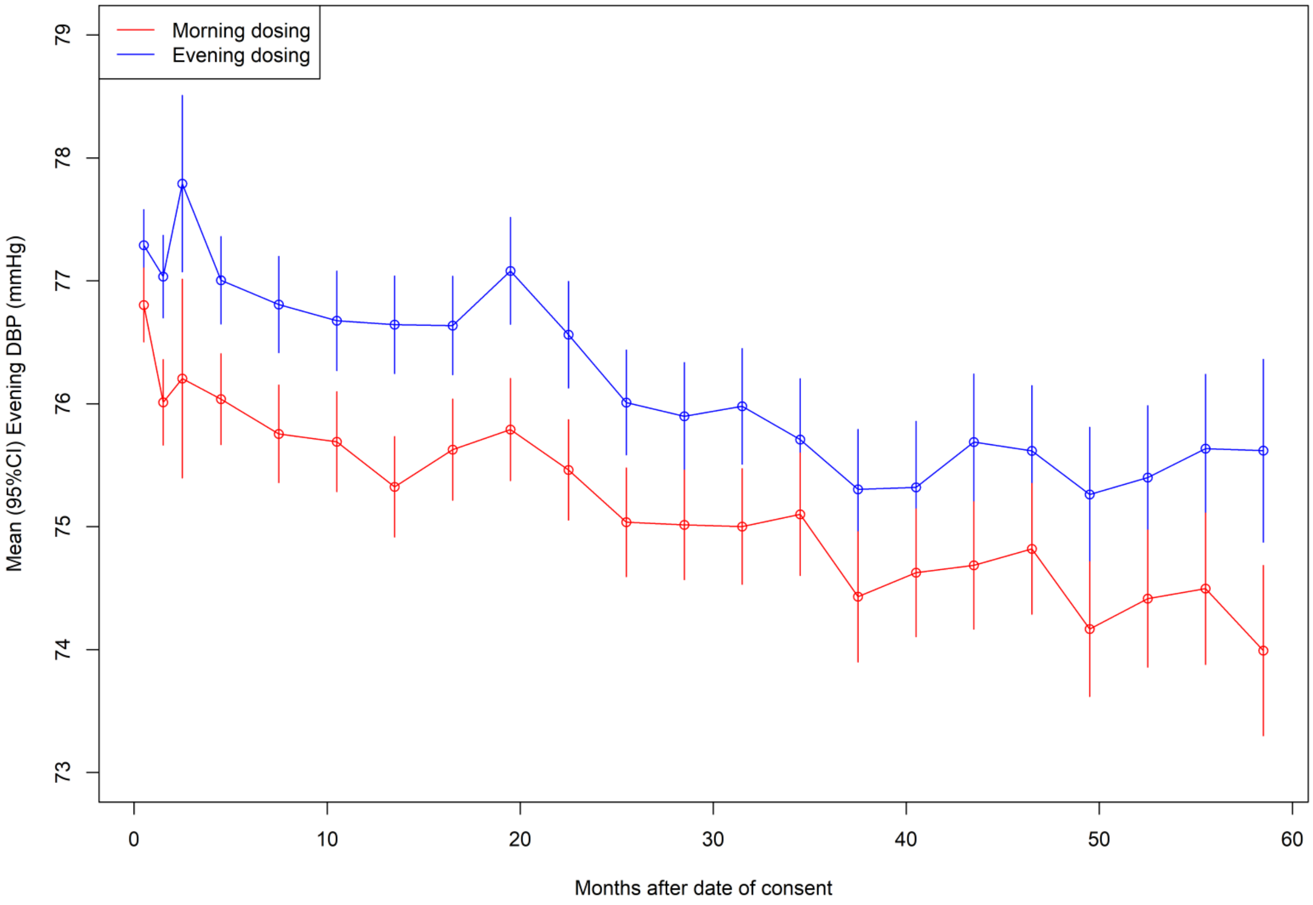

TIME Study Committees and Contributors

**TIME Steering Committee**	
Prof Neil Poulter (Chair)	Imperial College London
Prof Tom MacDonald (Chief Investigator)	University of Dundee
Prof Morris Brown	Queen Mary University of London
Evelyn Findlay	University of Dundee
Prof Ian Ford	Robertson Centre for Biostatistics, University of Glasgow
Prof Chim Lang	University of Dundee
Prof Isla Mackenzie	University of Dundee
Prof David Webb	University of Edinburgh
Prof Bryan Williams	University College London
**TIME Independent Data Monitoring Committee**	
Prof Peter Sever (Chair)	Imperial College London
Prof Francesco Cappuccio	University of Warwick
Prof Stuart Pocock	London School of Hygiene and Tropical Medicine
Prof Kausik Ray	Imperial College London
**TIME Endpoint Adjudication Committee**	
Prof Chim Lang (Chair)	University of Dundee and NHS Tayside
Dr Zaid Iskandar	University of Dundee and NHS Tayside
Dr Ali Kashan	University of Dundee and NHS Tayside
Dr Ify Mordi	University of Dundee and NHS Tayside
Dr Priya Nair	University of Dundee and NHS Tayside
Dr Ben New	University of Dundee and NHS Tayside
Dr Mon Myat Oo	University of Dundee and NHS Tayside
Dr Jagdeep Singh	University of Dundee and NHS Lothian
Dr Qaiser Zeb	University of Dundee and NHS Lothian
**Software development and data management**	University of Dundee
Dr David Rorie	
Lewis McConnachie	
**Statistics**	Robertson Centre for Biostatistics, University of Glasgow
Dr Robin Young	
Prof Alex McConnachie	
Prof Ian Ford	
**MEMO Research, University of Dundee**	
**Project Management**	**Study Physicians**
Wendy Saywood, Rebecca Barr, Suzanne Duce, Geraldine Mackle, Lindyanne Alexander, Evelyn Findlay	Amy Rogers, Greg Guthrie, Filippo Pigazzani, Kerr Grieve, Evie Rooke, Selvarani Subbarayan, Alex Doney
**Study Administration**	**Research Nurses**
Samantha Thomson, Catriona Young, Heather Farmer, Dawn Thompson, Vivian Taylor, Julia Carr, Carolyn Boyle, Maya Prakash	Dawn Ross, Lesley Riley, Ann Mackintosh, Caroline Paterson, Alison McGinnis
**Others**	**Patient and Public Involvement Group**
Joanne Elwin, Shirley Fraser, Susan Long, Patrick McDonnell, Caitlin McKay, Ruth Normand, Sheelagh Wiseman, Krists Zutis	Sheina Bell, Sandy Edwards, Sharon Ham, Irene and Stephen Vincent
**TASC, University of Dundee and NHS Tayside**	
**Governance and Contracts**	
Patricia Burns, Graeme Boyle, Jacob George, Russell Petty, Euan Banyard	
**Recruitment Support**	
**England**	
**NIHR**	
Lead Clinical Research Network (CRN): West Midlands (Primary Care Research Manager, Max Feltham)	
Participating CRNs: East Midlands, North Midlands, North Thames (NOCLOR), Eastern, Thames Valley and South Midlands, Wessex; Kent, Surrey, and Sussex; North West Coast, Yorkshire and Humber, South West Peninsula, London, North East.	

Pharmacy Recruitment: West Midlands (Research Facilitator, Mark Evans)
Recruiting Hospitals/Trusts: Ashfield and St Peter's Hospitals, Surrey; Barts Health NHS Trust; Basildon and Thurrock University Hospitals NHS Foundation Trust; Birmingham South Central CCG; Blackpool Teaching Hospital; Bradford Royal Infirmary; Braintree Community Hospital; Bridgwater Community Hospital; Broomfield Hospital, Chelmsford; Burnham-on-Sea Memorial Hospital; Burnley General Hospital; Calderdale and Huddersfield NHS Trust; Chard Community Hospital; Crewkerne Community Hospital; Dene Barton Community Unit; Frome Community Hospital; Harrogate & District NHS Foundation Trust; Hinchinbrooke Hospital; Homerton Hospital, Hackney; King George Hospital, Essex; Leeds General Infirmary; Lister Hospital, Stevenage Herts; Luton and Dunstable Hospital; Maidstone Hospital, Kent; Mid Essex Hospital Services; Minehead Community Hospital; North Cumbria Unversity Hospital; North Manchester General Hospital; North Middlesex Univerity Hospital; Northampton General Hospital NHS Trust; Plymouth Community Healthcare; Plymouth Hospitals NHS Trust Princess Alexandra Hospital, Harlow; Queen's Hospital, Romford; RNLN North Cumbria Integrated Care NHS Trust; Royal Blackburn Hospital; Royal Cornwall Hospital; Royal Devon and Exeter NHS Foundation Trust; Royal Free Hospital; Salford Royal NHS Foundation Trust; Shepton Mallet Community Hospital; Somerset Partnership NHS Foundation Trust; South Devon Healthcare NHS Foundation Trust; South Petherton Community Hospital; Southend University Hospital NHS Foundation Trust; St Luke's Hospital, Bradford; St Michael's Hospital, Braintree; St Peter's Hospital, Maldon; Stockport NHS Foundation Trust; Taunton and Somerset NHS Foundation Trust; The Whittington Hospital NHS Trust; Torbay and South Devon NHS Foundation Trust Hospital; University College London Hospitals; University Hospital of North Tees; University Hospital of South Manchester NHS Foundation Trust; Victoria Hospital, Deal; Watford General Hospital; Wellington Community Hospital; West Cumberland Hospital, Whitehaven and Cumberland Infirmary, Carlisle; West Hertfordshire Hospitals NHS Trust; West Mendip Community Hospital; West Middlesex University Hospital Trust; West Suffolk Foundation Trust; Whitstable and Tankerton Hospital; Williton Community Hospital; Wincanton Community Hospital; Yeovil District Hospital NHS Foundation Trust
**Scotland**
**Scottish Primary Care Research Networks (SPCRN)**
Recruiting hospitals/trusts: Ninewells Hospital, NHS Tayside
**Wales**
Health and Care Research Wales (Primary Care Research Manager, Lewis Darmanin)
Local Health Boards: Aneurin Bevan University Health Board, Betsi Cadwaladr University Health Board, Cardiff & Vale University Health Board, Cwm Taf, Morgannwg University Health Board, Hywel Dda University Health Board, Powys Teaching Health Board, Swansea Bay University Health Board

Study Protocol

Study Protocol

Treatment in Morning versus Evening Study (TIME)

A British Hypertension Society Research Network Study

Funded by the British Heart Foundation

Sponsor	University of Dundee
Trial Coordinating Centre	Medicines Monitoring Unit & Hypertension Research Centre, Ninewells Hospital & Medical School, Dundee DD1 9SY
Funder	British Heart Foundation
Funding Reference Number	CS/14/1/30659
Chief Investigator	Professor Tom MacDonald
Sponsor's Protocol Code Number	Pilot Code; V1.0200411
CTA Number	N/A
MREC Number	11/AL/0309
ISRCTN Number	ISRCTN18157641
UKCRN Number	17071
IRAS Study Number	80516
Version Number and Date	Version 12.0 (10/02/2021)

Contents

CONTENTS......3
TREATMENT IN MORNING VERSUS EVENING (TIME)......5
LIST OF ABBREVIATIONS......6
1 SUMMARY......7
2 INTRODUCTION......7
2.1 BACKGROUND......7
2.2 SCIENTIFIC PRINCIPLES......8
2.3 HYPOTHESIS......8
2.4 QUESTIONS TO BE ADDRESSED......8
2.5 PILOT STUDY......8
3 STUDY OBJECTIVES......9
3.1 PRIMARY OBJECTIVE......9
3.2 METHODS......9
STUDY DESIGN......9
3.4 STUDY POPULATION......10
4 INCLUSION CRITERIA......10
4.1 EXCLUSION CRITERIA......10
5 OUTCOME MEASURES......11
5.1 PRIMARY ENDPOINT......11
5.2 SECONDARY ENDPOINTS......11
5.3 END POINT ADJUDICATION......11
6 PARTICIPATION SELECTION AND ENROLMENT......12
6.1 RECRUITMENT STRATEGY......12
6.2 STUDY TIMELINE......13
6.3 CONSENTING PARTICIPANTS......13
SCREENING FOR ELIGIBILITY......14
7 RANDOMISATION......14
7.1 COMPUTER RANDOMISATION......14
7.2 TREATMENT ALLOCATION......14
8 DIURETIC TREATMENT: PATIENT INSTRUCTIONS......15
8.1 THIAZIDE OR THIAZIDE-LIKE DIURETICS......15
8.2 DIURETIC THERAPY (WITH LOOP-TYPE DIURETICS) AND EVENING DOSING......15
9 WITHDRAWAL......15
10 MEDICINAL PRODUCTS......16
10.1 PARTICIPANT COMPLIANCE......16
10.2 OTHER MEDICATIONS......16
11 DATA COLLECTION......16
11.1 DATA STORAGE......17
11.2 DATA RETENTION......17
11.3 DATA ACCESS......17
12 STATISTICS AND DATA ANALYSIS......17
12.3 BIAS......17
POTENTIAL RISKS & HAZARDS......17

SAMPLE SIZE 17
12.4 PLANNED SUBGROUP ANALYSES 18
13 ADVERSE EVENTS 18
13.1 EARLY STOPPING 19
14 COMPETING STUDIES 19
15 TRIAL MANAGEMENT AND OVERSIGHT ARRANGEMENTS 19
15.1 SPONSORSHIP 19
15.2 TRIAL EXECUTIVE COMMITTEE 19
15.3 OWNERSHIP OF STUDY DATABASE 19
15.4 POTENTIAL SUB-STUDIES 20
16 SUB-STUDIES 21
16.1 COGNITIVE FUNCTION SUB-STUDY 21
16.2 SLEEP SUB-STUDY 21
16.3 MOOD SUB-STUDY 21
16.4 GENETICS SUB-STUDY (POTENTIAL) 21
16.5 CHRONOTYPE SUB-STUDY 21
17 TRIAL MANAGEMENT 21
17.1 CENTRAL TRIAL OFFICE 22
17.2 TRIAL STEERING COMMITTEE 22
18 INDEPENDENT DATA MONITORING COMMITTEE 22
19 INSPECTION OF RECORDS 22
19.1 STUDY MONITORING 22
20 ETHICAL CONDUCT OF STUDY 22
20.1 INFORMED CONSENT 22
20.2 DATA RECORDING 23
20.3 COLLABORATING INVESTIGATORS 23
20.4 CONFIDENTIALITY 23
20.5 DATA PROTECTION 23
21 STUDY CONDUCT RESPONSIBILITIES 23
21.1 PROTOCOL AMENDMENTS 23
21.2 STUDY RECORD RETENTION 23
22 END OF STUDY 23
23 REPORTING, PUBLICATIONS AND NOTIFICATION OF RESULTS 24
23.1 EXPECTED VALUE OF THE RESULTS 24
24 PEER REVIEW 24

Treatment in Morning Versus Evening (TIME)

Eudra CT number 2011-001968-21

Approval Signature

By signing this document I am confirming that I have read, understood and approve the protocol for the above study.

	Signature	Date
Chief Investigator<br><br>Prof Tom MacDonald		3 March 2021

List of abbreviations

Blood Pressure	BP
Monitorización Ambulatoria para Predicción de Eventos Cardiovasculares	MAPEC
Ambulatory Blood Pressure Monitor	ABPM
Febuxostat vs Allopurinol Streamlined Trial	FAST
General Practitioner	GP
National Health Service	NHS
Prospective, Randomised, Open, Blinded-endpoint	PROBE
Portable Document Format	PDF
The Standard care versus Celecoxib Outcome Trial	SCOT

1 Summary

An outcome event trial of treated hypertensive subjects of all ages comparing evening dosing of usual antihypertensive therapy with conventional morning dosing is proposed. 20,000 subjects already taking antihypertensive medication in usual care will be recruited from general practices, secondary care settings, pharmacies, consented patient databases or by social media and followed up for 4 years. Subjects participate by registering on a study-specific website. Subjects who meet the inclusion criteria are randomised to continue taking medication at their usual time (usually morning) or to switch to taking medication in the evening. Subjects receive regular emails with simple links to record responses to track progress. Participants or surrogates can record adverse events or endpoints at any time on-line. This largely automated system has worked well in the pilot phase with good patient retention. Record-linkage to hospitalisations and deaths will be carried out and events adjudicated. The primary outcome is the composite endpoint of non-fatal myocardial infarction, non-fatal stroke or vascular death. A pilot study that has randomised 402 patients with > 1 year follow-up, has confirmed the feasibility of this approach.

This trial completed a successful pilot phase and the roll-out is funded by the British Heart Foundation.

2 Introduction

2.1 Background

Nocturnal blood pressure (BP) has consistently been a better predictor of cardiovascular outcome than daytime BP. As the day: night BP ratio increases, cardiovascular risk appears to decrease[1–4]. For example, in the Ohasama Study there was a linear relationship between the nocturnal decline in blood pressure and reduced cardiovascular mortality[5].

There is also evidence to suggest that antihypertensive drugs taken in the evening rather than in the morning reduce nocturnal blood pressure to a greater extent[6–8] and might have more benefit in hypertension alone[9,10], hypertension with renal disease[11,12], and hypertension with diabetes[13]. A recent study that examined the effect on nocturnal blood pressure of drugs taken in the evening in subjects with resistant hypertension found that bedtime dosing showed significantly lower 24-h means of systolic and diastolic BP (by 4.1/1.5 mm Hg) and that this difference between groups was driven by asleep BP (9.7/4.4 mm Hg lower).[14] Nocturnal dosing and subsequent lower nocturnal blood pressure might also explain the unexpectedly large benefit of ramipril in the Heart Outcomes Prevention Evaluation (HOPE) study[15,16]. The most recent and most compelling evidence in favour of nocturnal dosing comes from the Monitorización Ambulatoria para Predicción de Eventos Cardiovasculares (MAPEC) study which randomly assigned 2156 hypertensive patients to take all of their antihypertensive drugs on awakening or to take one or more of them at bedtime[17,18]. All patients in this study underwent ambulatory monitoring at baseline and yearly and all wore wrist activity meters to control for the effects of activity on BP and to determine when subjects were asleep[19]. Patients were followed up for 5.6 years. The study found that those who took one or more antihypertensive drugs at bed-time had 68 cardiovascular events whereas those who took all medication on awakening had 187 events a relative risk reduction of 64% ($p<0.001$). 48 hour ABPM showed lower systolic BP (122.1mmHg morning dosing v 120.8mmHg evening dosing $p=0.029$) with evening dosing and this difference was due to lower asleep systolic blood pressure (116.1mmHg morning dosing v 110.9mmHg evening dosing, $p< 0.001$). The MAPEC

study had several major limitations in that it was not prospectively powered, the process of randomisation was not reported, the end points included some unusual cardiovascular events and the endpoints were not adjudicated. However, even if more modest benefits than those in MAPEC can be confirmed then this would be a very major advance in the management of hypertensive patients and would be extremely cost-effective.

Calls for a large prospective randomised trial to confirm or refute the findings of the MAPEC study have been made[20]. The present study answers this call and seeks to discover if nocturnal dosing of antihypertensive medication reduces cardiovascular events compared with conventional morning dosing.

2.2 Scientific principles

The TIME study builds on successful novel methodology to track patient outcome using information technology (IT) and record-linkage to determine hospitalisations and deaths[21,22]. The entire project will be conducted on a secure web portal with patients signing up on-line and being followed up by email and record-linkage to national databases with end-point adjudication blinded to dosing time.

2.3 Hypothesis

The present study tests the hypothesis that nocturnal dosing of antihypertensive medication reduces cardiovascular events compared with conventional morning dosing.

2.4 Questions to be addressed

The question to be definitively answered is whether nocturnal dosing of antihypertensive medication is better than morning dosing for reducing cardiovascular events. Secondary questions examine whether there are any downsides to nocturnal dosing. Will patients accept nocturnal dosing? Clearly nocturnal diuretic use is likely to be an issue and data on this and how it can be managed is being collected (patients attempt nocturnal diuretic dosing, if not tolerated they try 6pm and finally revert to morning dosing). Nocturnal hypotension and its consequences (falls, fractures) are also being addressed. A sub-study also collects data on home blood pressure control.

Five sub-studies will be conducted assessing the following in relation to dosing time:

- Cognitive Function
- Sleep Quality
- Mood
- Genetics (potential)
- Chronotype

2.5 Pilot study

A rolling pilot phase (funded by the British Hypertension Society) randomised 402 patients. This was done by asking 3 primary care practices to send a letter of invitation to all of their treated hypertensive patients. In addition, posters advertising the TIME study were sent to all UK practices (n=15,158) to be displayed in their waiting room. In addition a YouTube video

and social media campaign was launched and an email sent to all staff in the Dundee Medical School.

The pilot phase has determined that about one in 26 treated hypertensive patients in each practice took up the invitation and were randomised after being invited by their GP. Only 5.6% of practices displayed the TIME study poster in their practice waiting room and the impact on recruitment was modest and posters did not stay up for long. The impact of the social media campaign is still being evaluated.

Importantly the pilot study showed that:

- The mean age of those randomising was 61 (median age 63) years.
- The cardiovascular risk profile of those who signed up to TIME pilot (based on the ASSIGN risk score [23]) showed a 10 year risk of 18% averaged over all age groups
- Patient retention was good. Over a one-year period only 14 subjects contacted us and withdrew from the randomised time of dosing but 11 of these agreed to email and record-linkage follow-up and the remainder agreed to record-linkage follow up. A further 57 subjects did not respond to email contact at some point in the first year but of these a patient nominated surrogate answered for 15 subjects. At least one subject had died and there were 6 reported CVAs and 4 MIs. However, no-one withdrew active consent for follow up.
- 207 pilot participants who reported owning a home BP monitor were asked to provide home BP measurements a week prior to randomisation and then regularly throughout the study. This provided high quality data. Measurements provided by study participants using their own home BP monitor will demonstrate the effect of am v pm dosing and may help validate adherence to nocturnal dosing especially in those taking Ramipril.

3 Study Objectives

3.1 Primary objective

A study to determine if antihypertensive therapy taken in the evening has improved cardiovascular outcomes compared with more conventional morning dosing.

3.2 Methods

TIME is a prospective, randomised, open-label, blinded end-point (PROBE design[24]) controlled clinical trial. It utilises IT to improve efficiency (www.timestudy.co.uk) Subjects sign up online and are followed-up by email. Endpoints are detected by record-linkage to hospitalisation and death certification records and by regular email contacts with patients, nominated surrogates and primary or secondary care physicians (all patients consent to their medical records being accessed). Original medical records will be retrieved from hospitalisation events to validate endpoints.

Study design

Schematic Diagram of Study

Treatment in the Morning versus Evening (TIME)

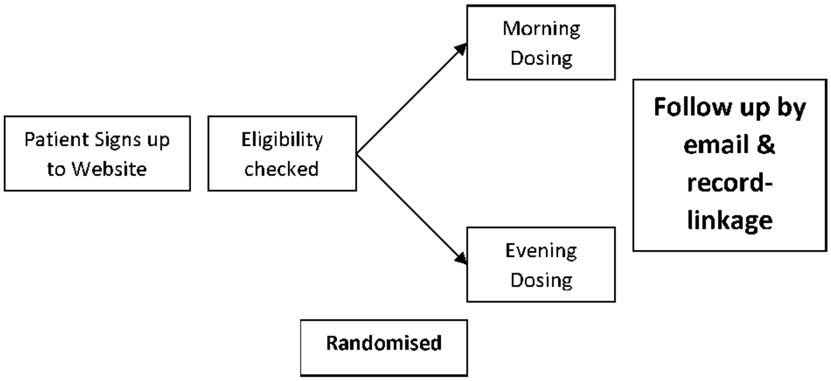

3.4 Study population

Hypertensive patients in the UK prescribed one or more once daily antihypertensive drug therapies.

4 Inclusion criteria

- Both diagnosed and treated for hypertension (all forms) with at least one antihypertensive drug.
- Age $\geq$ 18 years.
- Have a valid email address.

4.1 Exclusion criteria

- Subjects who take twice daily antihypertensive therapy.
- Subjects who work shift patterns that include a night shift.
- Subjects who are unwilling to consent to:
  - follow-up
  - provide a surrogate to be contacted and/or
  - give consent for their family practice to release follow up clinical data
  - to have their physical case records abstracted if required
  - to have their electronic case records searched and abstracted if required
  - to allow their consent form to be copied to authorities from whom the study team is requesting medical data.
- Those participating in a clinical trial of an investigational medicinal product (CTIMP) or who have done in the last 3 months.

5 Outcome measures

5.1 Primary endpoint

The primary endpoint is hospitalisation for the composite endpoint of non-fatal MI, non-fatal stroke or vascular death. This end-point is an appropriate composite end point in studies of hypertension[25].

5.2 Secondary endpoints

Secondary endpoints (in rank order) will include:

- Each component of the primary endpoint:
- Hospitalisation for non-fatal stroke
- Hospitalisation for non-fatal MI
- Vascular death
- All-cause mortality
- Hospitalisation or death from congestive heart failure.

Adherence to the evening dosing regimen v morning (patient reported) with particular reference to patients taking diuretic therapy.

Patient reported and hospitalised adverse events in the morning versus evening groups will be compared. In particular falls and fractures will be recorded.

Home BP readings taken by a subset of patients will be compared between morning & evening dosing.

5.3 End point adjudication

A medical records/administrative officer at Ninewells Hospital will coordinate the retrieval of hospital case records from all hospitals where record-linkage suggests a possible end-point has occurred to facilitate adjudication. The adjudication process works well in other studies currently run by our group. These data will be held securely.

Endpoint adjudication will be done by an endpoint committee. Data will be abstracted from any available source to allow the committee to perform adjudication. Hospitalisation data will be retrieved in collaboration with the medical records department at Ninewells Hospital. We will supply copies of the patient consent forms to facilitate this process. The records will be converted to PDF files and uploaded to our secure server. From there the records will be processed and suitably redacted to produce anonymised end-point packages for the end-point committee.

An endpoint committee charter will be developed as a separate document. This committee will have due regard of the published consensus diagnostic criteria for myocardial infarction[26], stroke[27], vascular death, and heart failure[28].
The endpoint committee terms of reference and their names and contact details will be detailed in the trial operations manual.

6 Participation selection and enrolment

6.1 Recruitment Strategy

Primary care
Collaborating general practitioners will bring this trial to the attention of all treated hypertensive patients in their practices. This will be done by displaying a Study poster or mailing a letter of invitation to all treated hypertensive patients in the practice (see operations manual for this letter and for the patient information sheet). These subjects may be identified from various sources including practice registers, prescribing records, searches of practice computer systems etc. Practitioners may also hand a letter to subjects attending for review of antihypertensive medication treatment.

With PCRN support in the UK, all treated hypertensive patients in research network practices will receive a letter of invitation. This is likely to be the dominant route of recruitment. Since most practices have a hypertension register this is likely to be a relatively simple search strategy. (Note that we spent some considerable time investigating the use of the Clinical Practice Research Data link system (www.cprd.com) as an aid to recruitment but ultimately abandoned this as it was more expensive and had no obvious advantages).

Secondary care
Secondary care clinics will send letters of invitation to previous clinic attendees. Posters will also be displayed in appropriate clinic areas.

Pharmacies
Pharmacists will bring this trial to the attention of all treated hypertensive patients in their databases either by information about the study being packaged with their prescription or by a letter of invitation.

Use of Consented Patient Databases
Databases of consented patients including, but not limited to, resources such as UK Biobank, Tayside Bioresource and Share will be utilised to contact patients to inform them of the study. Patients in these databases have consented to be contacted about further studies. Patients will be contacted by the database owners who will send approved letters of invitation.

Advertisements
Advertisements for the study will be placed in primary care, secondary care and where possible in other healthcare premises (pharmacies, etc). Articles and advertisements in the press may also be placed. (See the operations manual for examples of advertisements.) Advertisements, information about the study and links to the study website will also be placed on appropriate websites – for example the British Heart Foundation and SHARE. Social media platforms such as Twitter will also be used to distribute an advertisement for the study.

Estimate of consent rates
About 1 in 26 eligible subjects invited to participate in TIME actually randomised in the pilot study. There is some uncertainty about this number as it was based on a search of only 3 practices and for this reason we suggest writing 580,000 letters to randomise 20,000 subjects

or more as required to reach the number of endpoints needed. We will monitor the recruitment rate carefully.

6.2 Study timeline

Recruitment

All trial sign-up, consent and follow up is done via a secure web portal with email and telephone backup from the Dundee centre. TIME has been adopted by PCRNs and recruitment should move ahead swiftly. We expect that recruitment should be complete within 2 years of commencing the full study. TIME is technically a single centre study and this will make the process of regional approvals easier.

Participation in the study

To participate in the study patients must voluntarily log onto the study website. The study website will contain details of the study and the patient information sheet. If having read these the patient wishes to participate they will enter a valid email address and password to start the process of enrolment. An email with a validation web-link will be sent to the patient. Clicking on this will validate the patient's email address.

Follow up

At the end of the recruitment year there will be an average of 6 months follow up in the study. The study will continue with regular checks regarding the number of primary endpoints that have been generated. Once the estimated number of primary endpoints exceeds that of the number required to achieve statistical power the Steering Committee will decide on a date for participant involvement to end. Thereafter the trial will close down in an orderly fashion. A final record-linkage will be done 3 months after the end of the trial in order to capture late recorded events and the remaining time will be spent finalizing adjudications, locking the database and carrying out the analysis. We have two large studies running that rely on record-linkage for the outcome (SCOT and FAST) and these work extremely well. See: www.dundee.ac.uk/memo for details of these.

Around two months after we have ended the regular follow-up questionnaires and participants are no longer assigned a time to take their anti-hypertensive medications we will send out a further questionnaire to ask if they have changed the time they take their medication since the end of the study and their reasons for any change in time.

In addition to routine follow-up emails, an annual newsletter will be sent to participants.
Also through the Study website we wish to send Annual Newsletters informing participants of our latest news, and thanking them for their continued support. In our first newsletter we would like to reiterate the importance of complying with randomised medication dosing time and allowing us to follow their health through to the conclusion of the study.

6.3 Consenting participants

Patients will then be asked to enter some personal details in order to fill in an electronic consent form. The consent form (see operations manual) will contain check boxes to attest to consent for each aspect of the trial. A final check box will be marked *'Checking this box is equivalent to your signature in electronic form. Checking this box means that you agree to participate in*

the TIME Study'. Subjects will be sent a PDF of their completed consent form (note: the consent form will record the IP address of the person completing the form along with their email address and a date and time stamp.)

Screening for eligibility

Note that if subjects do not agree to certain aspects on the consent form then they will be ineligible to join the study.

Participants who have responded to an invitation to join the study by entering a valid email address and signing the consent form will then be invited to fill in forms to assess their eligibility and baseline characteristics.

Subjects who are < 18 years of age will be ineligible.

Subjects will be asked:

- Do you take tablets or capsules for the treatment of your blood pressure in both the morning and the evening?
- Do you habitually work a shift pattern that requires you to work night shifts?
- Are you participating in another clinical trial of investigational medicinal product (CTIMP) or have you done so in the last 3 months?

If subjects answer yes to any of these questions then they will be judged ineligible to participate in the study. However, subjects who have recently completed another study can join the TIME study once 3 months has elapsed after the end of the previous study. They will be able to do this using the same email address and password as previously.

6.5 Unintended Co-Enrolment

If co-enrolment occurs unintentionally, the person becoming aware of this should report it to both study teams and to the Sponsor. A decision should be made about continuation of or withdrawal from the study(s). The process followed and decision made should be clearly documented in the Study Files.

7 Randomisation

7.1 Computer randomisation

Randomisation will be done centrally using a computerised randomisation algorithm. Randomised status will be visible after enrolment, and randomisation will be confirmed by email.

7.2 Treatment allocation

Subjects will be randomised to take their antihypertensive medications in the morning (range 6am – 10am) or in the evening (range 8pm-midnight).

©University of Dundee TIME Study

8 Diuretic treatment: patient instructions

Many subjects may be taking thiazide or thiazide-like diuretics for the treatment of hypertension. A few subjects may be taking loop diuretics. These patients will receive further instructions.

8.1 Thiazide or thiazide-like diuretics

The following instructions will be given:

Diuretic tablets (sometimes known as 'water tablets' i.e. bendroflumethiazide) could theoretically increase urine volume at night and lead to early wakening to visit the toilet. In practice, when people have been on these tablets for a long time the effect on urine volume is small or absent. However, we recognise that this might still be a problem. For those people taking this sort of tablet we would like patients to try taking this tablet in the evening but if they find it a problem they could take it earlier in the evening (6pm). If this still does not resolve the problem then they should take it in the morning. We would like patients to tell us about this as we need to know if this really is an issue or not.

8.2 Diuretic therapy (with loop-type diuretics) and evening dosing

A few patients will be taking so-called loop diuretics like frusemide or bumetanide or similar. These tablets typically cause a prompt increase in urine volume that lasts a few hours. Patients taking these tablets will be aware of how long this effect lasts in them. We would like patients taking these drugs who are randomly allocated evening dosing to take these tablets at 6pm in the first instance. If this remains a problem then they should take it in the morning. We would like patients to tell us about this.

9 Withdrawal

Subjects will be free to withdraw from:

- Taking medication according to the time of randomisation.
- Receiving further follow up emails.
- From follow-up of their outcome by record-linkage or from contacting their surrogate or from contacting their family doctor.

Part of the objective of this study is to determine if subjects can continue to take medication according to the time of randomisation so we expect that a proportion of subjects will withdraw from taking their evening dose. We want to know the scale of withdrawal and why subjects withdrew and we will ask them to check one of the following reasons:

Subjects will be asked: Are you still taking your tablets in the morning / in the evening according to your allocation in this study?

If subjects say no, they will be asked to check all radio boxes that apply:

- I found taking tablets in the morning/evening inconvenient.
- I found that taking tablets in the morning/evening resulted in problems: Check all that apply:
    - Dizziness/light-headedness
    - Falls
    - Excessive visits to the toilet during the day/during the night

  - Sleep problems
  - Upset stomach/indigestion
  - Diarrhoea
  - Feeling generally less well
  - Muscle aches
  - Others (specify)
- I found that I forgot to take my tablets in the morning/evening.
- I found my blood pressure to be less well controlled taking tablets in the morning/evening
- I take a lot of tablets for other conditions in the morning/evening and I found it difficult to take my blood pressure tablets at other times.
- I got bored/fed up of taking tablets in the morning/evening.

They will then be asked:
- Are you still happy to receive emails from us to check on your progress?

Those that say no will get asked:
- Are you happy for us to track your outcome by checking your electronic and or paper medical records?

If they answer yes, we will ask:
- Do you wish to be informed about the results of this study when they are available?

Whether yes or no they will then get a message:
Thank you for participating in TIME

10 Medicinal products
There are no study drugs in this study. This is a study to determine the optimal time to take usual prescribed antihypertensive medication.

10.1 Participant compliance
Participants will receive emails after 1 month and then every three months asking them if they are still taking their treatment at the time they were allocated at randomisation to determine compliance with randomised time of dosing.

10.2 Other medications
Details of participant's current regular medications will be collected at baseline and at 3 monthly intervals throughout the study.

11 Data collection
This study will capture data directly from patients or their nominated surrogates. In addition, record-linkage to hospitalisations and deaths will be used where available. In addition, we will gather specific consent from patients to have access to their health care records whether electronic diagnostic or death data (HES data & ONS data in England (the Connecting for Health Research Capability Program has agreed in principle to facilitate this) ISD & GRO data in Scotland), their acute care summary (if this exists), their paper hospital or electronic records

(including laboratory and x ray findings) and their paper or electronic primary care records. Medical records staff at Ninewells Hospital will coordinate the retrieval of these data from hospital case records. Further data may be retrieved from general practitioner records. Such records may be converted into portable document format (PDF) for the purposes of electronic storage within the study database. These will be redacted for subject details and time of dosing before being sent to the endpoint committee.

Data will be validated at the point of entry into the patient completed on-line e-Clinical Report Form and at regular intervals during the study. Data discrepancies will be flagged to the study coordinators and any data changes will be recorded in order to maintain a complete audit trail (reason for change, date change made, who made change).

11.1 Data storage

These data will be held in a secure database in a secure environment within the sole control of the Medicines Monitoring Unit at Ninewells Hospital & Medical School.

11.2 Data retention

To enable evaluations and/or audits, the investigators will keep records, including the identity of all participating patients (sufficient information to link records, all original informed consent data, adverse event data and any source documents). The records will be securely retained and archived by the study sponsor according to ICH and local regulations.

11.3 Data access

Participating subjects will be able to have sight of their own data on request and will be allowed to comment on perceived inaccuracies therein.

12 Statistics and data analysis

The primary outcome will be a comparison of time to first event comparing morning versus evening dosing using an intention to treat analysis.

12.3 Bias

The current trial requires that subjects have a valid email address and that they are computer literate enough to sign up to the study on-line. A previous study done by our group trialled email, text and phone contact and email was by far the most successful[Error! Bookmark not defined.]. This IT-literate requirement may bias the study population. However, the mean age of the subjects recruited into the pilot study was 61 years which appears reasonably representative of the hypertensive population.

Potential risks & hazards

One reason for carrying out this study is to determine whether subjects can tolerate taking medication in the evening. This is being prospectively measured. In addition, subjects are asked to formally report adverse events associated with dosing time and whether nocturnal diuretic dosing is feasible. In particular falls and fractures are being captured as a surrogate for nocturnal hypotensive events during toileting. Finally, the cognitive function sub-study is designed to monitor any effects of nocturnal dosing on cognitive function.

Sample size

Proposed sample size: Evidence of feasibility & power calculation
For a 2 sided test to detect 20% superiority (a much more modest benefit than that shown in the MAPEC study) at 80% power, 631 events are needed. Based on the profile of subjects recruited into the pilot phase of the study, a trial with a four year follow up period would need to randomise 19,740 subjects. Because the primary analysis is intention to treat and because few subjects are likely to withdraw consent for record-linkage follow up, only relatively minor (< 5%) inflation of the 19,740 subjects is required to compensate. So a target of 20,000 subjects will be randomised.

Primary care population
80% of the UK population is aged 16 or older and 30% of these are hypertensive according to the Health Survey for England. An average practice of 6,000 patients will contain 720 treated hypertensive patients. To be conservative we have restricted this to 500 subjects per practice. If 1 in 26 subjects randomise into TIME then an average practice will randomise 20 subjects. As recruitment will be from primary, secondary and via consented patient databases we estimate that about 500 UK practices are required. The chief investigator is currently running two large trials (SCOT & FAST) with a network of >700 practices so the recruitment of patients seems feasible.

Compliance and loss to follow up
The pilot phase of this trial has determined that 14 out of the 388 randomised subjects withdrew from randomised treatment over the first year of follow up. However, all subjects gave permission to being followed up by record-linkage and so remain in the primary analysis. Some patients have been lost to email follow up but since record-linkage to hospitalisations and deaths will be used to determine the primary outcome of this study, the number lost is likely to be smaller. With regard to participants who have been lost to email follow up, there could be several possible reasons for this, for example: a change of email address, no longer having access to a computer or no longer wishing to participate in the TIME study. We shall send a letter from MEMO to those identified to enable us to update our records. If they do not respond to a letter we will make contact with their General Practitioner.

12.4 Planned subgroup analyses
Pre-specified subgroup analyses will examine gender, age and prior cardiovascular disease.

13 Adverse Events
The present study will collect only adverse events associated with changing the time of dosing. This will consist of the following questions:

As a result of the time you take your blood pressure medication have you experienced any increase or onset of the following conditions:

- Dizziness/light-headedness
- Falls
- Excessive visits to the toilet during the day/during the night
- Sleep problems
- Upset stomach/indigestion

©University of Dundee TIME Study

- Diarrhoea
- Feeling generally less well
- Muscle aches
- Others (specify)

No medication related adverse events will be recorded.
Participants will report adverse events related to the time of dosing at any time after consenting to join the trial. All time of dosing related reported adverse events (AEs) that occur after joining the trial will be recorded in the CRF. In the case of an AE, patients will judge whether they are happy to continue taking treatment at the time randomised or whether they wish to change or revert to the alternative time of dosing or whether they wish to discontinue the study.

13.1 Early stopping

If recruitment exceeds expectations or the event rate is higher than expected or the IDMC advise this then the trial may stop early.

14 Competing Studies

We are not aware of any competing studies of hypertension that would conflict with the TIME study.

15 Trial management and oversight arrangements

15.1 Sponsorship

The trial is sponsored by the University of Dundee / NHS Tayside (TASC).

The study team will have the following roles:

The Chief Investigator and Principal Investigator will drive the project and along with the part time research fellow will answer all patient questions. Our unit currently runs two large outcome studies one with 7,300 randomised and another with a target of 5,700 so the unit is well acquainted with running large outcome trials. A part time research manager will drive logistic issues such as regional approvals and event adjudication supported by the part time administrator/secretary. A full time Information Technology / programmer will be the lynchpin of the study e-Clinical Record Forms & maintaining and backing up the web portal. A medical records clerk will retrieve original case records from hospitals to enable event adjudication. Finally a small amount of statistician time will be based at the Robertson Centre in Glasgow in order to service the Independent Data Monitoring Committee.

15.2 Trial executive committee

A trial executive committee will be constituted to guide the day to day running of the study.

15.3 Ownership of study database

The study database will be generated and stored at the Medicines Monitoring Unit, University of Dundee. Database ownership will be jointly shared by participating BHS research principal investigators at participating centres.

15.4 Potential sub-studies

Several sub-studies have been proposed. All sub-studies will be approved by the trial steering committee.

16 Sub-studies

16.1 Cognitive function sub-study

Cognitive impairment is prevalent in people with hypertension, particularly at age ≥70 years[29,30]BP-lowering has relatively little impact on the rate of cognitive decline in previous trials, but most trials used morning dosing only. It is possible that evening-dosing might reduce the risk of chronic cognitive decline as well as the risk of acute vascular events by controlling nocturnal hypertension and reducing afternoon hypotension.

The main purpose of cognitive testing in TIME will be to detect temporal change, particularly any sustained reduction in performance. We therefore propose to use combined telephone testing with the T-MOCA and the TICS to assess cognitive function in consenting participants in the TIME trial. Our previous experience shows that testing takes between 15-30 minutes and that it is well tolerated by patients.

16.2 Sleep sub-study

All patients signing up to the TIME study will be invited to take part in a sub-study investigating sleep quality. Participants will be asked to complete an online questionnaire designed to assess sleep quality at study entry and then repeated at 3 months and annually throughout the duration of TIME. The main purpose of the sleep quality assessment is to assess whether dosing time of antihypertensive medication has significant positive or negative effects on sleep quality.

16.3 Mood sub-study

All study participants will be invited to take part in a sub-study exploring the links between mood disorder, hypertension and cardiovascular disease. Participants will be asked to complete a single online questionnaire to assess their current mood status and history of mood disorder.

16.4 Genetics sub-study (potential)

All subjects will be asked if they would consent to participate in a genetics sub-study where their DNA will be bio-banked for possible future genetic studies. Subjects who consent will be asked to provide a cheek swab or saliva collection by post or they may be asked to join the GO-SHARE project.

16.5 Chronotype Sub-study

All patients signed up to the TIME study will be asked to complete the ultra-short version of the Munich ChronoType Questionnaire (μMCTQ) [31] and the General Practice Physical Activity Questionnaire (GPPAQ). [32] We will use information from the μMCTQ to determine their chronotype and, also, to evaluate the social jetlag state, the mismatch between the chronotype and the external environment. Then, we will combine these data with the typical grade, frequency and temporal distribution of physical activity over 24 hours, to investigate association between chronotype, social jetlag and physical activity. The main aim of this substudy will be to determine if patients take their medication in line with their chronotype and if this has an impact on adherence to treatment, adverse events and outcomes. In addition, we will evaluate whether specific outcomes may be linked to social jetlag state. We will use this data to determine if there is any benefit to taking medication at a time linked to chronotype, and whether physical activity can mitigate increased health risks associated with social jetlag.

©University of Dundee TIME Study

17 Trial management

A Trial Manager will oversee the study and will be accountable to the Chief Investigator.

17.1 Central trial office

The Central Trial Office will provide support to each site.

17.2 Trial steering committee

A Trial Steering Committee (TSC) will be established to oversee the conduct and progress of the trial. This will consist of principal investigators in each of the participating trial centres. All publications will be approved by the steering committee.

18 Independent data monitoring committee

An Independent Data Monitoring Committee (IDMC) will be established to oversee the safety of subjects in the main trial. A charter for the IDMC will be detailed in the trial operations manual.

19 Inspection of records

Principal Investigators and institutions involved in the study will permit trial related monitoring, audits or REC review. In the event of an audit, the Investigator agrees to allow the Sponsor, representatives of the Sponsor or regulatory authorities direct access to all study records and source documentation.

19.1 Study monitoring

Since this study intervention is time of dosing and there are no investigational medical products, no formal study monitoring is proposed. However, the sponsor is free to audit this study if they see fit.

20 Ethical conduct of study

A favourable ethical opinion was obtained from the appropriate REC prior to commencement of the pilot study. A further favourable opinion has been obtained to roll out this study.

20.1 Informed consent

The Investigator is responsible for ensuring informed consent is obtained before any protocol specific procedures are carried out. The decision of a participant to participate in clinical research is voluntary and will be based on a clear understanding of what is involved.
Participants will receive adequate written information – appropriate Participant Information and Informed Consent Forms will be provided.

Participant will be given opportunities to clarify any points they do not understand and, ask for more by using a 'Contact Us' link on the study website. Participants are free to take as much time as they require to consider the information provided. Participants may withdraw their consent to participate at any time.

Participant will be informed and will agree to their medical records being inspected by study staff but understand that their personal details will not be disclosed.

Participants will be emailed a PDF of their electronically signed consent form. Consent forms will be stored electronically by the investigator.

A separate patient information sheet and consent form will be provided for those who indicate their willingness to provide a mouth swab genetics sample.

20.2 Data recording

The study subject is responsible for the quality of the data recorded in the CRF but electronic checks and possible augmentation by study staff will be done.

20.3 Collaborating investigators

Collaborating investigators will be responsible for dealing with the local issues of bringing the trial to the attention of possible subjects either in clinics or in primary care. Since all patients by their own volition decide to go to the study website and sign up, the usual investigator / study subject relationship is not present.

20.4 Confidentiality

All records will be kept in a secure storage area with limited access. Clinical information will not be released without the written permission of the participant, except as necessary for auditing by the Sponsor, its designee, Regulatory Authorities, or the REC.

20.5 Data protection

The study will comply with the requirements of the Data Protection Act 1998 with regard to the collection, storage, processing and disclosure of personal information and will uphold the Act's core principles. Access to collated participant data will be restricted to appropriate study staff.

Computers used to collate the data will have limited access measures via user names and passwords.

Published results will not contain any personal data that could allow identification of individual participants.

21 Study conduct responsibilities

21.1 Protocol amendments

Changes in research activity, except those necessary to remove an apparent, immediate hazard to the participant, will be reviewed and approved by the Chief Investigator and Sponsor. Amendments to the protocol will be submitted in writing to the appropriate REC, Regulatory Authority and local R&D for approval prior to participants being enrolled into an amended protocol.

21.2 Study record retention

All study documentation will be kept for at least 5 years.

22 End of study

The end of study will be announced when sufficient events have accrued to allow the study to be analysed except where the study steering committee advises otherwise.

The chief investigators and/or the trial steering committee have the right at any time to terminate the study for clinical or administrative reasons.

The end of the study will be reported to the REC and Regulatory Authority within 90 days, or 15 days if the study is terminated prematurely. The Investigators will inform participants and ensure that the appropriate follow up is arranged for all involved.

A summary report of the study will be provided to the REC and Regulatory Authority and all participants who request this within 1 year of the end of the study.

23 Reporting, publications and notification of results

The steering committee will publish the study protocol (where possible) and will undertake to make public the study results. The major form of publication will be articles in scientific journals but presentations and press releases may also be made. All subjects who request the study results will be emailed a copy of papers accepted for publication.

23.1 Expected Value of the Results

If the TIME study shows definite benefits of dosing antihypertensive medication in the evening rather than the morning then this would represent the most cost-effective advance in the treatment of hypertension and the prevention of CV disease in recent years. Balanced against this, TIME will determine whether subjects can tolerate nocturnal dosing and whether there are any adverse effects of nocturnal versus morning dosing.

24 Peer review

Members of the British Hypertension Society research network have peer reviewed the study protocol. In addition the study was separately peer reviewed by referees for the British Heart Foundation.

25 References

1. Fagard RH, Celis H, Thijs L, et al. Daytime and nighttime blood pressure as predictors of death and cause-specific cardiovascular events in hypertension. *Hypertension.* 2008;51:55-61. doi:10.1161/HYPERTENSIONAHA.107.100727.
2. Fagard RH, Thijs L, Staessen JA, Clement DL, De Buyzere ML, De Bacquer DA. Night–day blood pressure ratio and dipping pattern as predictors of death and cardiovascular events in hypertension. *J Hum Hypertens.* 2009;23:645. doi:10.1038/jhh.2009.9.
3. Dolan E, Stanton A, Thijs L, et al. Superiority of ambulatory over clinic blood pressure measurement in predicting mortality - The Dublin Outcome Study. *Hypertension.* 2005;46:156-161. doi:10.1161/01.HYP.0000170138.56903.7a.
4. Brotman DJ, Davidson MB, Boumitri M, Vidt DG. Impaired diurnal blood pressure variation and all-cause mortality. *Am J Hypertens.* 2008;21(1):92-97. doi:10.1038/ajh.2007.7.
5. Ohkubo T, Hozawa A, Yamaguchi J, et al. Prognostic significance of the nocturnal decline in blood pressure in individuals with and without high 24-h blood pressure: The ohasama study. *J Hypertens.* 2002;20(11):2183-2189. doi:10.1097/00004872-200211000-00017.
6. Hermida RC, Calvo C, Ayala DE, et al. P-148 MP-11: Treatment of non-dipper essential hypertension with bedtime administration of valsartan. *Am J Hypertens.* 2005;18(S4):61A - 61A. doi:10.1016/j.amjhyper.2005.03.166.
7. Hermida RC, Ayala DE. Chronotherapy with the angiotensin-converting enzyme inhibitor ramipril in essential hypertension: improved blood pressure control with bedtime dosing. *Hypertension.* 2009;54(1):40-46. doi:10.1161/HYPERTENSIONAHA.109.130203.

8. Kuroda T, Kario K, Hoshide S, et al. Effects of Bedtime vs. Morning Administration of the Long-Acting Lipophilic Angiotensin-Converting Enzyme Inhibitor Trandolapril on Morning Blood Pressure in Hypertensive Patients. *Hypertens Res*. 2004;27(1):15-20. doi:10.1291/hypres.27.15.
9. Hermida RC, Calvo C, Ayala DE, López JE. Decrease in urinary albumin excretion associated with the normalization of nocturnal blood pressure in hypertensive subjects. *Hypertension*. 2005;46(4):960-968. doi:10.1161/01.HYP.0000174616.36290.fa.
10. Hermida RC, Ayala DE, Calvo C. Administration-time-dependent effects of antihypertensive treatment on the circadian pattern of blood pressure. *Curr Opin Nephrol Hypertens*. 2005;14(5):453-459. https://www.ncbi.nlm.nih.gov/pubmed/16046904.
11. Hermida RC, Ayala DE, Smolensky MH, et al. Chronotherapy improves blood pressure control and reduces vascular risk in CKD. *Nat Rev Nephrol*. 2013;9(6):358-368. doi:10.1038/nrneph.2013.79.
12. Hermida RC, Ayala DE, Mojon A, Fernandez JR. Bedtime Dosing of Antihypertensive Medications Reduces Cardiovascular Risk in CKD. *J Am Soc Nephrol*. 2011;22(12):2313-2321. doi:10.1681/ASN.2011040361.
13. Hermida RC, Ayala DE, Mojón A, Fernández JR. Influence of Time of Day of Blood Pressure–Lowering Treatment on Cardiovascular Risk in Hypertensive Patients With Type 2 Diabetes. *Diabetes Care*. 2011;34(6):1270-1276. doi:10.2337/dc11-0297.
14. Hermida RC, Ayala DE, Mojón A, Fernández JR. Effects of time of antihypertensive treatment on ambulatory blood pressure and clinical characteristics of subjects with resistant hypertension. *Am J Hypertens*. 2010;23(4):432-439. doi:10.1038/ajh.2009.260.
15. Yusuf S, Sleight P, Pogue J ft, Bosch J, Davies R, Dagenais G. Effects of an angiotensin-converting-enzyme inhibitor, ramipril, on cardiovascular events in high-risk patients. *N Engl J Med*. 2000;342(3):145-153. https://europepmc.org/abstract/med/10639539.
16. Svensson P, de Faire U, Sleight P, Yusuf S, Ostergren J. Comparative effects of ramipril on ambulatory and office blood pressures: a HOPE Substudy. *Hypertension*. 2001;38:E28-E32.
17. Hermida RC. Ambulatory blood pressure monitoring in the prediction of cardiovascular events and effects of chronotherapy: rationale and design of the MAPEC study. *Chronobiol Int*. 2007;24(4):749-775. doi:10.1080/07420520701535837.
18. Hermida RC, Ayala DE, Mojón A, Fernández JR. Influence of circadian time of hypertension treatment on cardiovascular risk: results of the MAPEC study. *Chronobiol Int*. 2010;27(8):1629-1651. doi:10.3109/07420528.2010.510230.
19. Leary AC, Donnan PT, MacDonald TM, Murphy MB. Physical activity level is an independent predictor of the diurnal variation in blood pressure. *J Hypertens*. 2000;18(4):405-410. https://www.ncbi.nlm.nih.gov/pubmed/10779090.
20. Basile JN, Bloch MJ. Analysis of Recent Papers in Hypertension: Nighttime Administration of At Least One Antihypertensive Medication is Associated With Better Blood Pressure Control and Cardiovascular Outcomes in Patients With Type 2 Diabetes or Chronic Kidney Disease Departm. *J Clin Hypertens*. 2013;15(1):2-4. doi:10.1111/jch.12028.
21. Mackenzie IS, MacDonald TM, Shakir S, et al. Influenza H1N1 (swine flu) vaccination: a safety surveillance feasibility study using self-reporting of serious adverse events and pregnancy outcomes. *Br J Clin Pharmacol*. 2012;73:801-811. doi:10.1111/j.1365-2125.2011.04142.x.
22. Ford I, Murray H, Packard CJ, et al. Long-term follow-up of the West of Scotland Coronary Prevention Study. *N Engl J Med*. 2007;357(15):1477-1486. doi:10.1056/NEJMoa065994.
23. NHS Scotland. ASSIGN Score. NHS Scotland. www.assign-score.com. Published 2014. Accessed January 30, 2013.
24. Hansson L, Hedner T, Dahlof B. Prospective randomized open blinded end-point (PROBE) study. A novel design for intervention trials. Prospective Randomized Open Blinded End-Point. *Blood Press*. 1992;1:113-119. http://www.ncbi.nlm.nih.gov/pubmed/1366259.
25. Collaboration AT. Collaborative meta-analysis of randomised trials of antiplatelet therapy

for prevention of death, myocardial infarction, and stroke in high risk patients. *BMJ*. 2002;324(7329):71-86. https://www.bmj.com/content/bmj/suppl/2002/01/11/324.7329.71.DC2/ppr71.pdf.

26. Alpert JS, Thygesen K, Antman E, Bassand JP. Myocardial infarction redefined--a consensus document of The Joint European Society of Cardiology/American College of Cardiology Committee for the redefinition of myocardial infarction. *J Am Coll Cardiol*. 2000;36:959-969. http://www.ncbi.nlm.nih.gov/pubmed/10987628.
27. Adams Jr. HP, Adams RJ, Brott T, et al. Guidelines for the early management of patients with ischemic stroke: A scientific statement from the Stroke Council of the American Stroke Association. *Stroke*. 2003;34:1056-1083. doi:10.1161/01.STR.0000064841.47697.22.
28. Swedberg K, Cleland J, Dargie H, et al. Guidelines for the diagnosis and treatment of chronic heart failure: executive summary (update 2005): The Task Force for the Diagnosis and Treatment of Chronic Heart Failure of the European Society of Cardiology. *Eur Hear J*. 2005;26:1115-1140. doi:10.1093/eurheartj/ehi204.
29. Gorelick PB, Scuteri A, Black SE, et al. Vascular contributions to cognitive impairment and dementia: a statement for healthcare professionals from the american heart association/american stroke association. *Stroke*. 2011;42(9):2672-2713. doi:10.1161/STR.0b013e3182299496.
30. Pendlebury ST, Rothwell PM. Prevalence, incidence, and factors associated with pre-stroke and post-stroke dementia: a systematic review and meta-analysis. *Lancet Neurol*. 2009;8(11):1006-1018. doi:10.1016/S1474-4422(09)70236-4.
31. Ghotbi N., Pilz L.K., Winnebeck E., Vetter C., Zerbini G., Lenssen D., Frighetto G., Salamanca M., Costa R., Montagnese S., et al. The µMCTQ—An ultra-short version of the Munich ChronoType Questionnaire. in press. [Google Scholar]
32. https://www.gov.uk/government/publications/general-practice-physical-activity questionnaire-gppaq

25 TIME STUDY FLOW DIAGRAM

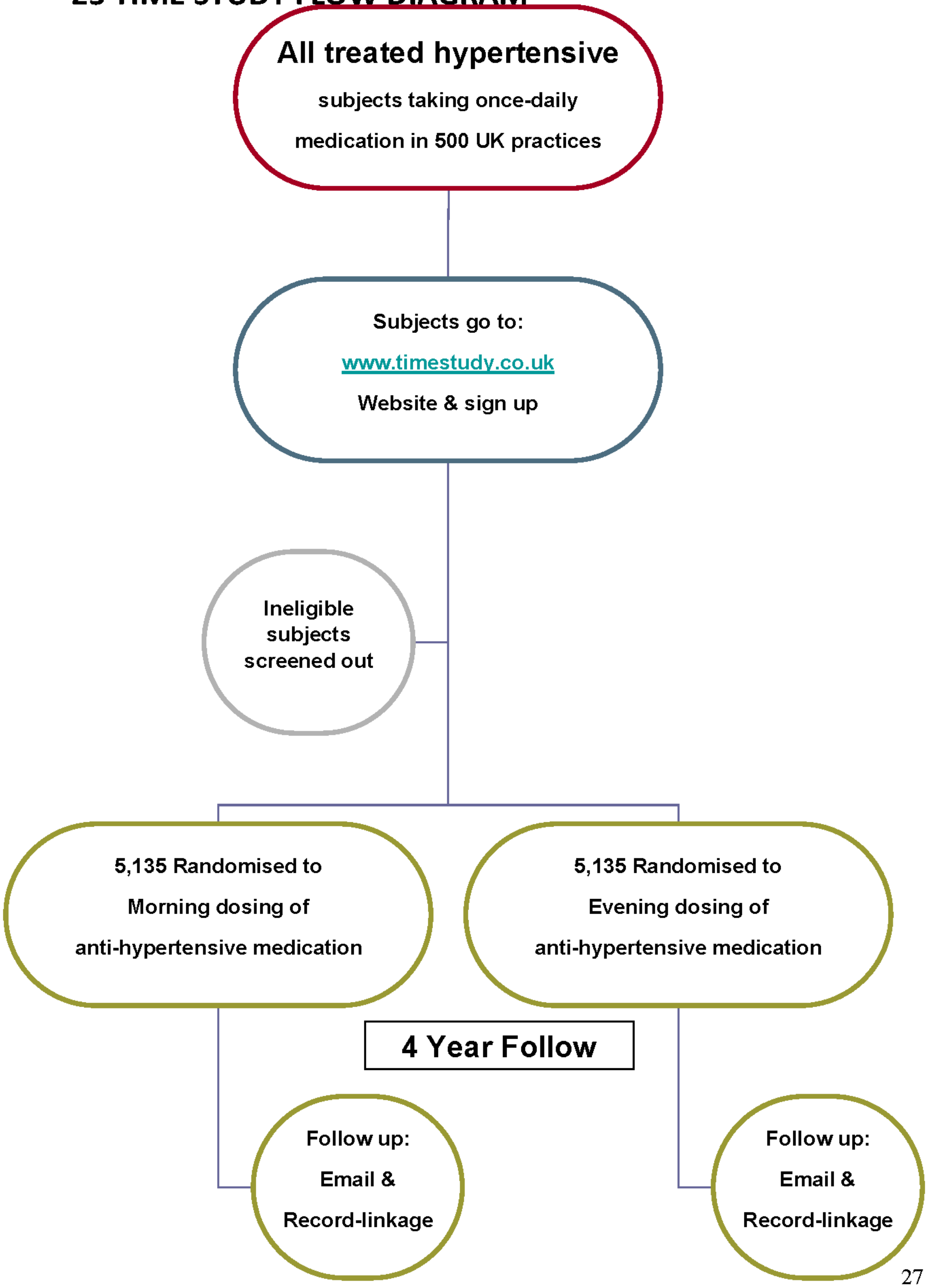

©University of Dundee TIME Study

Endpoint Committee Charter

Treatment In Morning Versus Evening Study (TIME)

Clinical Endpoint Committee Charter

Version:	1.5
Document Date	9th March 2022

Contents

1. Introduction 3
2 Composition and responsibilities of the CEC 3
2.1 CEC members and responsibilities 3
2.2 CEC coordination staff and responsibilities 4
3 Events to be reviewed 4
3.1 Deaths 4
3.2 Non-fatal events 5
4 Adjudication process 5
4.1 Identification and reporting of events 6
4.2 Study team review 6
4.3 Phase 1 CEC review 7
4.4 Incomplete event data 7
4.5 Phase 2 CEC review 7
4.6 Adjudication timelines 8
4.7 Clinical data to be provided 8
5 Clinical Event definitions 9
5.1 Hospitalisation 9
5.2 Non-fatal events 10
5.2.1 Acute myocardial infarction 10
5.2.2 marker positive acute coronary syndrome (ACS) 14
*5.2.3 Hospitalisation for troponin-negative cardiac chest pain** 15
5.2.4 Hospitalisation for other chest pain (not endpoint)* 15
5.2.5 Stroke 16
5.2.6 Hospitalisation for heart failure 17
5.3 Fatal events 18
5.3.1 Cardiovascular deaths 19
5.3.2 Non-cardiovascular deaths 21
5.3.3 Undetermined cause of death 21
6 Approvals 22

Appendix A. ICD and OPCS CODES 23

1. Introduction

The primary objective of the TIME (Treatment in the Morning versus Evening) study is to determine whether antihypertensive therapy taken in the evening has improved cardiovascular outcomes compared with more conventional morning dosing. The primary endpoint of the study is the composite endpoint of non-fatal myocardial infarction, non-fatal stroke or vascular death. Secondary outcomes include all cause mortality and hospitalisation or death from congestive heart failure. Prespecified cardiovascular and death events will be classified independently by the Clinical Endpoint Committee (CEC) utilising a consistent and unbiased classification system via an endpoint adjudication web portal. The CEC will be blinded regarding any information relating to the randomisation group.

2 Composition and responsibilities of the CEC

2.1 CEC members and responsibilities

The CEC consists of the following physicians specialising in cardiology and/or stroke:

CEC Member	Affiliation
Professor Chim C Lang (Chair)	University of Dundee and Ninewells Hospital, Dundee
Dr Priya Nair	Perth Royal Infirmary and Ninewells Hospital, Dundee
Dr Ify Mordi	University of Dundee and Ninewells Hospital, Dundee
Dr Jagdeep Singh (Left committee April 2022)	University of Dundee and Ninewells Hospital, Dundee
Dr Zaid Iskandar (joined committee 31/07/2018, left committee April 2022)	University of Dundee and Ninewells Hospital, Dundee
Dr Ben New (Joined Committee August 2021)	University of Dundee and Ninewells Hospital, Dundee
Dr Ali Kashan (Joined Committee May 2021)	University of Dundee and Ninewells Hospital, Dundee
Dr Qaiser Zeb (Joined Committee May 2021)	University of Dundee and Ninewells Hospital, Dundee
Dr Mon Myat Oo (Joined Committee May 2021)	University of Dundee and Ninewells Hospital, Dundee

In the event that a CEC member is unable to continue participation, the CEC chairman will recommend a replacement to the Sponsor. The Sponsor has the final decision as to the replacement. CEC members may not participate in the study as principal or co- investigators, nor should they routinely participate in the medical

care of a patient in the study, except in an emergency.

The CEC Chairman will
- Act as the primary liaison between the CEC and the Sponsor (University of Dundee)
- Act as the primary liaison between the CEC and the study team
- Be responsible for the overall conduct of the CEC
- Review other reported cardiovascular and non-cardiovascular events (i.e. those not appearing to constitute event types requiring CEC adjudication) to ensure that potential endpoint events that would require CEC adjudication have not been missed
- Communicating with the study team to resolve any data queries
- Liaising with the CEC members to arrange CEC meetings as necessary
- Ensuring that final event classification decisions reached as a result of CEC meetings are entered promptly into the endpoint adjudication web portal

CEC members will be responsible for:
- Participating in training on the adjudication process and trial-specific events and definitions
- Reviewing the relevant clinical data about a subject identified as having experienced a suspected event that requires adjudication
- Adjudicating pre-specified clinical events (see section 3) according to the definitions outlined in section 5 of this charter
- Timely submission of event adjudication decisions
- Communicating with the study team about needs when necessary.
- Attending scheduled CEC meetings or teleconferences throughout the study

2.2 CEC coordination staff and responsibilities
The CEC coordinating staff will be responsible for coordinating the day-to-day operations of the CEC. The CEC coordinating staff will assist the study team.

3 Events to be reviewed
The primary end point of the TIME study is improvement in cardiovascular outcomes. Therefore fatal and non-fatal endpoints will require adjudication.

3.1 Deaths
The CEC will review all reported deaths and classify the cause of death according to the following schema:
- Non-cardiovascular
  A definite non-cardiovascular cause of death must be identified
- Cardiovascular (CV)
  - Death due to acute myocardial infarction
  - Death due to stroke
  - Sudden cardiac death
  - Death due to heart failure
  - Cardiovascular procedure-related death
  - Other cardiovascular death (e.g. pulmonary embolism, ruptured aortic

aneurysm)

- Undetermined cause of death (i.e. cause of death unknown)

Note on the classification of haemorrhagic deaths
Deaths due to gastrointestinal haemorrhage (e.g. from a peptic ulcer) will be classified as non-cardiovascular deaths.
Deaths due to vascular disease leading to fatal haemorrhage (e.g. ruptured aortic aneurysm) will be classified as “other cardiovascular” deaths (see above).
Deaths due to vascular trauma leading to fatal haemorrhage (e.g. stabbing) will be categorized as death due to trauma (non-cardiovascular death).
Similarly, deaths due to other types of secondary haemorrhage will be ascribed to the primary aetiology (e.g warfarin overdose, coagulopathy, etc) (non-cardiovascular death).

3.2 Non-fatal events

The CEC will review the following reported non-fatal cardiovascular events:

- Acute myocardial infarction/biomarker positive acute coronary syndrome (reported to have been a reason for hospitalisation or to have occurred *during* a hospitalisation)
- Hospitalisation for angina*/other cardiac chest pain (e.g. troponin-negative)* (whether the angina/chest pain event was reported to have been the primary, or a contributing, reason for hospitalisation)
- Stroke/ possible stroke ** (whether reported to have been hospitalised or non- hospitalised or to have occurred *during* a hospitalisation)
- Hospitalisation for heart failure (whether the heart failure event was reported to have been the primary, or a contributing, reason for hospitalisation)

NB: Coronary/cerebral revascularisation procedures done over the course of the study will not be adjudicated by the CEC.
*Reported hospitalisation for angina/other cardiac chest pain events are secondary study endpoints and such events will be reviewed by the CEC to ensure that acute myocardial infarction/biomarker positive ACS events have not been missed.

**Brief documentation (eg discharge summary or clinic letter) relating to likely or definite TIAs, which are not thought by the study team to be stroke events, will be sent to the EPC to ensure that stroke events have not been missed. The EPC will not be asked to validate a diagnosis of TIA because of the difficulties (including the transience of the symptoms and the lack of a definitive test) involved in doing this reliably. TIA events will be reviewed simply as potential strokes and classified as ‘stroke’ or ‘not an endpoint. If additional information is required for adjudication of a likely stroke, reviewers can request this prior to making their decision.

NB All hospitalised TIA events that are identified (e.g. from hospitalisation codes or by study site investigators) will be collected by the Sponsor during the course of the study.

Other non-fatal cardiovascular events will not routinely be reviewed by the CEC. However, these events will be reviewed by the study team to ensure that potential

endpoint events which would require adjudication by the CEC are not missed. If the study team identifies a potential endpoint event, further information will be requested, as required and, if necessary, the event will be allocated to the CEC for adjudication.

4 Adjudication process

The CEC chair will be copied into the first 20 reported events for review. A stroke physician will be involved in the review of any possible stroke events. For the first 20 reported events requiring adjudication, the events will be reviewed at a CEC meeting or teleconference with all CEC members, and the CEC chair present. The purpose of this committee review will be to ensure that all committee members are aligned with regard to the application of the event definitions described in this charter.

4.1 Identification and reporting of events

All deaths and hospitalisations occurring within Scotland will be retrieved regularly from the General Register Office (GRO) database and the Scottish Morbidity Record One (SMR1) database, respectively. Hospitalisations and deaths occurring in England will also be retrieved regularly from centralised electronic records.

Potential endpoints will be identified when specific ICD and OPCS codes are detected electronically. Potential endpoints may also be identified from manual SAE reporting throughout the study.

Deaths and hospitalisations that occur outside the Scottish and English record-linkage framework will be identified by the study team. These events will be identified as potential endpoints if appropriate by the study team.

When potential endpoint events are identified, trained clinicians will scrutinise primary and secondary care records as well as death certification data, where appropriate. The data reviewed will be summarised onto *event pages* that have been specially designed to ensure that the information required for adjudication is captured. Where possible, the data will be supplemented by scanned images of relevant supportive source documentation (see section 4.7). Supportive source documents will be scrutinised to ensure that all data is blinded to both patient identity and randomisation before review by the CEC. The study team will review the *event packet* containing the relevant data for a reported event and check it for completeness. If required data is missing or incomplete, the process outlined in section 4.4 will be followed. The event data will be posted on the endpoint adjudication portal by the study team for review by the CEC members

4.2 Study team review

Event packets for those events that require review by the CEC (see sections 3.1 and 3.2) will be allocated on a regular basis to a pair of CEC cardiology members (which will
not include the Chairman) (or a pair of CEC members including at least one stroke physian for stoke events) and the pair will receive electronic notification that they have events ready for adjudication. The pairs will be rotated automatically in a manner that ensures that events are distributed to the members on an even basis. A full tracking system and audit including details of the date of dispatch to the CEC members will be utilised.

Those reported events ***not*** requiring adjudication by the CEC will be screened by the study team and CEC chair to ensure that potential endpoint events have not been missed. If the CEC chair is satisfied that a potential endpoint event requiring CEC adjudication has not been missed, the event will be signed-off electronically as "not a potential endpoint event". If the CEC chair considers that a potential endpoint event may have been missed, further information will be requested as required, by the study team and, if necessary, the event will be forwarded to the CEC for adjudication.

4.3 Phase 1 CEC review

Upon receipt of a batch of ***event packets*** containing the relevant event data for suspected events, the adjudicating pair of CEC physicians will review each one independently and will enter their adjudication decisions onto the web portal. This is the Phase 1 review. For each event where the two reviewers have agreed on a classification, the event is deemed classified.

If the classification decision of the two reviewers is not unanimous, the event will be referred to the CEC chair for decision. The CEC chair may either elect to classify the event or to bring the event to the CEC committee for discussion and a majority decision.

Insufficient information to classify an event

If, at any time, a CEC member decides that a classification verdict is not obtainable because of incomplete/insufficient data, the process outlined in section 4.4 will be followed.

4.4 Incomplete event data

If, having reviewed the event data pertaining to an event, the CEC chair, or a CEC member deems that the information therein is insufficient for the purposes of event adjudication, an electronic request for further information will be made directly to the study team, via the study web portal. This will be done by the CEC chair/ CEC member who will detail the specific information required. If the requested information is obtained and is deemed sufficient for the purpose of adjudication, the new *event package* (including the new, or updated, event data*)* will be distributed to the relevant pair of CEC members. This will then be reviewed independently by the pair of CEC members following the procedure outlined in section 4.3.

Alternatively, if the CEC chair or CEC member deems that the further information obtained is insufficient for the purpose of adjudicating an event, a new request for further information will be generated using the process described above.

In instances where it is confirmed that efforts to obtain requested information have been unsuccessful (e.g. because the study team has indicated that the information is not available despite best efforts to obtain it), classification of the event will proceed on the available information. Discussion at a CEC meeting or may be scheduled if the CEC chair feels this is necessary.

4.5 Phase 2 CEC review

The full CEC will convene at 3 monthly intervals throughout the study. These meetings

will be face to face if possible, else held virtually using either Teams or Zoom.

The primary objective of CEC meetings is the “Phase 2 review” and classification of those events for which a final classification decision has not been achieved by the Phase 1 review process outlined above (section 4.3). Phase 2 review of an event constitutes the discussion and adjudication of the event by the CEC as a group. Events discussed by the CEC as a group will be adjudicated by the majority vote. The Chair will have the casting vote if necessary. It is expected that at least 4 members including the Chair will be present at each CEC meeting.

The final classification decision for events discussed by the CEC as a group will be entered onto the web portal by the CEC chair. If the CEC are unable to arrive at a classification verdict for an event because of incomplete or inadequate information and it is felt that such information may be obtainable (e.g. the study team has *not* indicated that the information required is *not* available), the Chairman will detail the precise information/documentation that is needed to achieve classification and the study team will request this data using the process described in section 4.4. The event will be tabled and placed on the agenda for review at a subsequent CEC meeting when either the further information requested has been provided or when confirmation has been received that efforts to obtain the information have been unsuccessful. With respect to a death event; if, despite discussion, the cause of death remains unclear (and the study team has indicated that further information is *not* available despite best efforts to obtain it), the classification category “undetermined cause of death” will be used (see section 5.3.3).

4.6 Adjudication timelines

The CEC members will expect events to be allocated as they become available on the TIME web portal and will make every effort to enter their classification decisions onto the web-portal within 2 to 4 weeks from the time that the event data is received, although this may vary slightly. The prompt review/adjudication of events will be dependent on the CEC receiving the required event data (see 4.7) in a timely fashion and on the study team dealing with data-queries as promptly as possible.

For events requiring discussion at a CEC meeting, every effort will be made to ensure that such meetings take place in a timely fashion and that any final classification decisions reached are entered onto the web portal within 2 weeks of the meeting. If required, the above timelines may be amended as the study progresses, if the CEC and the other relevant parties agree.

4.7 Clinical data to be provided

Event data for each potential endpoint event will be posted on a website portal by the study team and will include:

a) A cover page that will identify the patient (by a unique patient identification number) and specify the event(s) to be adjudicated.
b) The relevant completed *event page(s)* [each with a unique identifying event number] with the narrative (clinical summary) section(s).

c) The appropriate supportive source documentation* for each event

*Where applicable and available, copies of the following source documentation should be provided for the following events:

Death event:

- Death certificate
- Autopsy report
- Relevant hospitalisation records (see "hospitalisation event", below)
- Discharge summary +/- clinical records or GP/paramedic records regarding the event

Hospitalisation event:

Relevant hospitalisation records, which may include:

- Hospital death/discharge summary
- Medical clerking records and relevant medical progress notes/continuation sheets (if required)
- Prescription charts (if required)

Potential myocardial infarction/biomarker positive ACS/angina/other chest pain event:

- ECGs: ECGs pertaining to the event and, if applicable, an ECG recorded before the event
- Cardiac enzyme/marker laboratory reports
- Other cardiovascular investigation reports as requested on the MI event page (e.g. exercise-ECG, echocardiography, myocardial perfusion scan)
- Cardiovascular operation/procedure reports (e.g coronary angiography)

Potential stroke/possible stroke event:

- Neuroimaging (CT brain, MRI brain, cerebral angiography) reports
- Key discharge or clinic letters
- Lumbar puncture report

Potential heart failure event

- ECGs: ECGs pertaining to the event and, if applicable, an ECG before the event
- Chest x-ray report
- Cardiac enzyme/marker laboratory report
- BNP/NT-proBNP report, if available
- Other relevant cardiovascular investigation reports (e.g. echocardiography, radionuclide ventriculography)
- Cardiovascular operation/procedure reports

5 Clinical Event definitions

5.1 Hospitalisation

Hospitalisation is defined as an emergency/unplanned admission to a hospital setting

(emergency room, observation or inpatient unit) that results in at least one overnight stay (i.e. a date change).

5.2 Non-fatal events

Date of onset

For purposes of classification, when classifying events that are a cause of hospitalisation, the date of admission will be used as the onset date. In cases where the stated date of admission differs from the date the patient first presented to hospital with the event (e.g. because of a period of observation in an emergency department, medical assessment unit or equivalent), the date of intial presentation to hospital will be used (provided that the patient had not been discharged from hospital in the interim). For events where an admission date is not applicable (e.g. events occuring *during* an ongoing hospitalisation), the date of onset as reported by the treating physician will be used.

5.2.1 Acute myocardial infarction

Note on biomarker elevations:

For cardiac biomarkers, laboratories should report an upper reference limit (URL). If the 99th percentile of the upper reference limit (URL) from the respective laboratory performing the assay is not available, then the URL for myocardial necrosis from the laboratory should be used. If the 99th percentile of the URL or the URL for myocardial necrosis is not available, the MI decision limit for the particular laboratory should be used as the URL.

Spontaneous acute myocardial infarction:

A rise and/or fall of cardiac biomarkers (troponin or CK-MB) should usually be detected (see note below) with at least one value above the upper reference limit (URL) together with evidence of myocardial ischemia with at least one of the following:

- Clinical presentation consistent with ischemia
- ECG evidence of acute myocardial ischaemia (as outlined in Table 1) or new left bundle branch block (LBBB)
- Development of pathological Q waves on the ECG (see Table 2)
- Imaging evidence of new loss of viable myocardium or new regional wall motion abnormality
- Autopsy evidence of acute myocardial infarction

If biomarkers are elevated from a prior infarction, then a spontaneous myocardial infarction is defined as:

a. One of the following:

- Clinical presentation consistent with ischemia
- ECG evidence of acute myocardial ischemia (as outlined in Table 1) or new left bundle branch block. [The events committee will adjudicate in the context of the sequential ECG changes that are commonly seen in acute ST elevation/acute non-ST elevation myocardial infarction.]
- New pathological Q waves (see Table 2). [The events committee will adjudicate in the context of the sequential ECG changes that are commonly seen in acute ST elevation/acute non-ST elevation myocardial infarction.]
- Imaging evidence of new loss of viable myocardium or new regional wall motion abnormality

- Autopsy evidence of acute myocardial infarction

AND

b. Both of the following:
- Evidence that cardiac biomarker values were decreasing (e.g. two samples 3-6 hours apart) prior to the suspected acute myocardial infarction*
- ≥ 20% increase (and > URL) in troponin or CK-MB between a measurement made at the time of the initial presentation with the suspected recurrent myocardial infarction and a further sample taken 3-6 hours later 1-3 hours where high sensitivity troponin assay is used.

*If biomarkers are increasing or peak is not reached, then a definite diagnosis of recurrent myocardial infarction is generally not possible.

Percutaneous coronary intervention-related acute myocardial infarction

Peri-percutaneous coronary intervention (PCI) acute myocardial infarction is defined by any of the following criteria. (Symptoms of cardiac ischemia are not required.)

1. Biomarker elevations within 48 hours of PCI:
   • Troponin or CK-MB (preferred) > 3 x URL ***and***
   • No evidence that cardiac biomarkers were elevated prior to the procedure;

   OR

   Both of the following must be true:
   - ≥ 50% increase in the cardiac biomarker result
   - Evidence that cardiac biomarker values were decreasing (e.g. two samples 3-6 hours apart) prior to the suspected acute myocardial infarction

2. New pathological Q waves or new left bundle branch block (LBBB).
   [If the PCI was undertaken in the context of an acute myocardial infarction, the events committee will adjudicate in the context of the sequential ECG changes that are commonly seen in acute ST elevation/acute non-ST elevation myocardial infarction.]

3. Autopsy evidence of acute myocardial infarction

Coronary artery bypass grafting-related acute myocardial infarction

Peri-coronary artery bypass graft surgery (CABG) acute myocardial infarction is defined by the following criteria. (Symptoms of cardiac ischemia are not required.)

1. Biomarker elevations within 72 hours of CABG:
   - Troponin or CK-MB (preferred) > 5 x URL **and**
   - No evidence that cardiac biomarkers were elevated prior to the procedure;

   OR

- Both of the following must be true:
  - ≥ 50% increase in the cardiac biomarker result
  - Evidence that cardiac biomarker values were decreasing (e.g. two samples 3-6 hours apart) prior to the suspected acute myocardial infarction

AND

2. One of the following:
   - New pathological Q-waves (preferably with evidence of persistence)
   - New LBBB (preferably with evidence of persistence)
   - Angiographically documented new graft or native coronary artery occlusion
   - Imaging evidence of new loss of viable myocardium

OR

3. Autopsy evidence of acute myocardial infarction

Note: For a diagnosis of acute myocardial infarction, elevation of cardiac biomarkers above the upper reference limit (or, if an URL is not available, above the local MI decision limit) should usually be present. If biomarkers are detectable but do not exceed the URL or the local MI decision limit, the classification "biomarker positive acute coronary syndrome" will be used, providing that the definition of this particular event-type (see below) is met.
However, myocardial infarction may be adjudicated for an event that has characteristics which are very suggestive of acute infarction but which does not meet the strict definition because biomarkers are not available (e.g. not measured) or are non-contributory (e.g. may have normalized).
Suggestive characteristics are:

- Typical cardiac ischemic-type pain/discomfort
  (except for suspected acute myocardial infarction occurring in the context of PCI or CABG where this requirement need not apply)

AND

- New ECG changes* or other evidence to support a diagnosis of acute myocardial infarction (e.g. imaging evidence of new loss of viable myocardium/new regional all motion abnormality or angiography demonstrating occlusive coronary thrombus)

*If ECG tracings are not available for review, the CEC may adjudicate on the basis of reported ECG changes that have been clearly documented in the case records or in the case report form.

Clinical classification of different types of myocardial infarction

Myocardial infarctions will be clinically classified as:

Type 1

Spontaneous myocardial infarction related to ischemia due to a primary coronary event such as plaque erosion and/or rupture, fissuring, or dissection.

Type 2
Myocardial infarction secondary to ischemia due to either increased oxygen demand or decreased supply, e.g. coronary artery spasm, coronary embolism, anaemia, arrhythmias, hypertension, or hypotension.

Type 3
Sudden unexpected cardiac death, including cardiac arrest, often with symptoms suggestive of myocardial ischemia, accompanied by presumably new ST elevation, or new LBBB, or evidence of fresh thrombus in a coronary artery by angiography and/or at autopsy, but death occurring before blood samples could be obtained, or at a time before the appearance of cardiac biomarkers in the blood.

Type 4a
Myocardial infarction associated with PCI.

Type 4b
Myocardial infarction associated with stent thrombosis as documented by angiography or at autopsy.

Type 5
Myocardial infarction associated with CABG

.
Myocardial infarctions will be further sub-classified as:
1. ST segment elevation myocardial infarction (STEMI).

or

2. Non-ST segment elevation myocardial infarction (NSTEMI).

or

3. Myocardial infarction, type (i.e. STEMI or NSTEMI) unknown.

Table 1: ECG manifestations of acute myocardial ischemia (in absence of left ventricular hypertrophy and left bundle branch block)
**ST elevation**<br>New ST elevation at the J-point in two anatomically contiguous leads with the cut-off<br>points: ≥ 0.2 mV in men (> 0.25 mV in men < 40 years) or ≥ 0.15 mV in women in leads V2-V3 and/or ≥ 0.1 mV in other leads.<br><br>**ST depression and T wave changes**<br>New horizontal or down-sloping ST depression ≥ 0.05 mV in two contiguous leads; and/or new T wave inversion ≥ 0.1 mV in two contiguous leads.<br><br>The above ECG criteria illustrate patterns consistent with myocardial ischemia. In patients with abnormal biomarkers, it is recognized that lesser ECG abnormalities may represent an ischemic response and may be accepted under the category of abnormal ECG findings.

Table 2: Pathological Q waves:
• Any Q-wave in leads V2-V3 ≥ 0.02 seconds or QS complex in leads V2 and V3<br>• Q-wave ≥ 0.03 seconds and ≥ 0.1 mV deep or QS complex in leads I, II, aVL, aVF, or V4-V6 in any two leads of a contiguous lead grouping (I, aVL, V6; V4-V6; II, III, and aVF) a<br><br>A The same criteria are used for supplemental leads V7-V9, and for the Cabrera frontal plane lead grouping.

5.2.2 Biomarker positive acute coronary syndrome (ACS)

Note: This does not include acute myocardial infarction which will be classified separately (see above).

Forthe diagnosis of biomarker positive ACS, the following criteria should be fulfilled:

There should be:

1 Clinical presentation consistent with ischaemia (e.g. typical cardiac ischaemic-type pain or discomfort).

and

2 Detectable cardiac biomarkers but without the fullfilment of the biomarker criteria outlined above for acute myocardial infarction.
[i.e. not exceeding the upper reference limit or, if an upper reference limit is not available, not exceeding the MI decision limit for the particular laboratory.]

and

3 The need for treatment with parenteral (intravenous, intra-arterial, buccal, transcutaneous or subcutaneous) anti-ischaemic/antithrombotic therapy and/or coronary revascularisation.

Note: The following are considered supportive of the diagnosis and, in general, at least one of these [(a), (b) or (c)] is expected to be present. However, this is not mandatory

if the criteria 1 to 3 (above) are met and provided that the adjudicator is satisfied that the totality of the information is consistent with the diagnosis.

(a) New and/or reversible ST segment or T wave changes on the ECG.
(b) Investigations undertaken in view of the event (e.g. exercise ECG or stress myocardial perfusion scan) showing evidence of reversible myocardial ischaemia,
(c) Coronary angiography showing angiographically significant coronary disease thought to be responsible for the patient's presentation. [If both invasive and CT angiographic imaging of the coronary arteries were performed, the results of the invasive coronary angiogram should take precedence.]

*5.2.3 Hospitalisation for troponin-negative cardiac chest pain**

For the diagnosis of hospitalisation for troponin-negative cardiac chest pain, there should be emergency/unplanned admission to a hospital setting (emergency room, observation or inpatient unit) that results in at least one overnight stay (i.e. a date change) with fulfillment of the following criteria:

There should be:

1 Clinical presentation consistent with ischaemia (e.g. typical cardiac ischaemic-type pain or discomfort) but without the fulfilment of the above diagnostic criteria for acute myocardial infarction or biomarker positive acute coronary syndrome.

and

2 The need for treatment with new or increased anti-anginal therapy (excluding sublingual nitrate therapy) and/or coronary revascularisation.

and

3 (a) New and/or reversible ST segment or T wave changes on the ECG.
Or
3 (b) Investigations undertaken in view of the event (e.g. exercise ECG or stress myocardial perfusion scan) showing evidence of reversible myocardial ischaemia.
Or
3 (c). Coronary angiography showing angiographically significant coronary disease thought to be responsible for the patient's presentation. [If both invasive and CT angiographic imaging of the coronary arteries were performed, the results of the invasive coronary angiogram should take precedence.]

and

4 The CEC should be satisified that angina was the primary reason for hospitalisation.

5.2.4 Hospitalisation for other chest pain (not endpoint)*

There should be:

- Emergency/unplanned admission to a hospital setting (emergency room, observation or inpatient unit) that results in at least one overnight stay i.e. a date change) due to chest pain but where the definitions (above) of acute myocardial infarction, biomarker positive ACS or angina are not met.
- The CEC or CEC chair should be satisfied that chest pain was the primary reason for
hospitalisation.

*These events are not study endpoints but the definitions provided for these events will be used by the CEC or CEC chair to categorise reported myocardial infarction, biomarker positive ACS, angina and chest pain events that do not meet the study definitions of acute myocardial infarction or biomarker positive acute coronary syndrome.

5.2.5 Stroke

Stroke is defined as an acute episode of neurological dysfunction caused by focal or global brain, spinal cord, or retinal vascular injury.

For the diagnosis of stroke, the following 4 criteria should usually be fulfilled:

1. **Rapid onset* of a focal/global neurological deficit with at least one of the following:**
   - Change in level of consciousness
   - Hemiplegia
   - Hemiparesis
   - Numbness or sensory loss affecting one side of the body
   - Dysphasia/aphasia
   - Hemianopia (loss of half of the field of vision of one or both eyes)
   - Complete/partial loss of vision of one eye
   - Other new neurological sign(s)/symptom(s) consistent with stroke

   *If the mode of onset is uncertain, a diagnosis of stroke may be made provided that there is no plausible non-stroke cause for the clinical presentation.

2. **Duration of a focal/global neurological deficit $\geq$ 24 hours**
   or
   < 24 hours if
   (i) this is because of at least one of the following therapeutic interventions:
   (a) pharmacologic i.e. thrombolytic drug administration.
   (b) non-pharmacologic i.e. neurointerventional procedure (e.g. intracranial angioplasty).
   or
   (ii) brain imaging available clearly documenting a new haemorrhage or infarct.
   or
   (iii) the neurological deficit results in death.

3. **No other readily identifiable non-stroke cause for the clinical presentation** (e.g. brain tumour, hypoglycaemia, peripheral lesion).

4. **Confirmation of the diagnosis by at least one of the following****:
   a) Neurology, stroke or neurosurgical specialist.
   b) brain imaging procedure (at least one of the following):
   (i) CT scan (a normal CT scan does not rule out cerebral infarction).

(ii) MRI scan.
(iii) cerebral vessel angiography.

c) lumbar puncture (i.e. spinal fluid analysis diagnostic of intracranial haemorrhage).

**If a stroke is reported but evidence of confirmation of the diagnosis by the methods outlined above is absent, the event will be discussed by the CEC members adjudicating the event with the CEC chair. In such cases, the event may be adjudicated as a stroke on the basis of the clinical presentation alone but only with agreement of the CEC chair.

Strokes will be further sub-classified as:

- Ischaemic (non-hemorrhagic) stroke
  (ie caused by an infarction of central nervous system tissue).

or

- Hemorrhagic stroke
  (ie caused by nontraumatic intraparenchymal, intraventricular or subarachnoid hemorrhage).

or

- Stroke type (i.e. hemorrhagic or ischaemic) unknown (i.e when imaging/other investigations are unavailable or inconclusive).

5.2.6 Hospitalisation for heart failure

For the diagnosis of hospitalisation for heart failure, there should be emergency/unplanned admission to a hospital setting (emergency room, observation or inpatient unit) that results in at least one overnight stay (i.e. a date change) with fulfillment of the following criteria:

There should be:

1. clinical manifestations of new or worsening heart failure including at least one of the following:
   - New or worsening dyspnoea on exertion
   - New or worsening dyspnoea at rest
   - New or worsening fatigue/decreased exercise tolerance
   - New or worsening orthopnoea
   - New or worsening PND (paroxysmal nocturnal dyspnoea)
   - New or worsening lower limb or sacral oedema
   - New or worsening pulmonary crackles/crepitations
   - New or worsening elevation of JVP (jugular venous pressure)
   - New or worsening third heart sound or gallop rhythm

And

2. Investigative evidence of structural or functional heart disease (if available) with at least *one* of the following:
   - Radiological evidence of pulmonary edema/congestion or cardiomegaly.
   - Imaging (e.g. echocardiography, cardiac magnetic resonance imaging, radionuclide ventriculography) evidence of an abnormality (e.g. left

ventricular systolic dysfunction, significant valvular heart disease, left ventricular hypertrophy).
- Elevation of BNP or NT-proBNP levels.
- Other investigative evidence of structural or functional heart disease (e.g evidence obtained from pulmonary artery catheterisation).

And

3. Need for new/increased therapy* *specifically* *for the treatment of heart failure* including at least one of the following:

   - New or increased oral therapy for the treatment of heart failure
   - (See note on oral therapy, below).
   - Initiation of intravenous diuretic, inotrope, vasodilator or other recognised intravenous heart failure treatment or uptitration of such intravenous therapy if already receiving it.
   - Mechanical or surgical intervention (e.g. mechanical or non-invasive ventilation, mechanical circulatory support, heart transplantation, ventricular pacing to improve cardiac function), or the use of ultrafiltration, hemofiltration, dialysis or other mechanical or surgical intervention that is specifically directed at treatment of heart failure.

   *If time does not allow for the initiation of, or an increase in, treatment directed at heart failure or if the circumstances were such that doing so would have been inappropriate (e.g. patient refusal), the CEC will adjudicate on clinical presentation and, if available, investigative evidence.

   Note on oral therapy: In general, for an event to qualify as *heart failure requiring hospitalisation* on the basis of *oral* heart failure therapy (i.e. in cases where none of the intravenous or non-pharmacological therapies listed above have been utilised), the new or increased oral therapy should include oral diuretics. However, in special cases, other new or increased oral therapy (e.g. hydralazine/long acting nitrate, aldosterone antagonist) may be accepted provided that the adjudication committee is satisfied that:

a) the new or increased oral therapy was primarily directed at treating clinical manifestations of new or worsening heart failure (rather than, for example, initiation or uptitration of heart failure therapy as part of the routine optimisation of medical therapy).

and

b) the totality of the evidence indicates that heart failure, rather than any other disease process, was the primary cause of the clinical presentation.

And

4. The CEC should be satisfied that heart failure was the primary disease process accounting for the clinical presentation.

5.3 Fatal events

In cases where a patient experiences an event and later dies due to that event, the

event causing death and the death will be considered as separate events *only* if they are separated by a change in calendar day. If the event causing death and the death occur on the same calendar day, death will be the only event classified.

5.3.1 Cardiovascular deaths

Cardiovascular death includes death resulting from an acute myocardial infarction, sudden cardiac death, death due to heart failure, death due to stroke and death due to other cardiovascular causes as follows:

Death due to Acute Myocardial Infarction refers to a death usually occurring up to 30 days after a documented acute myocardial infarction (verified either by the diagnostic criteria outlined above for acute myocardial infarction or by autopsy findings showing recent myocardial infarction or recent coronary thrombus) due to the myocardial infarction or its immediate consequences (e.g. progressive heart failure) and where there is no conclusive evidence of another cause of death.

If death occurs before biochemical confirmation of myocardial necrosis can be obtained, adjudication should be based on clinical presentation and other (e.g. ECG, angiographic, autopsy) evidence.
NOTE: This category will include sudden cardiac death, involving cardiac arrest, often with symptoms suggestive of myocardial ischemia, and accompanied by presumably new ST elevation*, or left new left bundle branch block*, or evidence of fresh thrombus in a coronary artery by coronary angiography and/or at autopsy, but death occurring before blood samples could be obtained, or at a time before the appearance of cardiac biomarkers in the blood (i.e. acute myocardial infarction Type 3 – see section 5.2, above).

*If ECG tracings are not available for review, the CEC may adjudicate on the basis of reported new ECG changes that have been clearly documented in the case records or in the case report form.

Death resulting from a procedure to treat an acute myocardial infarction [percutaneous coronary intervention (PCI), coronary artery bypass graft surgery (CABG)], or to treat a complication resulting from acute myocardial infarction, should also be considered death due to acute myocardial infarction
.
Death resulting from a procedure to treat myocardial ischemia (angina) or death due to an acute myocardial infarction that occurs as a direct consequence of a cardiovascular investigation/procedure/operation that was not undertaken to treat an acute myocardial infarction or its complications should be considered as a death due to other cardiovascular causes.

Sudden Cardiac Death refers to a death that occurs unexpectedly in a previously stable patient. The cause of death should not be due to another adjudicated cause (e.g. acute myocardial infarction Type 3 – see section 5.2 above).
The following deaths should be included.
a. Death witnessed and instantaneous without new or worsening symptoms

b. Death witnessed within 60 minutes of the onset of new or worsening symptoms unless a cause other than cardiac is obvious.
c. Death witnessed and attributed to an identified arrhythmia (e.g., captured on an ECG recording, witnessed on a monitor), or unwitnessed but found on implantable cardioverter-defibrillator review.
d. Death in patients resuscitated from cardiac arrest in the absence of pre-existing circulatory failure or other causes of death, including acute myocardial infarction, and who die (without identification of a non-cardiac aetiology) within 72 hours or without gaining consciousness; similar patients who died during an attempted resuscitation.
e. Unwitnessed death without any other cause of death identified (information regarding the patient's clinical status in the 24 hours preceding death should be provided, if available)

Sudden cardiac death events will be further subclassifed by the CEC as:
1) Sudden cardiac death due to a documented arrhythmia
(i.e. arrhythmia adjudged to be the primary terminal event ***and*** documented evidence of the arrhythmia)
2) "Other" sudden cardiac death (i.e. not classifiable as being due to a documented arrhythmia)
[e.g. insufficient evidence to suggest that an arrhythmia was the primary terminal event and/or no documented evidence of an arrhythmia]

Death due to Heart Failure refers to a death occurring in the context of clinically worsening symptoms and/or signs of heart failure without evidence of another cause of death (e.g. acute myocardial infarction).

Death due to heart failure should include sudden death occurring during an admission for worsening heart failure as well as death from progressive heart failure or cardiogenic shock following implantation of a mechanical assist device.

New or worsening signs and/or symptoms of heart failure include any of the following:
a. New or increasing symptoms and/or signs of heart failure requiring the initiation of, or an increase in, treatment directed at heart failure or occurring in a patient already receiving maximal therapy for heart failure
Note: If time does not allow for the initiation of, or an increase in, treatment directed at heart failure or if the circumstances were such that doing so would have been inappropriate (e.g. patient refusal), the CEC will adjudicate on clinical presentation and, if available, investigative evidence.
b. Heart failure symptoms or signs requiring continuous intravenous therapy or chronic oxygen administration for hypoxia due to pulmonary edema.
c. Confinement to bed predominantly due to heart failure symptoms.
d. Pulmonary edema sufficient to cause tachypnea and distress **not** occurring in the context of an acute myocardial infarction, worsening renal function (that is not wholly explained by worsening heart failure/cardiac function) or as the consequence of an arrhythmia occurring in the absence of worsening heart failure.
e. Cardiogenic shock **not** occurring in the context of an acute myocardial infarction or as the consequence of an arrhythmia occurring in the absence of worsening heart failure

Death due to Stroke refers to death after a documented stroke (verified by the diagnostic criteria outlined above for stroke or by typical post mortem findings) that is either a direct consequence of the stroke or a complication of the stroke and where there is no conclusive evidence of another cause of death.

NOTE: In cases of early death where confirmation of the diagnosis cannot be obtained, the CEC may adjudicate based on clinical presentation alone.
Death due to a stroke reported to occur as a direct consequence of a cardiovascular investigation/procedure/operation will be classified as death due to other cardiovascular cause.

Death due to Other Cardiovascular Causes refers to a cardiovascular death not included in the above categories [e.g. pulmonary embolism, cardiovascular intervention (other than one performed to treat an acute myocardial infarction or a complication of an acute myocardial infarction – see definition of death due to myocardial infarction, above), aortic aneurysm rupture, or peripheral arterial disease]. Mortal complications of cardiac surgery or non-surgical revascularisation should be classified as cardiovascular deaths.

5.3.2 Non-cardiovascular deaths

A non-cardiovascular death is defined as any death that is not thought to be due to a cardiovascular cause. There should be unequivocal and documented evidence of a non-cardiovascular cause of death.
Further subclassification of non-cardiovascular death will be as follows:

- Pulmonary
- Renal
- Gastrointestinal
- Infection (includes sepsis)
- Non-infectious (e.g., systemic inflammatory response syndrome (SIRS))
- Malignancy
- Hemorrhage, not intracranial
- Accidental/Trauma
- Suicide
- Non-cardiovascular surgery
- Other non-cardiovascular, specify

5.3.3 Undetermined cause of death

This refers to any death not attributable to one of the above categories of cardiovascular death or to a non-cardiovascular cause (e.g. due to lack of information such as a case where the only information available is "patient died"). It is expected that every effort will be made to provide the adjudicating committee with enough information to attribute deaths to either a cardiovascular or non-cardiovascular cause so that the use of this category is kept to a minimal number of patients.

6 *Approvals*

The following CEC and Sponsor representatives have approved this Charter.

Signature :	***Date***
Professor Chim C Lang (CEC Chair)<br>**Approved by email stored in TMF**	18-03-22
Dr Priya Nair<br><br>**Approved by email stored in TMF**	21-03-22
Dr Ify Mordi<br><br>**Approved by email stored in TMF**	10-03-22
Dr Jagdeep Singh	
Dr Zaid Iskandar (joined committee31/07/2018)	
Dr Ben New (Joined Committee August 2021)<br><br>**Approved by email stored in TMF**	14-03-22
Dr Ali Kashan (Joined Committee May 2021)	
Dr Qaiser Zeb (Joined Committee May 2021)	
Dr Mon Myat Oo (Joined Committee May 2021)	
Professor Tom MacDonald (CI TIME Study)	

Appendix A. ICD and OPCS CODES

Potential endpoints of interest to the CEC will be identified by searching electronically for the following codes:

CODE type	Code	Description
**DISEASE CODES**		
CHEST PAINS		
ICD10	R07.0	Pain in throat
ICD10	R07.1	Chest pain on breathing/painful respiration
ICD10	R07.2	Precordial pain
ICD10	R07.3	Other chest pain/anterior chest wall pain
ICD10	R07.4	Chest pain unspecified
CORONARY HEART DISEASE		
STABLE ANGINA		
ICD10	I20	Angina pectoris
ICD10	I25	Chronic ischaemic heart disease
ICD10	I250	Atherosclerotic cardiovascular disease, so described
ICD10	I251	Atherosclerotic heart disease
ICD10	I209	Angina pectoris, unspecified
ICD10	I201	Angina pectoris with documented spasm
ICD10	I258	Other forms of chronic ischaemic heart disease
ICD10	I259	Chronic ischaemic heart disease, unspecified
ICD10	I208	Other forms of angina pectoris
ACUTE CORONARY SYNDROMES		
ICD10	I200	Unstable angina
ICD10	I21	Acute myocardial infarction
ICD10	I210	Acute transmural myocardial infarction of anterior wall
ICD10	I211	Acute transmural myocardial infarction of inferior wall
ICD10	I212	Acute transmural myocardial infarction of other sites
ICD10	I213	Acute transmural myocardial infarction of unspecified site
ICD10	I214	Acute subendocardial myocardial infarction
ICD10	I219	Acute myocardial infarction, unspecified
ICD10	I22	Subsequent myocardial infarction
ICD10	I220	Subsequent myocardial infarction of anterior wall
ICD10	I221	Subsequent myocardial infarction of inferior wall
ICD10	I248	Other forms of acute ischaemic heart disease
ICD10	I249	Acute ischaemic heart disease, unspecified
ICD10	I24	Other acute ischaemic heart diseases
ICD10	I240	Coronary thrombosis not resulting in myocardial infarction
ICD10	I228	Subsequent myocardial infarction of other sites
ICD10	I229	Subsequent myocardial infarction of unspecified site
COMPLICATIONS OF ACUTE MYOCARDIAL INFARCTION		
ICD10	I23	Certain current complication follow acute myocardial infarct
ICD10	I230	Haemopericardium as curr comp folow acut myocard infarct

ICD10	I231	Atral sept defect as curr comp folow acut myocardal infarct

ICD10	I232	Ventric sep defect as curr comp fol acut myocardal infarc
ICD10	I233	Rup cardac wal withou haemopercard as cur comp fol ac MI
ICD10	I234	Rup chordae tendinae as curr comp fol acut myocard infarct
ICD10	I235	Rup papilary muscle as curr comp fol acute myocard infarct
ICD10	I236	Thromb atrium/auric append/vent as curr comp foll acute MI
ICD10	I238	Oth current comp following acute myocardial infarction
ICD10	I241	Dressler's syndrome
ICD10	I253	Aneurysm of heart
ICD10	I510	Cardiac septal defect, acquired
ICD10	I511	Rupture of chordae tendineae, not elsewhere classified
ICD10	I512	Rupture of papillary muscle, not elsewhere classified
ICD10	I513	Intracardiac thrombosis, not elsewhere classified
PREVIOUS MYOCARDIAL INFARCTION		
ICD10	I252	Old myocardial infarction
OTHER CORONARY HEART DISEASE		
ICD10	I254	Coronary artery aneurysm
ICD10	I256	Silent myocardial ischaemia
CEREBROVASCULAR DISEASES		
ICD10	I60	Subarachnoid haemorrhage
ICD10	I600	Subarachnoid haemorrhage from carotid siphon and bifurcation
ICD10	I601	Subarachnoid haemorrhage from middle cerebral artery
ICD10	I602	Subarachnoid haemorrhage from anterior communicating artery
ICD10	I603	Subarachnoid haemorrhage from posterior communicating artery
ICD10	I604	Subarachnoid haemorrhage from basilar artery
ICD10	I605	Subarachnoid haemorrhage from vertebral artery
ICD10	I606	Subarachnoid haemorrhage from other intracranial arteries
ICD10	I607	Subarachnoid haemorrhage from intracranial artery, unspec
ICD10	I608	Other subarachnoid haemorrhage
ICD10	I609	Subarachnoid haemorrhage, unspecified
ICD10	I61	Intracerebral haemorrhage
ICD10	I610	Intracerebral haemorrhage in hemisphere, subcortical
ICD10	I611	Intracerebral haemorrhage in hemisphere, cortical
ICD10	I612	Intracerebral haemorrhage in hemisphere, unspecified
ICD10	I613	Intracerebral haemorrhage in brain stem
ICD10	I614	Intracerebral haemorrhage in cerebellum
ICD10	I615	Intracerebral haemorrhage, intraventricular
ICD10	I616	Intracerebral haemorrhage, multiple localized
ICD10	I618	Other intracerebral haemorrhage
ICD10	I619	Intracerebral haemorrhage, unspecified
ICD10	I62	Other nontraumatic intracranial haemorrhage
ICD10	I620	Subdural haemorrhage (acute)(nontraumatic)
ICD10	I621	Nontraumatic extradural haemorrhage

ICD10	I629	Intracranial haemorrhage (nontraumatic), unspecified

ICD10	I63	Cerebral infarction
ICD10	I630	Cerebral infarct due to thrombosis of precerebral arteries
ICD10	I631	Cerebral infarction due to embolism of precerebral arteries
ICD10	I632	Cereb infarct due unsp occlusion or stenos precerebrl arts
ICD10	I633	Cerebral infarction due to thrombosis of cerebral arteries
ICD10	I634	Cerebral infarction due to embolism of cerebral arteries
ICD10	I635	Cerebrl infarct due unspec occlusion or stenos cerebrl arts
ICD10	I636	Cereb infarct due cerebral venous thrombosis, nonpyogenic
ICD10	I638	Other cerebral infarction
ICD10	I639	Cerebral infarction, unspecified
ICD10	I64X	Stroke, not specified as haemorrhage or infarction
ICD10	I65	Occlusion/stenos precerebral arts not result cerebrl infarct
ICD10	I650	Occlusion and stenosis of vertebral artery
ICD10	I651	Occlusion and stenosis of basilar artery
ICD10	I652	Occlusion and stenosis of carotid artery
ICD10	I653	Occlusion and stenosis of multip and bilat precerebrl arts
ICD10	I658	Occlusion and stenosis of other precerebral artery
ICD10	I659	Occlusion and stenosis of unspecified precerebral artery
ICD10	G45	Transient ischaemic attack/transient cerebral ischaemia
ICD10	I66	Occlusion/stenosis cerebral arts not result cerebral infarct
ICD10	I660	Occlusion and stenosis of middle cerebral artery
ICD10	I661	Occlusion and stenosis of anterior cerebral artery
ICD10	I662	Occlusion and stenosis of posterior cerebral artery
ICD10	I663	Occlusion and stenosis of cerebellar arteries
ICD10	I664	Occlusion and stenosis of multiple and bilat cerebrl arts
ICD10	I668	Occlusion and stenosis of other cerebral artery
ICD10	I669	Occlusion and stenosis of unspecified cerebral artery
ICD10	I67	Other cerebrovascular diseases
ICD10	I670	Dissection of cerebral arteries, nonruptured
ICD10	I671	Cerebral aneurysm, nonruptured
ICD10	I672	Cerebral atherosclerosis
ICD10	I673	Progressive vascular leukoencephalopathy
ICD10	I674	Hypertensive encephalopathy
ICD10	I675	Moyamoya disease
ICD10	I676	Nonpyogenic thrombosis of intracranial venous system
ICD10	I677	Cerebral arteritis, not elsewhere classified
ICD10	I678	Other specified cerebrovascular diseases
ICD10	I679	Cerebrovascular disease, unspecified
ICD10	I68	Cerebrovascular disorders in diseases classified elsewhere
ICD10	I680A	Cerebral amyloid angiopathy
ICD10	I681A	Cerebral arteritis in infect & parasit dis classif elsewh
ICD10	I682A	Cerebral arteritis in other diseases classified elsewhere
ICD10	I688A	Other cerebrovascular disorders in diseases EC
ICD10	I69	Sequelae of cerebrovascular disease
ICD10	I690	Sequelae of subarachnoid haemorrhage
ICD10	I691	Sequelae of intracerebral haemorrhage
ICD10	I692	Sequelae of other nontraumatic intracranial haemorrhage
ICD10	I693	Sequelae of cerebral infarction
ICD10	I694	Sequelae of stroke, not spec as haemorrhage or infarction

ICD10	I698	Sequelae of other and unspecified cerebrovascular diseases
ICD10	I70	Atherosclerosis
ICD10	G45	Transient cerebral ischaemic attacks and related syndromes
ICD10	G450	Vertebro-basilar artery syndrome
ICD10	G451	Carotid artery syndrome (hemispheric)
ICD10	G452	Multiple and bilateral precerebral artery syndromes
ICD10	G453	Amaurosis fugax
ICD10	G454	Transient global amnesia
ICD10	G458	Other transient cerebral ischaemic attacks and related synd
ICD10	G459	Transient cerebral ischaemic attack, unspecified
ICD10	G46	Vascular syndromes of brain in cerebrovascular diseases
ICD10	G460	Middle cerebral artery syndrome
ICD10	G461A	Anterior cerebral artery syndrome
ICD10	G462A	Posterior cerebral artery syndrome
ICD10	G463A	Brain stem stroke syndrome
ICD10	G464A	Cerebellar stroke syndrome
ICD10	G465A	Pure motor lacunar syndrome
ICD10	G466A	Pure sensory lacunar syndrome
ICD10	G467A	Other lacunar syndromes
ICD10	G468A	Oth vascular syndromes of brain in cerebrovascular dis
HEART FAILURE SYNDROMES		
ICD10	I50	Heart failure
ICD10	I500	Congestive heart failure
ICD10	I501	Left ventricular failure
ICD10	I509	Heart failure, unspecified
ICD10	J81	Pulmonary oedema
CARDIOMYOPATHIES		
ICD10	I42	Cardiomyopathy
ICD10	I420	Dilated cardiomyopathy
ICD10	I255	Ischaemic cardiomyopathy
ICD10	I421	Obstructive hypertrophic cardiomyopathy
ICD10	I422	Other hypertrophic cardiomyopathy
ICD10	I423	Endomyocardial (eosinophilic) disease
ICD10	I424	Endocardial fibroelastosis
ICD10	I425	Other restrictive cardiomyopathy
ICD10	I426	Alcoholic cardiomyopathy
ICD10	I427	Cardiomyopathy due to drugs and other external agents
ICD10	I428	Other cardiomyopathies
ICD10	I429	Cardiomyopathy, unspecified
ICD10	I43	Cardiomyopathy in diseases classified elsewhere
ICD10	I430A	Cardiomyopathy in infectious & parasitic diseases CE
ICD10	I431A	Cardiomyopathy in metabolic diseases
ICD10	I432A	Cardiomyopathy in nutritional diseases
ICD10	I438A	Cardiomyopathy in other diseases classified elsewhere
SHOCK (NOT ELSEWHERE CLASSFIED)		
ICD10	R57	Shock, not elsewhere classified

ICD10	R570	Cardiogenic shock
ICD10	R571	Hypovolaemic shock
ICD10	R578	Other shock
ICD10	R579	Shock, unspecified

HYPERTENSION AND RELATED CONDITIONS

ICD10	I11	Hypertensive heart disease
ICD10	I110	Hypertensive heart disease with (congestive) heart failure
ICD10	I119	Hypertensive heart disease without (conges) heart failure
ICD10	I12	Hypertensive renal disease
ICD10	I120	Hypertensive renal disease with renal failure
ICD10	I129	Hypertensive renal disease without renal failure
ICD10	I13	Hypertensive heart and renal disease
ICD10	I130	Hypertens heart and renal dis with (conges) heart failure
ICD10	I131	Hypertensive heart and renal disease with renal failure
ICD10	I132	Hyper heart and renal dis both (cong) heart and renal fail
ICD10	I139	Hypertensive heart and renal disease, unspecified
ICD10	I15	Secondary hypertension
ICD10	I150	Renovascular hypertension
ICD10	I151	Hypertension secondary to other renal disorders
ICD10	I152	Hypertension secondary to endocrine disorders
ICD10	I158	Other secondary hypertension
ICD10	I674	Hypertensive encephalopathy
ICD10	I159	Secondary hypertension, unspecified

HEART VALVE DISORDERS AND RELATED CONDITIONS

ICD10	I00X	Rheumatic fever without mention of heart involvement
ICD10	I01	Rheumatic fever with heart involvement
ICD10	I010	Acute rheumatic pericarditis
ICD10	I011	Acute rheumatic endocarditis
ICD10	I012	Acute rheumatic myocarditis
ICD10	I018	Other acute rheumatic heart disease
ICD10	I019	Acute rheumatic heart disease, unspecified
ICD10	I02	Rheumatic chorea
ICD10	I020	Rheumatic chorea with heart involvement
ICD10	I029	Rheumatic chorea without heart involvement
ICD10	I05	Rheumatic mitral valve diseases
ICD10	I050	Mitral stenosis
ICD10	I051	Rheumatic mitral insufficiency
ICD10	I052	Mitral stenosis with insufficiency
ICD10	I058	Other mitral valve diseases
ICD10	I059	Mitral valve disease, unspecified
ICD10	I34	Nonrheumatic mitral valve disorders
ICD10	I340	Mitral (valve) insufficiency
ICD10	I341	Mitral (valve) prolapse
ICD10	I342	Nonrheumatic mitral (valve) stenosis
ICD10	I348	Other nonrheumatic mitral valve disorders
ICD10	I349	Nonrheumatic mitral valve disorder, unspecified
ICD10	I35	Nonrheumatic aortic valve disorders
ICD10	I350	Aortic (valve) stenosis
ICD10	I351	Aortic (valve) insufficiency

ICD10	I352	Aortic (valve) stenosis with insufficiency
ICD10	I358	Other aortic valve disorders
ICD10	I359	Aortic valve disorder, unspecified
ICD10	I06	Rheumatic aortic valve diseases
ICD10	I060	Rheumatic aortic stenosis
ICD10	I061	Rheumatic aortic insufficiency
ICD10	I062	Rheumatic aortic stenosis with insufficiency
ICD10	I068	Other rheumatic aortic valve diseases
ICD10	I069	Rheumatic aortic valve disease, unspecified
ICD10	I07	Rheumatic tricuspid valve diseases
ICD10	I070	Tricuspid stenosis
ICD10	I071	Tricuspid insufficiency
ICD10	I072	Tricuspid stenosis with insufficiency
ICD10	I078	Other tricuspid valve diseases
ICD10	I079	Tricuspid valve disease, unspecified
ICD10	I36	Nonrheumatic tricuspid valve disorders
ICD10	I360	Nonrheumatic tricuspid (valve) stenosis
ICD10	I361	Nonrheumatic tricuspid (valve) insufficiency
ICD10	I362	Nonrheumatic tricuspid (valve) stenosis with insufficiency
ICD10	I368	Other nonrheumatic tricuspid valve disorders
ICD10	I369	Nonrheumatic tricuspid valve disorder, unspecified
ICD10	I08	Multiple valve diseases
ICD10	I080	Disorders of both mitral and aortic valves
ICD10	I081	Disorders of both mitral and tricuspid valves
ICD10	I082	Disorders of both aortic and tricuspid valves
ICD10	I083	Combined disorders of mitral, aortic and tricuspid valves
ICD10	I088	Other multiple valve diseases
ICD10	I089	Multiple valve disease, unspecified
ICD10	I09	Other rheumatic heart diseases
ICD10	I090	Rheumatic myocarditis
ICD10	I091	Rheumatic diseases of endocardium, valve unspecified
ICD10	I092	Chronic rheumatic pericarditis
ICD10	I098	Other specified rheumatic heart diseases
ICD10	I099	Rheumatic heart disease, unspecified
ICD10	I37	Pulmonary valve disorders
ICD10	I370	Pulmonary valve stenosis
ICD10	I371	Pulmonary valve insufficiency
ICD10	I372	Pulmonary valve stenosis with insufficiency
ICD10	I378	Other pulmonary valve disorders
ICD10	I379	Pulmonary valve disorder, unspecified
ICD10	I390A	Mitral valve disorders in diseases classified elsewhere
ICD10	I391A	Aortic valve disorders in diseases classified elsewhere
ICD10	I392A	Tricuspid valve disorders in diseases classified elsewhere
ICD10	I393A	Pulmonary valve disorders in diseases classified elsewhere
ICD10	I394A	Multiple valve disorders in diseases classified elsewhere
PULMONARY CIRCULATORY DISEASE		
ICD10	I26	Pulmonary embolism
ICD10	I260	Pulmonary embolism with mention of acute cor pulmonale
ICD10	I269	Pulmonary embolism without mention of acute cor pulmonale

ICD10	I27	Other pulmonary heart diseases
ICD10	I270	Primary pulmonary hypertension
ICD10	I271	Kyphoscoliotic heart disease
ICD10	I278	Other specified pulmonary heart diseases
ICD10	I279	Pulmonary heart disease, unspecified
ICD10	I28	Other diseases of pulmonary vessels
ICD10	I280	Arteriovenous fistula of pulmonary vessels
ICD10	I281	Aneurysm of pulmonary artery
ICD10	I288	Other specified diseases of pulmonary vessels
ICD10	I289	Disease of pulmonary vessels, unspecified
PERICARDIAL DISEASES		
ICD10	I30	Acute pericarditis
ICD10	I300	Acute nonspecific idiopathic pericarditis
ICD10	I301	Infective pericarditis
ICD10	I308	Other forms of acute pericarditis
ICD10	I309	Acute pericarditis, unspecified
ICD10	I31	Other diseases of pericardium
ICD10	I310	Chronic adhesive pericarditis
ICD10	I311	Chronic constrictive pericarditis
ICD10	I312	Haemopericardium, not elsewhere classified
ICD10	I313	Pericardial effusion (noninflammatory)
ICD10	I318	Other specified diseases of pericardium
ICD10	I319	Disease of pericardium, unspecified
ICD10	I32	Pericarditis in diseases classified elsewhere
ICD10	I320A	Pericarditis in bacterial diseases classified elsewhere
ICD10	I321A	Pericarditis in other infectious and parasitic diseases EC
ICD10	I328A	Pericarditis in other diseases classified elsewhere
ENDOCARDITIS AND CARDIAC INFECTIONS		
ICD10	I33	Acute and subacute endocarditis
ICD10	I330	Acute and subacute infective endocarditis
ICD10	I398A	Endocarditis, valve unspec, in diseases class elsewhere
ICD10	I339	Acute endocarditis, unspecified
ICD10	I38X	Endocarditis, valve unspecified
ICD10	I39	Endocarditis and heart valve disorders in diseases EC
ICD10	I520A	Other heart disorders in bacterial diseases EC
ICD10	I521A	Oth heart disorders in oth infectious and parasitic dis EC
ICD10	I980A	Cardiovascular syphilis
ICD10	I981A	Cardiovascular disorder other infectious and parasitic dis
MYOCARDITIS		
ICD10	I40	Acute myocarditis
ICD10	I400	Infective myocarditis
ICD10	I401	Isolated myocarditis
ICD10	I408	Other acute myocarditis
ICD10	I409	Acute myocarditis, unspecified
ICD10	I41	Myocarditis in diseases classified elsewhere
ICD10	I410A	Myocarditis in bacterial diseases classified elsewhere
ICD10	I411A	Myocarditis in viral diseases classified elsewhere
ICD10	I412A	Myocarditis in other infectious and parasitic diseases EC

ICD10	I514	Myocarditis, unspecified
ICD10	I418A	Myocarditis in other diseases classified elsewhere
CARDIAC CONDUCTING SYSTEM DISEASE		SE
ICD10	I441	Atrioventricular block, second degree
ICD10	I442	Atrioventricular block, complete
CARDIAC ARREST		
ICD10	I46	Cardiac arrest
ICD10	I460	Cardiac arrest with successful resuscitation
ICD10	I461	Sudden cardiac death, so described
ICD10	I469	Cardiac arrest, unspecified
CARDIAC ARRYTHMIAS		
ICD10	I47	Paroxysmal tachycardia
ICD10	I470	Re-entry ventricular arrhythmia
ICD10	I471	Supraventricular tachycardia
ICD10	I472	Ventricular tachycardia
ICD10	I479	Paroxysmal tachycardia, unspecified
ICD10	I48X	Atrial fibrillation and flutter
ICD10	I49	Other cardiac arrhythmias
ICD10	I490	Ventricular fibrillation and flutter
ICD10	I495	Sick sinus syndrome
ICD10	I498	Other specified cardiac arrhythmias
ICD10	I499	Cardiac arrhythmia, unspecified
DISEASES OF THE AORTA		
ICD10	I71	Aortic aneurysm and dissection
ICD10	I710	Dissection of aorta [any part]
ICD10	I711	Thoracic aortic aneurysm, ruptured
ICD10	I712	Thoracic aortic aneurysm, without mention of rupture
ICD10	I713	Abdominal aortic aneurysm, ruptured
ICD10	I714	Abdominal aortic aneurysm, without mention of rupture
ICD10	I715	Thoracoabdominal aortic aneurysm, ruptured
ICD10	I716	Thoracoabdominal aortic aneurysm, without mention of rupture
ICD10	I718	Aortic aneurysm of unspecified site, ruptured
ICD10	I719	Aortic aneurysm of unspec site, without mention of rupture
ICD10	I72	Other aneurysm
ICD10	I790A	Aneurysm of aorta in diseases classified elsewhere
ICD10	I791A	Aortitis in diseases classified elsewhere
PERIPHERAL ARTERIAL AND VENOUS DISEASES		
ICD10	I702	Atherosclerosis of arteries of extremities
ICD10	I708	Atherosclerosis of other arteries
ICD10	I720	Aneurysm of carotid artery
ICD10	I721	Aneurysm of artery of upper extremity
ICD10	I722	Aneurysm of renal artery
ICD10	I723	Aneurysm of iliac artery
ICD10	I724	Aneurysm of artery of lower extremity
ICD10	I728	Aneurysm of other specified arteries

ICD10	I729	Aneurysm of unspecified site
ICD10	I730	Raynaud's syndrome
ICD10	I731	Thromboangiitis obliterans [Buerger]
ICD10	I738	Other specified peripheral vascular diseases
ICD10	I739	Peripheral vascular disease, unspecified
ICD10	I74	Arterial embolism and thrombosis
ICD10	I740	Embolism and thrombosis of abdominal aorta
ICD10	I741	Embolism and thrombosis of other and unspec parts of aorta
ICD10	I742	Embolism and thrombosis of arteries of upper extremities
ICD10	I743	Embolism and thrombosis of arteries of lower extremities
ICD10	I744	Embolism and thrombosis of arteries of extremities, unspec
ICD10	I745	Embolism and thrombosis of iliac artery
ICD10	I748	Embolism and thrombosis of other arteries
ICD10	I749	Embolism and thrombosis of unspecified artery
ICD10	I77	Other disorders of arteries and arterioles
ICD10	I770	Arteriovenous fistula, acquired
ICD10	I771	Stricture of artery
ICD10	I772	Rupture of artery
ICD10	I776	Arteritis, unspecified
ICD10	I778	Other specified disorders of arteries and arterioles
ICD10	I80	Phlebitis and thrombophlebitis
ICD10	I800	Phlebitis/thrombophlebitis superfic vessels low extremties
ICD10	I801	Phlebitis and thrombophlebitis of femoral vein
ICD10	I802	Phlebitis/thrombophlebitis oth deep vessels low extremties
ICD10	I803	Phlebitis and thrombophlebitis of lower extremities, unspec
ICD10	I808	Phlebitis and thrombophlebitis of other sites
ICD10	I809	Phlebitis and thrombophlebitis of unspecified site
ICD10	I82	Other venous embolism and thrombosis
ICD10	I821	Thrombophlebitis migrans
ICD10	I822	Embolism and thrombosis of vena cava
ICD10	I823	Embolism and thrombosis of renal vein
ICD10	I828	Embolism and thrombosis of other specified veins
ICD10	I829	Embolism and thrombosis of unspecified vein
VARIOUS NON SPECIFIC CODES		
ICD10	I515	Myocardial degeneration
ICD10	I516	Cardiovascular disease, unspecified
ICD10	I517	Cardiomegaly
ICD10	I518	Other ill-defined heart diseases
ICD10	I519	Heart disease, unspecified
ICD10	I52	Other heart disorders in diseases classified elsewhere
ICD10	I51	Complications and ill-defined descriptions of heart disease
ICD10	I528A	Other heart disorders in other diseases classified elsewhere
ICD10	I97	Postprocedural disorders of circulatory system NEC
ICD10	I970	Postcardiotomy syndrome
ICD10	I971	Other functional disturbances following cardiac surgery
ICD10	I978	Other postprocedural disorders of circulatory system NEC
ICD10	I979	Postprocedural disorder of circulatory system, unspecified

ICD10	I98	Other disorders of circulatory system in diseases EC
ICD10	I988A	Other specified disorders of circulatory system in dis EC
ICD10	I99X	Other and unspecified disorders of circulatory system

PROCEDURE CODES
CARDIAC SURGICAL PROCEDURES

CORONARY ARTERY SURGERY		
OPCS4.4	K40.1	Saphenous vein graft replacement of one coronary artery
OPCS4.4	K40.2	Saphenous vein graft replacement of two coronary arteries
OPCS4.4	K40.3	Saphenous vein graft replacement of three coronary arteries
OPCS4.4	K40.4	Saphenous vein graft replacement of four or more coronary arteries
OPCS4.4	K40.8	Other specified saphenous vein graft replacement of coronary artery
OPCS4.4	K40.9	Unspecified saphenous vein graft replacement of coronary artery
OPCS4.4	K41.1	Autograft replacement of one coronary artery NEC
OPCS4.4	K41.2	Autograft replacement of two coronary arteries NEC
OPCS4.4	K41.3	Autograft replacement of three coronary arteries NEC
OPCS4.4	K41.4	Autograft replacement of four or more coronary arteries NEC
OPCS4.4	K41.8	Other specified other autograft replacement of coronary artery
OPCS4.4	K41.9	Unspecified other autograft replacement of coronary artery
OPCS4.4	K42.1	Allograft replacement of one coronary artery
OPCS4.4	K42.2	Allograft replacement of two coronary arteries
OPCS4.4	K42.3	Allograft replacement of three coronary arteries
OPCS4.4	K42.4	Allograft replacement of four or more coronary arteries
OPCS4.4	K42.8	Other specified allograft replacement of coronary artery
OPCS4.4	K42.9	Unspecified allograft replacement of coronary artery
OPCS4.4	K43.1	Prosthetic replacement of one coronary artery
OPCS4.4	K43.2	Prosthetic replacement of two coronary arteries
OPCS4.4	K43.3	Prosthetic replacement of three coronary arteries
OPCS4.4	K43.4	Prosthetic replacement of four or more coronary arteries
OPCS4.4	K43.8	Other specified prosthetic replacement of coronary artery
OPCS4.4	K43.9	Unspecified prosthetic replacement of coronary artery
OPCS4.4	K44.1	Replacement of coronary arteries using multiple methods
OPCS4.4	K44.2	Revision of replacement of coronary artery
OPCS4.4	K44.8	Other specified other replacement of coronary artery
OPCS4.4	K44.9	Unspecified other replacement of coronary artery
OPCS4.4	K45.1	Double anastomosis of mammary arteries to coronary arteries
OPCS4.4	K45.2	Double anastomosis of thoracic arteries to coronary arteries NEC
OPCS4.4	K45.3	Anastomosis of mammary artery to left anterior descending coronary artery
OPCS4.4	K45.4	Anastomosis of mammary artery to coronary artery NEC
OPCS4.4	K45.5	Anastomosis of thoracic artery to coronary artery NEC
OPCS4.4	K45.6	Revision of connection of thoracic artery to coronary artery

OPCS4.4	K45.8	Other specified connection of thoracic artery to coronary artery

OPCS4.4	K45.9	Unspecified connection of thoracic artery to coronary artery
OPCS4.4	K46.1	Double implantation of mammary arteries into heart
OPCS4.4	K46.2	Double implantation of thoracic arteries into heart NEC
OPCS4.4	K46.3	Implantation of mammary artery into heart NEC
OPCS4.4	K46.4	Implantation of thoracic artery into heart NEC
OPCS4.4	K46.5	Revision of implantation of thoracic artery into heart
OPCS4.4	K46.8	Other specified other bypass of coronary artery
OPCS4.4	K46.9	Unspecified other bypass of coronary artery
OPCS4.4	K47.1	Endarterectomy of coronary artery
OPCS4.4	K47.2	Repair of arteriovenous fistula of coronary a
OPCS4.4	K47.3	Repair of aneurysm of coronary artery
OPCS4.4	K47.4	Repair of rupture of coronary artery
OPCS4.4	K47.5	Repair of arteriovenous malformation of coronary artery
OPCS4.4	K47.8	Other specified repair of coronary artery
OPCS4.4	K47.9	Unspecified repair of coronary artery
OPCS4.4	K48.1	Transection of muscle-bridge of coronary artery
OPCS4.4	K48.2	Transposition of coronary artery NEC
OPCS4.4	K48.3	Open angioplasty of coronary artery
OPCS4.4	K48.4	Exploration of coronary artery
OPCS4.4	K48.8	Other specified other open operations on coronary artery
OPCS4.4	K48.9	Unspecified other open operations on coronary artery
TRANSPLANTATION PROCEDURES		
OPCS4.4	K01.1	Allotransplantation of heart and lung
OPCS4.4	K01.2	Revision of transplantation of heart and lung
OPCS4.4	K01.8	Other specified transplantation of heart and lung
OPCS4.4	K01.9	Unspecified transplantation of heart and lung
OPCS4.4	K02.1	Allotransplantation of heart NEC
OPCS4.4	K02.2	Xenotransplantation of heart
OPCS4.4	K02.3	Implantation of prosthetic heart
OPCS4.4	K02.4	Piggy back transplantation of heart
OPCS4.4	K02.5	Revision of implantation of prosthetic heart
OPCS4.4	K02.6	Revision of transplantation of heart NEC
OPCS4.4	K02.8	Other specified other transplantation of heart
OPCS4.4	K02.9	Unspecified other transplantation of heart
VALVE CARDIAC SURGERY		
OPCS4.4	K25.1	Allograft replacement of mitral valve
OPCS4.4	K25.2	Xenograft replacement of mitral valve
OPCS4.4	K25.3	Prosthetic replacement of mitral valve
OPCS4.4	K25.4	Replacement of mitral valve NEC
OPCS4.4	K25.5	Mitral valve repair NEC
OPCS4.4	K25.8	Other specified plastic repair of mitral valve
OPCS4.4	K25.9	Unspecified plastic repair of mitral valve
OPCS4.4	K26.1	Allograft replacement of aortic valve
OPCS4.4	K26.2	Xenograft replacement of aortic valve
OPCS4.4	K26.3	Prosthetic replacement of aortic valve
OPCS4.4	K26.4	Replacement of aortic valve NEC
OPCS4.4	K26.5	Aortic valve repair NEC
OPCS4.4	K26.8	Other specified plastic repair of aortic valve
OPCS4.4	K26.9	Unspecified plastic repair of aortic valve

OPCS4.4	K27.1	Allograft replacement of tricuspid valve
OPCS4.4	K27.2	Xenograft replacement of tricuspid valve
OPCS4.4	K27.3	Prosthetic replacement of tricuspid valve
OPCS4.4	K27.4	Replacement of tricuspid valve NEC
OPCS4.4	K27.5	Repositioning of tricuspid valve
OPCS4.4	K27.6	Tricuspid valve repair NEC
OPCS4.4	K27.8	Other specified plastic repair of tricuspid valve
OPCS4.4	K27.9	Unspecified plastic repair of tricuspid valve
OPCS4.4	K28.1	Allograft replacement of pulmonary valve
OPCS4.4	K28.2	Xenograft replacement of pulmonary valve
OPCS4.4	K28.3	Prosthetic replacement of pulmonary valve
OPCS4.4	K28.4	Replacement of pulmonary valve NEC
OPCS4.4	K28.5	Pulmonary valve repair NEC
OPCS4.4	K28.8	Other specified plastic repair of pulmonary valve
OPCS4.4	K28.9	Unspecified plastic repair of pulmonary valve
OPCS4.4	K29.1	Allograft replacement of valve of heart NEC
OPCS4.4	K29.2	Xenograft replacement of valve of heart NEC
OPCS4.4	K29.3	Prosthetic replacement of valve of heart NEC
OPCS4.4	K29.4	Replacement of valve of heart NEC
OPCS4.4	K29.5	Repair of valve of heart NEC
OPCS4.4	K29.6	Truncal valve repair
OPCS4.4	K29.7	Replacement of truncal valve
OPCS4.4	K29.8	Other specified plastic repair of unspecified valve of heart
OPCS4.4	K29.9	Unspecified plastic repair of unspecified valve of heart
OPCS4.4	K30.1	Revision of plastic repair of mitral valve
OPCS4.4	K30.2	Revision of plastic repair of aortic valve
OPCS4.4	K30.3	Revision of plastic repair of tricuspid valve
OPCS4.4	K30.4	Revision of plastic repair of pulmonary valve
OPCS4.4	K30.5	Revision of plastic repair of truncal valve
OPCS4.4	K30.8	Other specified revision of plastic repair of valve of heart
OPCS4.4	K30.9	Unspecified revision of plastic repair of valve of heart
OPCS4.4	K31.1	Open mitral valvotomy
OPCS4.4	K31.2	Open aortic valvotomy
OPCS4.4	K31.3	Open tricuspid valvotomy
OPCS4.4	K31.4	Open pulmonary valvotomy
OPCS4.4	K31.5	Open truncal valvotomy
OPCS4.4	K31.8	Other specified open incision of valve of heart
OPCS4.4	K31.9	Unspecified open incision of valve of heart
OPCS4.4	K32.1	Closed mitral valvotomy
OPCS4.4	K32.2	Closed aortic valvotomy
OPCS4.4	K32.3	Closed tricuspid valvotomy
OPCS4.4	K32.4	Closed pulmonary valvotomy
OPCS4.4	K32.8	Other specified closed incision of valve of heart
OPCS4.4	K32.9	Unspecified closed incision of valve of heart
OPCS4.4	K33.1	Aortic root replacement using pulmonary valve autograft with right ventricle to pulmonary artery valved conduit
OPCS4.4	K33.2	Aortic root replacement using pulmonary valve autograft with right ventricle to pulmonary artery valved conduit and aortoventriculoplasty
OPCS4.4	K33.3	Aortic root replacement using homograft
OPCS4.4	K33.4	Aortic root replacement using mechanical prosthesis
OPCS4.4	K33.5	Aortic root replacement

OPCS4.4	K33.6	Aortoventriculoplasty with pulmonary valve autograft
OPCS4.4	K33.8	Other specified operations on aortic root
OPCS4.4	K33.9	Unspecified operations on aortic root
OPCS4.4	K34.1	Annuloplasty of mitral valve
OPCS4.4	K34.2	Annuloplasty of tricuspid valve
OPCS4.4	K34.3	Annuloplasty of valve of heart NEC
OPCS4.4	K34.4	Excision of vegetations of valve of heart
OPCS4.4	K34.5	Closure of tricuspid valve
OPCS4.4	K34.6	Closure of pulmonary valve
OPCS4.4	K34.8	Other specified other open operations on valve of heart
OPCS4.4	K34.9	Unspecified other open operations on valve of heart
PERCUTANEOUS CLOSURE OF SEPTAL DEFECTS		
OPCS4.4	K13.1	Percutaneous transluminal repair of defect of interventricular septum using prosthesis
OPCS4.4	K13.2	Percutaneous transluminal repair of defect of interventricular septum NEC
OPCS4.4	K13.3	Percutaneous transluminal repair of defect of interatrial septum using prosthesis
OPCS4.4	K13.4	Percutaneous transluminal repair of defect of interatrial septum NEC
OPCS4.4	K13.5	Percutaneous transluminal repair of defect of unspecified septum using prosthesis
OPCS4.4	K13.8	Other specified transluminal repair of defect of septum
OPCS4.4	K13.9	Unspecified transluminal repair of defect of septum
OPCS4.4	K16.1	Percutaneous transluminal balloon atrial septostomy
OPCS4.4	K16.2	Percutaneous transluminal atrial septostomy NEC
OPCS4.4	K16.3	Percutaneous transluminal atrial septum fenestration closure with prosthesis
OPCS4.4	K16.4	Percutaneous transluminal atrial septum fenestration
OPCS4.4	K16.5	Percutaneous transluminal closure of patent oval foramen with prosthesis
OPCS4.4	K16.6	Percutaneous transluminal chemical mediated septal ablation
OPCS4.4	K16.8	Other specified other therapeutic transluminal operations on septum of heart
OPCS4.4	K16.9	Unspecified other therapeutic transluminal operations on septum of heart
CARDIAC SURGERY ON CONDUCTING SYSTEM OF HEART		
OPCS4.4	K52.1	Open ablation of atrioventricular node
OPCS4.4	K52.2	Epicardial excision of rhythmogenic focus
OPCS4.4	K52.3	Endocardial excision of rhythmogenic focus
OPCS4.4	K52.4	Open division of accessory pathway within heart
OPCS4.4	K52.5	Open division of conducting system of heart NEC
OPCS4.4	K52.6	Incision of tissue in atria
OPCS4.4	K52.8	Other specified open operations on conducting system of heart
OPCS4.4	K52.9	Unspecified open operations on conducting system of heart

INSERTION OF CIRCULATORY SUPPORT DEVICES		
OPCS4.4	K54.1	Open implantation of ventricular assist device

OPCS4.4	K54.2	Open removal of ventricular assist device
OPCS4.4	K54.8	Other specifed open heart assist operations
OPCS4.4	K54.9	Unspecified open heart assist operations
OPCS4.4	K56.1	Transluminal insertion of pulsation balloon into aorta
OPCS4.4	K56.2	Transluminal insertion of heart assist system NEC
OPCS4.4	K56.3	Transluminal maintenance of heart assist system
OPCS4.4	K56.4	Transluminal removal of heart assist system
OPCS4.4	K56.8	Other specified transluminal heart assist operations
OPCS4.4	K56.9	Unspecified transluminal heart assist operations
PERCUTANEOUS VALVE PROCEDURES		
OPCS4.4	K35.1	Percutaneous transluminal mitral valvotomy
OPCS4.4	K35.2	Percutaneous transluminal aortic valvotomy
OPCS4.4	K35.3	Percutaneous transluminal tricuspid valvotomy
OPCS4.4	K35.4	Percutaneous transluminal pulmonary valvotomy
OPCS4.4	K35.5	Percutaneous transluminal valvuloplasty
OPCS4.4	K35.6	Percutaneous transluminal pulmonary valve perforation and dilation
OPCS4.4	K35.7	Percutaneous transluminal pulmonary valve replacement
OPCS4.4	K35.8	Other specified therapeutic transluminal operations on valve of heart
OPCS4.4	K35.9	Unspecified therapeutic transluminal operations on valve of heart
ELECTROPHYSIOLOGY PROCEDURES		
OPCS4.4	K57.1	Percutaneous transluminal ablation of atrioventricular node
OPCS4.4	K57.2	Percutaneous transluminal ablation of conducting system of heart NEC
OPCS4.4	K57.3	Percutaneous transluminal removal of foreign body from heart
OPCS4.4	K57.4	Percutaneous transluminal ablation of accessory pathway
OPCS4.4	K57.5	Percutaneous transluminal ablation of atrial wall
OPCS4.4	K57.6	Percutaneous transluminal ablation of ventricular wall
OPCS4.4	K57.7	Percutaneous transluminal ablation for congenital heart malformation
OPCS4.4	K57.8	Other specified other therapeutic transluminal operations on heart
OPCS4.4	K57.9	Unspecified other therapeutic transluminal operations on heart
OPCS4.4	K58.1	Percutaneous transluminal mapping of conducting system of heart
OPCS4.4	K58.2	Percutaneous transluminal electrophysiological studies on conducting system of heart
OPCS4.4	K58.3	Percutaneous transluminal right ventricular biopsy
OPCS4.4	K58.4	Percutaneous transluminal left ventricular biopsy
OPCS4.4	K58.5	Transluminal intracardiac echocardiography
OPCS4.4	K58.8	Other specified diagnostic transluminal operations on heart
OPCS4.4	K58.9	Unspecified diagnostic transluminal operations on heart

PACEMAKERS AND DEFIBRILLATORS		
OPCS4.4	K59.1	Implantation of cardioverter defibrillator using one electrode lead

OPCS4.4	K59.2	Implantation of cardioverter defibrillator using two electrode leads
OPCS4.4	K59.3	Resiting of lead of cardioverter defibrillator
OPCS4.4	K59.4	Renewal of cardioverter defibrillator
OPCS4.4	K59.5	Removal of cardioverter defibrillator
OPCS4.4	K59.8	Other specified cardioverter defibrillator introduced through the vein
OPCS4.4	K59.9	Unspecified cardioverter defibrillator introduced through the vein
OPCS4.4	K60.1	Implantation of intravenous cardiac pacemaker system
OPCS4.4	K60.2	Resiting of lead of intravenous cardiac pacemaker system
OPCS4.4	K60.3	Renewal of intravenous cardiac pacemaker system
OPCS4.4	K60.4	Removal of intravenous cardiac pacemaker system
OPCS4.4	K60.5	Implantation of intravenous single chamber cardiac pacemaker system
OPCS4.4	K60.6	Implantation of intravenous dual chamber cardiac pacemaker system
OPCS4.4	K60.7	Implantation of intravenous biventricular cardiac pacemaker system
OPCS4.4	K60.8	Other specified cardiac pacemaker system introduced through vein
OPCS4.4	K60.9	Unspecified cardiac pacemaker system introduced through vein
OPCS4.4	K61.1	Implantation of cardiac pacemaker system NEC
OPCS4.4	K61.2	Resiting of lead of cardiac pacemaker system NEC
OPCS4.4	K61.3	Renewal of cardiac pacemaker system NEC
OPCS4.4	K61.4	Removal of cardiac pacemaker system NEC
OPCS4.4	K61.5	Implantation of single chamber cardiac pacemaker system
OPCS4.4	K61.6	Implantation of dual chamber cardiac pacemaker system
OPCS4.4	K61.7	Implantation of biventricular cardiac pacemaker system
OPCS4.4	K61.8	Other specified other cardiac pacemaker system
OPCS4.4	K61.9	Unspecified other cardiac pacemaker system

RIGHT AND LEFT HEART CATHETER PROCEDURES

OPCS4.4	K63.1	Angiocardiography of combination of right and left side of heart
OPCS4.4	K63.2	Angiocardiography of right side of heart NEC
OPCS4.4	K63.3	Angiocardiography of left side of heart NEC
OPCS4.4	K63.4	Coronary arteriography using two catheters
OPCS4.4	K63.5	Coronary arteriography using single catheter
OPCS4.4	K63.6	Coronary arteriography NEC
OPCS4.4	K63.8	Other specified contrast radiology of heart
OPCS4.4	K63.9	Unspecified contrast radiology of heart
OPCS4.4	K65.1	Catheterisation of combination of right and left side of heart NEC
OPCS4.4	K65.2	Catheterisation of right side of heart NEC
OPCS4.4	K65.3	Catheterisation of left side of heart NEC
OPCS4.4	K65.4	Catheterisation of left side of heart via atrial transeptal puncture
OPCS4.4	K65.8	Other specified catheterisation of heart
OPCS4.4	K65.9	Unspecified catheterisation of heart

PERICARDIAL PROCEDURES		
OPCS4.4	K67.1	Excision of lesion of pericardium

OPCS4.4	K67.8	Other specified excision of pericardium
OPCS4.4	K67.9	Unspecified excision of pericardium
OPCS4.4	K68.1	Decompression of cardiac tamponade
OPCS4.4	K68.2	Pericardiocentesis NEC
OPCS4.4	K68.8	Other specified drainage of pericardium
OPCS4.4	K68.9	Unspecified drainage of pericardium
OPCS4.4	K69.1	Freeing of adhesions of pericardium
OPCS4.4	K69.2	Fenestration of pericardium
OPCS4.4	K69.8	Other specified incision of pericardium
OPCS4.4	K69.9	Unspecified incision of pericardium
OPCS4.4	K71.1	Biopsy of lesion of pericardium
OPCS4.4	K71.2	Repair of pericardium
OPCS4.4	K71.3	Injection of therapeutic substance into pericardium
OPCS4.4	K71.4	Exploration of pericardium
OPCS4.4	K71.8	Other specified other operations on pericardium
OPCS4.4	K77.1	Percutaneous transluminal pericardiocentesis
OPCS4.4	K77.8	Other specified transluminal drainage of pericardium
OPCS4.4	K77.9	Unspecified transluminal drainage of pericardium
OPCS4.4	K71.9	Unspecified other operations on pericardium
CORONARY ANGIOPLASTY PROCEDURES		
OPCS4.4	K75.1	Percutaneous transluminal balloon angioplasty and insertion of 1-2 drug-eluting stents into coronary artery
OPCS4.4	K75.2	Percutaneous transluminal balloon angioplasty and insertion of 3 or more drug-eluting stents into coronary artery
OPCS4.4	K75.3	Percutaneous transluminal balloon angioplasty and insertion of 1-2 stents into coronary artery
OPCS4.4	K75.4	Percutaneous transluminal balloon angioplasty and insertion of 3 or more stents into coronary artery NEC
OPCS4.4	K75.8	Other specified percutaneous transluminal balloon angioplasty and stenting of coronary artery
OPCS4.4	K75.9	Unspecified percutaneous transluminal balloon angioplasty and stenting of coronary artery
OPCS4.4	K76.1	Percutaneous transluminal balloon dilation of cardiac conduit
OPCS4.4	K76.8	Other specified transluminal operations on cardiac conduit
OPCS4.4	K76.9	Unspecified transluminal operations on cardiac conduit
OPCS4.4	K78.1	Transluminal occlusion of left internal mammary artery side branch
OPCS4.4	K78.8	Other specified transluminal operations on internal mammary artery side branch
OPCS4.4	K78.9	Unspecified ?? transluminal operations on internal mammary artery side branch
OPCS4.4	K49.1	Percutaneous transluminal balloon angioplasty of one coronary artery
OPCS4.4	K49.2	Percutaneous transluminal balloon angioplasty of multiple coronary arteries
OPCS4.4	K49.3	Percutaneous transluminal balloon angioplasty of bypass graft of coronary artery
OPCS4.4	K49.4	Percutaneous transluminal cutting balloon angioplasty of coronary artery
OPCS4.4	K49.8	Other specified transluminal balloon angioplasty of coronary artery

OPCS4.4	K49.9	Unspecified transluminal balloon angioplasty of coronary artery
OPCS4.4	K50.1	Percutaneous transluminal laser coronary angioplasty
OPCS4.4	K50.2	Percutaneous transluminal coronary thrombolysis using streptokinase
OPCS4.4	K50.3	Percutaneous transluminal injection of therapeutic substance into coronary artery NEC
OPCS4.4	K50.4	Percutaneous transluminal atherectomy of coronary artery
OPCS4.4	K50.8	Other specified other therapeutic transluminal operations on coronary artery
OPCS4.4	K50.9	Unspecified other therapeutic transluminal operations on coronary artery
OPCS4.4	K51.1	Percutaneous transluminal angioscopy
OPCS4.4	K51.2	Intravascular ultrasound of coronary artery
OPCS4.4	K51.8	Other specified diagnostic transluminal operations on coronary artery
OPCS4.4	K51.9	Unspecified diagnostic transluminal operations on coronary artery

VARIOUS NON-SPECIFIC CARDIAC PROCEDURE CODES

OPCS4.4	K53.1	Inspection of valve of heart
OPCS4.4	K53.2	Exploration of heart NEC
OPCS4.4	K53.8	Other specified other incision of heart
OPCS4.4	K53.9	Unspecified other incision of heart
OPCS4.4	K55.1	Ligation of sinus of valsalva
OPCS4.4	K55.2	Open chest massage of heart
OPCS4.4	K55.3	Open removal of cardiac thrombus
OPCS4.4	K55.4	Open removal of cardiac vegetations NEC
OPCS4.4	K66.8	Other specified other operations of heart
OPCS4.4	K66.9	Unspecified other operations on heart

CEREBR0VASCULAR PROCEDURES

CAROTID ARTERY PROCEDURES

OPCS4.4	L29.1	Replacement of carotid artery using graft
OPCS4.4	L29.2	Intracranial bypass to carotid artery NEC
OPCS4.4	L29.3	Bypass to carotid artery NEC
OPCS4.4	L29.4	Endarterectomy of carotid artery and patch repair of carotid artery
OPCS4.4	L29.5	Endarterectomy of carotid artery NEC
OPCS4.4	L29.6	High-flow interposition extracranial to intracranial bypass from external carotid artery to middle cerebral artery
OPCS4.4	L29.7	Bypass of carotid artery by anastomosis of superficial temporal artery to middle cerebral artery
OPCS4.4	L29.8	Other specified reconstruction of carotid artery
OPCS4.4	L29.9	Unspecified reconstruction of carotid artery
OPCS4.4	L30.1	Repair of carotid artery NEC
OPCS4.4	L30.2	Ligation of carotid artery

OPCS4.4	L30.3	Open embolectomy of carotid artery
OPCS4.4	L30.4	Operations on aneurysm of carotid artery
OPCS4.4	L30.5	Operations on carotid body
OPCS4.4	L30.8	Other specified other open operations on carotid artery
OPCS4.4	L30.9	Unspecified other open operations on carotid artery

OPCS4.4	L31.1	Percutaneous transluminal angioplasty of carotid artery
OPCS4.4	L31.2	Arteriography of carotid artery
OPCS4.4	L31.3	Endovascular repair of carotid artery
OPCS4.4	L31.4	Percutaneous transluminal insertion of stent into carotid artery
OPCS4.4	L31.8	Other specified transluminal operations on carotid artery
OPCS4.4	L31.9	Unspecified transluminal operations on carotid artery
CEREBRAL ARTERY PROCEDURES		
OPCS4.4	L33.1	Excision of aneurysm of cerebral artery
OPCS4.4	L33.2	Clipping of aneurysm of cerebral artery
OPCS4.4	L33.3	Ligation of aneurysm of cerebral artery NEC
OPCS4.4	L33.4	Obliteration of aneurysm of cerebral artery NEC
OPCS4.4	L33.8	Other specified operations on aneurysm of cerebral artery
OPCS4.4	L33.9	Unspecified operations on aneurysm of cerebral artery
OPCS4.4	L34.1	Reconstruction of cerebral artery
OPCS4.4	L34.2	Anastomosis of cerebral artery
OPCS4.4	L34.3	Open embolectomy of cerebral artery
OPCS4.4	L34.4	Open embolisation of cerebral artery
OPCS4.4	L34.8	Other specified other open operations on cerebral artery
OPCS4.4	L34.9	Unspecified other open operations on cerebral artery
OPCS4.4	L35.1	Percutaneous transluminal embolisation of cerebral artery
OPCS4.4	L35.2	Arteriography of cerebral artery
OPCS4.4	L35.3	Percutaneous transluminal insertion of stent into cerebral artery
OPCS4.4	L35.8	Other specified transluminal operations on cerebral artery
OPCS4.4	L35.9	Unspecified transluminal operations on cerebral artery
SUBCLAVIAN/VERTEBRAL ARTERY PROCEDURES		
OPCS4.4	L37.1	Bypass of subclavian artery NEC
OPCS4.4	L37.2	Endarterectomy of vertebral artery
OPCS4.4	L37.3	Endarterectomy of subclavian artery and patch repair of subclavian artery
OPCS4.4	L37.4	Endarterectomy of subclavian artery NEC
OPCS4.4	L37.8	Other specified reconstruction of subclavian artery
OPCS4.4	L37.9	Unspecified reconstruction of subclavian artery
OPCS4.4	L38.1	Repair of subclavian artery NEC
OPCS4.4	L38.2	Ligation of subclavian artery
OPCS4.4	L38.3	Open embolectomy of subclavian artery
OPCS4.4	L38.4	Operations on aneurysm of subclavian artery
OPCS4.4	L38.8	Other specified other open operations on subclavian artery
OPCS4.4	L38.9	Unspecified other open operations on subclavian artery
OPCS4.4	L39.1	Percutaneous transluminal angioplasty of subclavian artery
OPCS4.4	L39.2	Percutaneous transluminal embolectomy of subclavian artery
OPCS4.4	L39.3	Percutaneous transluminal embolisation of subclavian artery
OPCS4.4	L39.4	Arteriography of subclavian artery
OPCS4.4	L39.5	Percutaneous transluminal insertion of stent into

OPCS4.4	L39.8	subclavian artery<br>Other specified transluminal operations on subclavian artery

OPCS4.4 subclavian artery	L39.9	Unspecified transluminal operations on
EVACUATION OF INTRACEREBRAL HAEMORRHAGE		
OPCS4.4 lobe of brain	A05.2	Evacuation of haematoma from temporal
OPCS4.4 cerebellum	A05.3	Evacuation of haematoma from

OPCS4.

Independent Data Monitoring Committee Charter

Treatment in Morning versus Evening Study (TIME)

Independent Data Monitoring Committee (IDMC) Charter

Independent Data Monitoring Committee Roster:

IDMC Members

- Professor Peter Sever (Professor of Clinical Pharmacology & Therapeutics, National Heart & Lung Institute, Imperial College London)
  p.sever@imperial.ac.uk

- Professor Kausik Ray, (Chair of Public Health, Imperial College London, (Charing Cross Campus), Room 320, 3rd Floor, Reynolds Building, St Dunstan's Road, London   W6 8RP)
  k.ray@imperial.ac.uk

- Professor Francesco P Cappuccio (Chair of Cardiovascular Medicine & Epidemiology, University of Warwick, Warwick Medical School, Gibbet Hill Road, Coventry   CV4 7AL)
  F.P.Cappuccio@warwick.ac.uk

- Professor Stuart Pocock (Professor or Medical Statistics, Medical Statistics Unit, London School of Hygiene and Tropical Medicine, Keppel Street, London WC1E 7HT)
  Stuart.Pocock@lshtm.ac.uk

IDMC Contacts

- ***Sponsor:*** The UNIVERSITY OF DUNDEE and NHS TAYSIDE ("Co-sponsors")

- ***Chief Investigator:***
  Professor Tom MacDonald (University of Dundee)
  t.m.macdonald@dundee.ac.uk Tel. 01382 383119

- ***IDMC Coordinator and TIME Project Manager:***
  Ms Wendy Saywood, (University of Dundee)
  w.saywood@dundee.ac.uk

- ***Trial Statistician (blinded):*** Prof Ian Ford (Robertson Centre for Biostatistics, Glasgow)
  Ian.Ford@glasgow.ac.uk

- ***Chair of the Trial Steering Committee (TSC):*** Prof Neil Poulter, (National Heart & Lung Institute, Imperial College London)
  n.poulter@imperial.ac.uk

APPROVING OFFICIALS

Name	Signature	Date
Prof Peter Sever<br>IDMC Chairperson	______________________	____________
Prof Kausik Ray	______________________	____________
Prof Francesco P Cappuccio	______________________	____________
Prof Stuart Pocock	______________________	____________
Professor Tom MacDonald<br>Chief Investigator<br>(Representative of the Sponsor - UNIVERSITY OF DUNDEE)	______________________	____________
Professor Ian Ford<br>Trial Statistician, Robertson Centre for Biostatistics, University of Glasgow	______________________	____________

Table of Contents

I	Scope of TIME STUDY IDMC and IDMC Charter	5
II	The role of the IDMC	5
III	Standard Constitution IDMC	5
IV	Independence	5
V	Composition of TIME STUDY IDMC	6
VI	IDMC Contacts and ad hoc Consultants	6
VII	TIME STUDY IDMC Responsibilities	6
VIII	UNIVERSITY OF DUNDEE Sponsor Responsibilities	8
IX	ROBERTSON CENTRE FOR BIOSTATISTICS Responsibilities	8
X	IDMC Coordinator Responsibilities	8
XI	TIME STUDY IDMC Member Training	8
XII	Ongoing Communications & Notifications to TIME STUDY IDMC	8
XIII	TIME STUDY IDMC Data Reports	8
XIV	TIME STUDY IDMC Meetings	9
XV	TIME STUDY IDMC Communication of Recommendation	11
XVI	Implementation of TIME STUDY IDMC Recommendations	11
XVII	TIME STUDY IDMC Document Handling & Records Retention	11
XVIII	Indemnity and Liability	11

I. Scope of TIME STUDY IDMC and IDMC Charter

The TIME STUDY IDMC (*Independent Data Monitoring Committee*) will independently monitor patient safety and efficacy information during this study.

The objective of the TIME STUDY IDMC Charter is to outline the specific purposes and functions of the IDMC and the procedures for data abstraction and data delivery conventions to and from the IDMC members for review purposes.

II. The role of the IDMC

The IDMC's main role is as follows:

- It is the only body involved in the trial that has access to the unblinded comparative data
- The role of its members is to monitor these data and make recommendations to the TSC (*Trial Steering Committee*) on whether there are any ethical or safety reasons why the trial should not continue
- The safety, rights and well-being of the trial participants are paramount
- The IDMC considers the need for any interim analysis advising the TSC regarding the release of data and/or information
- The IDMC may be asked by the TSC, Trial Sponsor or Trial Funder to consider data emerging from other related studies
- If funding is required above the level originally requested, the IDMC may be asked by the Chief Investigator, TSC, Trial Sponsor or Trial Funder to provide advice and, where appropriate, information on the data gathered to date in a way that will not compromise the trial
- Membership of the IDMC should be completely independent, small (3- 4 members) and comprise experts in the field, e.g. a clinician with experience in the relevant area or expert trial statistician
- Responsibility for calling and organising IDMC meetings lies with the Chief Investigator, in association with the Chair of the IDMC. The project team should provide the IDMC with a comprehensive report, the content of which should be agreed in advance by the Chair of the IDMC
- The IDMC should meet at least annually, or more often as appropriate, and meetings should be timed so that reports can be fed into the TSC
- Minutes of meetings (excluding closed sessions) should be sent to all IDMC members, the sponsor, the funder, the TSC and stored in the trial master file.

III. Standard Constitution IDMC

The following list identifies the minimum constitution requirements, a set of outline terms of reference and the primary reporting line for IDMC:

- Most primary research projects are required to establish a IDMC
- All IDMC members are to be independent
- Only appointed members will be entitled to vote and the chair will have a casting vote
- The minimum quorum for a meeting to conduct business is 67% of appointed members
- The chair and members to sign and maintain a log of potential conflicts and/or interests
- Attendance at IDMC meetings by non-members is at the discretion of the chair
- The primary IDMC reporting line is via the chair of the IDMC to the TSC

IV. Independence

The definition of independent is as follows:

- Not part of the same institution as any of the applicants or members of the project team
- Not part of the same institution that is acting as a recruitment or investigative centre
- Not related to any of the applicants or members of the project
- For the chair only- not an applicant on a rival proposal

V. Composition of TIME STUDY IDMC

The IDMC members are named on the IDMC roster. The IDMC will be composed of one Chairperson (Prof Peter Sever), two additional physicians and one biostatistician with clinical trial experience.

IDMC members will not be involved as investigators in the TIME study. In addition, IDMC members must not have a conflict of interest that would bias their review of trial data (e.g. IDMC members must not have a financial interest that could be substantially affected by the outcome of the study, strong views on the relative merits of the study intervention, or relationships with individuals in trial leadership positions that could be considered reasonably likely to affect their objectivity).

All IDMC members are expected to serve from study start until the study is completed (at least until end of study final database lock). Should it be necessary for a member to resign, the member must submit the effective date of resignation in writing to University of Dundee and the IDMC Chairperson. In the event a member resigns, University of Dundee, in consultation with the IDMC Chairperson, will initiate the process to identify a replacement member.

VI. IDMC Contacts and ad hoc Consultants

IDMC contacts and ad hoc consultants are not considered to be members of the IDMC. The official IDMC contacts are named on the IDMC roster.

The University of Dundee will assign IDMC Coordinators who will provide administrative, logistical, and coordinating services to the IDMC.

From the Sponsor, University of Dundee, an identified representative will serve as a primary contact person for the IDMC.

The Robertson Centre for Biostatistics, University of Glasgow will assign an unblinded biostatistician who will generate the IDMC Data Reports. In addition, this individual will be available to the IDMC, to provide consultation regarding the information presented within the IDMC Data Reports.

The IDMC may, with prior approval from University of Dundee, contact and involve selected expert consultants who may provide additional, relevant insight or expertise to the IDMC, regarding any specific issues that may arise.

As a rule, IDMC contacts and consultants must not attend closed sessions of IDMC Data Review Meetings. The IDMC Chairperson will ensure that IDMC contacts and consultants are not inappropriately exposed to fully unblinded and/or unblinded data made available to the IDMC.

VII. TIME STUDY IDMC Responsibilities

The TIME study IDMC is an independent expert advisory group commissioned and charged with the responsibility of evaluating cumulative safety, efficacy and other clinical trial data at regular intervals. As such, the primary objective of the IDMC is to monitor the safety of the subjects in the TIME study by reviewing the available clinical data at scheduled time points at least annually, or more often as appropriate, (which may be face to face or via teleconference) and on an *ad hoc* basis as needed.

After the review of each Data Report has been completed, the IDMC Chairperson will provide the official IDMC recommendation to the TSC regarding the appropriateness of continuing the study, from a safety and efficacy perspective, as well as any other recommendations relevant to study conduct and/or patient safety.

The operating procedures of the IDMC are based on and are in compliance with the US Food and Drug Administration's draft "Guidance for Clinical Trial Sponsors on the Establishment of Clinical Trial Data Monitoring Committees."

Specifically, the IDMC members are authorised and charged to perform the following functions:

- Provide approval for and operate in accordance with the specifications outlined in this IDMC Charter
- Monitor the safety of patients enrolled and to be enrolled in the TIME STUDY, and the efficacy of the trial, through scheduled review of accumulating clinical data from the ongoing clinical trial
- Review and evaluate the content of all Data Reports received
- Participate in and vote on IDMC recommendations.

Throughout the trial, the IDMC Chairperson will serve in a leadership role and will be authorised and charged with the following additional responsibilities:

- Conduct all IIDMC Data Review meetings
- Ensure that all relevant data have been reviewed by the IDMC members and that all issues have been addressed
- Ensure that blinded individuals (i.e. the IDMC Coordinator, IDMC contacts, and IDMC consultants) are not inappropriately exposed to confidential and/or unblinded data
- Ensure that only the members of the IDMC are present during IDMC deliberations, when IDMC recommendations are discussed and IDMC voting procedures are conducted
- Generate confidential, written minutes of all closed and executive sessions of any IDMC Meetings and maintain these minutes as confidential to IDMC members, only, until the final (end of study) database lock is complete
- Provide IDMC approval of records and minutes of open and final sessions of all IDMC meetings
- Maintain a secure central file of all data outputs received for IDMC review and all minutes of all sessions of IDMC meetings. Provide a copy of this file to the University of Dundee once the final (end of study) database lock is complete
- Communicate, author, sign, and provide the official, final recommendations of the IDMC within specified timelines and according to the specifications outlined in this charter. If the IDMC is divided in opinion on any major issue affecting the IDMC's recommendation to the University of Dundee, the IDMC Chairperson is responsible for assembling and presenting the majority and dissenting opinions for all recommendations considered
- Arrange for consultation(s) and/or request additional data, as deemed necessary.

VIII. UNIVERSITY OF DUNDEE Sponsor Responsibilities

The sponsor, University of Dundee, will have the following responsibilities with respect to the TIME STUDY IDMC:

- Ensure relevant clinical or other data available to University of Dundee on the safety of evening dosing are provided for communication to the IDMC
- Collaborate with the chief investigator to ensure that IDMC members are informed of trial progress and issues on a regular basis
- In preparation for data review meetings, collaborate with the chief investigator to prepare and provide a general summary of the status of the trial and any relevant clinical issues
- Provide Sponsor representation at all open and final sessions of IDMC meetings, as needed
- Arrange for fair and reasonable reimbursement to IDMC members for any study-related travel costs, such as transportation, lodging, and meals for the purposes of attending IDMC meetings.
- Provide a primary contact representative to receive recommendations from the IDMC
- Maintain ultimate responsibility for safe study conduct.

IX. ROBERTSON CENTRE FOR BIOSTATISTICS Responsibilities

Unblinded statisticians at the Robertson Centre for Biostatistics will programme and validate the reports that will be provided to the IDMC, run the reports and make the reports available to the IDMC in a secure manner.

The responsibilities of the Robertson Centre for Biostatistics are as follows:

- Operate in accordance with the specifications outlined in this IDMC Charter
- Work with IDMC members to determine the data that are necessary for the IDMC Data Reports
- Provide reports to the IDMC securely and in a timely fashion.
- Maintain an archive of electronic copies of datasets, programs and reports provided to the IDMC
- Provide consultation regarding the information presented in the IDMC Data Reports, as requested by the IDMC members.

X. IDMC Coordinator Responsibilities

The University of Dundee will provide an IDMC Coordinator(s) for the TIME STUDY. The IDMC Coordinator(s) will provide full administrative, logistical and coordinating support to the IDMC members.

The IDMC Coordinator will be charged with the following responsibilities:

- Secure approval for and operate in accordance with the specifications outlined in this IDMC Charter
- Serve as the primary, central point of contact for the IDMC members and as the main liaison between the TIME STUDY operations teams and the IDMC members
- Coordinate the implementation of the schedule for preparation and distribution of Data Reports to IDMC members
- Follow-up to verify that all data required by the IDMC are provided according to an agreed timeframe
- Obtain IDMC recommendation letters and distribute them, as described in this IDMC Charter
- Coordinate arrangements for all data review meetings and any IDMC *ad hoc* meetings, as outlined in this charter
- Maintain a central file of all key IDMC-related correspondences. Provide this file to University of Dundee after the final (end of study) database lock is completed
- University of Dundee will process IDMC member expense reports.

XI. TIME STUDY IDMC Member Training

All IDMC members will receive protocol overview training.

XII. Ongoing Communications & Notifications to TIME STUDY IDMC

The IDMC Coordinator will provide the IDMC Chairperson with copies of all Safety Letters related to the TIME STUDY.

XIII. TIME STUDY IDMC Data Reports

IDMC members will receive all IDMC Data Reports directly from the Robertson Centre for Biostatistics in a secure manner.

IDMC Data Reports will be provided to the IDMC members at least one week prior to scheduled data review meetings.

Data included in each IDMC Data Report will be cumulative-to-date at the time of the established data cut-off. The cut-off date for the data included in the Data Reports, as well as the current enrollment figures, will be stated in a cover letter.

The IDMC may request additional information on individual patients, as needed.

Data Reports for review by the IDMC will be presented in an unblinded fashion for both safety and efficacy reviews.

XIV. TIME STUDY IDMC Meetings

IDMC Data Review Meeting Frequency
Once all IDMC training is complete and the IDMC Charter has been finalised, the IDMC data review meetings will be scheduled at least annually, or more often as appropriate.

The committee will have the opportunity to make a recommendation of early stopping because of overwhelming evidence of benefit from study treatment based on interim analyses after approximately 50% and 75% of the target number of adjudicated study outcomes have been observed. Overwhelming evidence of benefit is defined as evidence of benefit of evening dosing over morning dosing ($P<0.001$).

The IDMC may formulate its own internal guidelines for monitoring other outcomes. These should be minuted.

In addition to these planned data review meetings, the IDMC will have the ability to hold *ad hoc* meetings, should they be deemed necessary.

IDMC Meeting Agendas
With input from the IDMC Chairperson and the University of Dundee, the IDMC Coordinator will establish the agenda for each planned data review meeting and for any ad hoc IDMC meetings.

IDMC Attendance
All four (4) IDMC members must be in attendance, in order for each IDMC data review meeting to be convened, and in order for voting procedures to be conducted.

IDMC Meeting Structure
It is anticipated that IDMC data review meetings will be either face to face or by teleconference.

IDMC data review meetings will normally consist of two sessions: an open session, and a closed session. If it is necessary for a representative of the Robertson Centre for Biostatistics to attend the closed session of an IDMC meeting, then the IDMC members should proceed to an executive session only including the IDMC members.

During open sessions the IDMC members, as well as the IDMC Coordinator, the Sponsor, a representative of the Robertson Centre for Biostatistics and any other IDMC contacts and consultants may be present. During open sessions, the IDMC members will receive a project update and will have an opportunity to discuss the progress of the trial with a representative of the Sponsor, the University of Dundee.

During closed sessions, the IDMC members will confidentially review the IDMC Data Report, deliberate and conduct voting procedures.

IDMC Voting Procedures: Recommendation to Sponsor
After review and discussion of each Data Report, the IDMC members will vote to determine the final IDMC recommendation within one of the following four options:

- Continue the study without modification
- Continue the study and amend the protocol, as specified
- Pause enrollment, pending resolution of a specified issue
- Terminate the study.

All IDMC members must participate in this voting procedure. The IDMC Chairperson will document the outcome of the vote. From review of the votes, the IDMC Chairperson will assess whether a consensus opinion has been achieved.

If IDMC consensus is not achieved, majority vote will determine the final decision of the IDMC, and the IDMC Chairperson will be responsible for assembling and presenting the majority and dissenting opinions to the University of Dundee, for all recommendations considered. If there is no majority vote the IDMC Chairperson will have the deciding vote.

If deemed necessary, the IDMC may elect to postpone the determination of an IDMC recommendation, pending external consultation(s) and/or receipt of additional data and a subsequent closed IDMC Data Review Meeting session.

The recommendations of the IDMC will be based on the members' clinical and biostatistical assessment of the cumulative safety data provided for review. As there will be interim analyses carried out, the IDMC members will be guided by formal statistical stopping guidelines. As part of the recommendation to the Sponsor, the IDMC may also make comments and suggestions that might enhance study performance, as deemed appropriate.

IDMC Meeting Minutes

IDMC Data Review meeting minutes will be divided by session and will reflect attendance, as well as whether each individual attended in person or via teleconference.

The IDMC Coordinator will produce minutes of open session of the IDMC meetings within a week of the meeting close out. The IDMC Coordinator will provide draft minutes to the IDMC Chairperson and the University of Dundee, for review and approval, before distribution. Once approved by the IDMC Chairperson and the University of Dundee, the IDMC Coordinator will distribute the final minutes as appropriate.

Since all details of IDMC deliberations must be kept strictly confidential among members of the IDMC, the closed and executive portions of the IDMC Data Review meeting minutes must remain confidential until after the study database is locked and the treatment groups for the entire study are unblinded. The IDMC Chairperson (or a designee selected among the IDMC members) will produce the minutes of the closed and executive sessions of IDMC meetings. These minutes will be distributed only to IDMC members, for the duration of the data collection until final database lock.

The member of the IDMC assigned to write the minutes should also prepare the recommendation letter from the Committee outlined in section XV.

The IDMC Chairperson will file all minutes from all sessions, centrally. After the study database is locked and the treatment groups for the entire study are unblinded, the IDMC Chairperson will forward the central file of all IDMC minutes for all sessions to the University of Dundee

XV. TIME STUDY IDMC Communication of Recommendation

Once the IDMC recommendation is finalised by IDMC vote, the IDMC Chairperson will communicate the IDMC recommendation to the Sponsor as follows:

- Formal, written communication of the IDMC recommendation will take the form of an IDMC recommendation letter from the IDMC Chairperson and should be provided to the IDMC Coordinator within one week after the IDMC recommendation is finalised
- Communications of IDMC recommendations will reflect the consensus opinion of the IDMC members. In the event that consensus cannot be reached, majority and dissenting opinions will be summarised and presented.

The IDMC Coordinator will receive the IDMC Recommendation Letter and distribute it to the TSC, Sponsor, Funder, Steering Committee and any other individuals as appropriate and as specified by the University of Dundee.

The IDMC Coordinator will also provide copies of the IDMC Recommendation Letters to investigators for submission to Institutional Review Boards (IRBs), upon receipt of investigator requests for this information.

XVI Implementation of TIME STUDY IDMC Recommendations

Any recommendations provided to the Sponsor by the IDMC will be treated as such. The University of Dundee after consultation with the study Steering Committee will hold the ultimate responsibility to implement the recommendations and take appropriate actions.

The Sponsor, University of Dundee, will notify the IDMC Chairperson in writing of actions taken in response to a given IDMC recommendation, for cases in which Sponsor action other than to continue the study without modification was recommended.

XVII. TIME STUDY IDMC Document Handling & Records Retention

Confidentiality

The IDMC will maintain a strictly confidential relationship to the TIME STUDY data. The IDMC will only reveal specific details and information associated with IDMC data review to appropriate parties, as specified by this IDMC Charter.

The materials provided to the IDMC should be considered and handled as strictly confidential in nature, as they have the potential to contain unblinded information regarding the trial, which cannot be communicated to non-IDMC members. As such, no member of the IDMC should release these data – or inappropriately disclose the contents – to unauthorised persons. If a situation occurs where confidential information is released to someone outside of the IDMC, the IDMC Co-ordinator should be informed and he or she will follow up immediately, in consultation with the chief investigator, to establish the best course of action to maintain study integrity.

With specific reference to the above instruction, IDMC members should take care to maintain the blind of the Sponsor and agents of the Sponsor (e.g., employees of the University of Dundee) at all times for the duration of the trial.

Data Handling

The IDMC Chairperson must retain a copy of all data reviewed by the IDMC in a central file.

Records Retention

The IDMC Chairperson should maintain a copy of the IDMC file (i.e., copies of all data reviewed by the IDMC and copies of final minutes of all sessions of any IDMC meeting) until two years after the end of the TIME study. After the two-year period, the IDMC Chairperson should contact the Sponsor, to determine if further retention and/or archiving are necessary.

XVIII. INDEMNIFICATION AND LIABILITY

Sponsor Indemnification

The University of Dundee (the Co-sponsor) shall indemnify, defend and hold harmless each IDMC member, from and against any and all losses, damages, liabilities, reasonable attorney fees, court costs, and expenses (collectively "Losses") resulting or arising from any third-party claims, actions, proceedings, investigations or litigation relating to or arising from or in connection with the performance of responsibilities by such IDMC member contemplated herein, except to the extent any such Losses have resulted from a breach of such IDMC member's obligations hereunder or from any willful or intentional misconduct of the IDMC member seeking indemnity hereunder. This indemnification provision shall not extend to any claim brought against a IDMC member by a Co-sponsor or their respective affiliates, its directors, officers, employees, agents and subcontractors.

IDMC Member Indemnification

Each IDMC member shall indemnify, defend and hold harmless each of the Co-sponsors, its affiliates, directors, officers, employees, agents and subcontractors (hereinafter collectively "Co-sponsors"), from and against any and all losses, damages, liabilities, reasonable attorney fees, court costs, and expenses (collectively "Losses") resulting or arising from any third-party claims, actions, proceedings, investigations or litigation relating to or arising from or in connection with the study TIME STUDY, provided that such Losses have resulted from any material breach of such IDMC member's obligations hereunder or from a judicial finding of willful or intentional misconduct of such IDMC member.

Indemnification Procedure

Each IDMC member seeking indemnification from the University of Dundee hereunder shall give the University of Dundee within seven (7) days written notice of any such claim or lawsuit (including a copy thereof) served upon it and shall fully cooperate with the University of Dundee and its legal representatives in the investigation of any matter the subject of indemnification. The IDMC member shall not unreasonably withhold its approval of the settlement of any claim, liability, or action covered by this indemnification provision.
A Co-sponsor seeking indemnification from any IDMC member hereunder shall give such IDMC member(s) prompt notice of any such claim or lawsuit (including a copy thereof) served upon it and shall fully cooperate with IDMC member and its legal representatives in the investigation of any matter the subject of indemnification. The Co-sponsors shall not unreasonably withhold its approval of the settlement of any claim, liability, or action covered by this indemnification provision.

Limitation of Liability
Notwithstanding anything contained herein, neither any IDMC member nor either Co-sponsor, nor any of its affiliates, directors, officers, employees, agents or subcontractors shall have any liability of any type (including, but not limited to, contract, negligence, and tort liability), for any loss of profits, opportunity or goodwill, or any type of special, incidental, indirect or consequential damage or loss in connection with or arising out of the obligations to be performed hereunder or otherwise in connection with the TIME study. In addition, the aggregate liability of the University of Dundee including for any material breach of its obligations hereunder, or otherwise in relation to the subject matter hereof (including that arising from negligence, delict, tort, or otherwise) during the TIME study or thereafter shall in no event exceed £2,000,000.

Other Remedies and Rights
The indemnification provided for herein shall be in addition, and not in limitation or in lieu of, any remedies or rights either party may have in law or equity or otherwise.

Statistical Analysis Plan

TIME

STATISTICAL ANALYSIS PLAN

Study Title:	Treatment in Morning versus Evening Study (TIME)		
Short Title:	TIME		
Funder:	British Heart Foundation		
Sponsor:	University of Dundee		
Protocol Version:	12.0	Date:	10/02/2021
SAP Version:	1.0	Date:	28/06/2022

		Signature	Date
Prepared by:	Dr Robin Young<br>Robertson Centre for Biostatistics		29/06/22
Approved by:	Prof Alex McConnachie<br>Assistant Director of Biostatistics, Robertson Centre for Biostatistics		29/06/2022
	Prof Tom MacDonald<br>Professor of Clinical Pharmacology and Pharmacoepidemiology, University of Dundee		29/06/2022

CONTENTS

1. Introduction 3
1.1. Study Background 3
1.2. Study Objectives 3
1.3. Study Design 3
1.4. Study Visits 3
1.5. Sample Size 4
1.6. Study Population 4
1.7. Statistical Analysis Plan (SAP) 4
1.7.1. SAP Objectives 4
1.7.2. General Principles 5
1.7.3. Study Protocol 5
1.7.4. Deviations to Those Specified in Protocol 5
1.7.5. Software 5
2. Analysis 5
2.1. Analysis Population 5
2.2. Baseline characteristics 6
2.3. Efficacy Outcomes 6
2.3.1. Primary Outcome 6
2.3.2. Primary Outcome – Subgroup Analyses 7
2.4. Secondary Outcomes 7
2.5. Exploratory Outcomes 7
2.6. Safety Outcomes 8
3. Tables and Figures 8
4. Listings 8
5. Document History 8

1. INTRODUCTION

1.1. STUDY BACKGROUND

Hypertension is a major risk factor for cardiovascular disease and affects people worldwide. Antihypertensive medications have been in routine use since the 1960s, and there are now many agents licensed for use in hypertension. It is widely accepted that lowering elevated blood pressure leads to a reduction in risks of heart attacks, strokes and all-cause mortality. Research using 24hr blood pressure monitoring has suggested that night-time blood pressure may be a better predictor of risk than daytime. It is suggested that medications administered at bedtime may have a greater protective effect than the same medications taken in the morning.

1.2. STUDY OBJECTIVES

The question to be definitively answered is whether, in usual care, nocturnal dosing of antihypertensive medication is better than morning dosing for reducing cardiovascular events.

Secondary questions examine whether there are any downsides to nocturnal dosing. Will patients accept nocturnal dosing? Nocturnal diuretic use causing urinary symptoms is likely to be an issue; data on this and how it can be managed is being collected (patients attempt nocturnal diuretic dosing, if not tolerated, they try 6 pm and finally revert to morning dosing). Nocturnal hypotension and its consequences (falls, fractures) are also being addressed. A sub-study also collects data on home blood pressure control.

Finally, four other sub-studies will be conducted. One will assess any effects on cognitive function and the others self-reported sleep quality, mood and chronotype (tendency towards being a “morning” or “evening” person). These will be analysed separately to the main study analysis.

1.3. STUDY DESIGN

TIME is a prospective, randomised, open-label, blinded end-point (PROBE design) controlled clinical trial.

1.4. STUDY VISITS

The study will follow the following schematic:

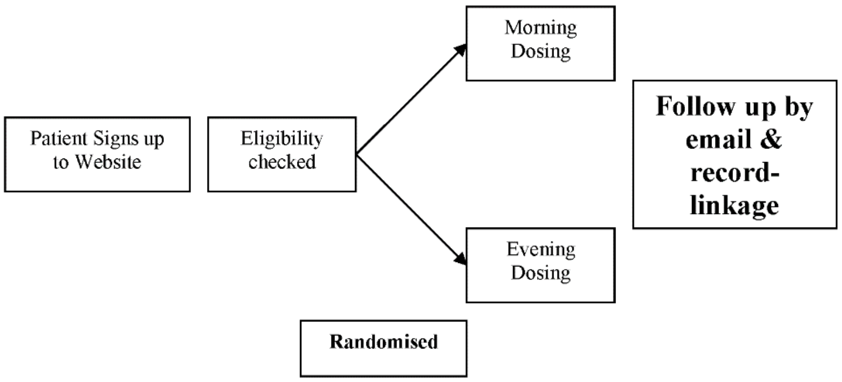

1.5. Sample Size

This sample size was adjusted during the study due to observed low cardiovascular event rates in several clinical trials involving similar populations. This adjustment was pre-specified in the published protocol[1].

For a 2-sided test to detect 20% superiority at 80% power, 631 events are needed. Based on the cardiovascular risk profile of participants recruited into the pilot phase of the study, a trial with a four year follow up period would need to randomise 19,740 participants. Because the primary analysis is intention to treat and because few participants are likely to withdraw consent for record-linkage follow up, only relatively minor (< 5%) inflation of the 19,740 participants was required to compensate. A target of 20,000 participants will be randomised. The original sample size estimate, based on a higher cardiovascular event rate was 10,269.

1.6. Study Population

The study population includes adults (≥18 years) in the UK who have hypertension and are prescribed at least one antihypertensive drug. For full details, see study protocol.

1.7. Statistical Analysis Plan (SAP)

1.7.1. SAP Objectives

The objective of this SAP is to describe the statistical analyses to be carried out for the final analysis of TIME. Any analyses that are undertaken and reported but not pre-specified in this SAP will be labelled as not pre-specified (post-hoc) in all reports.

1.7.2. General Principles

The primary analyses specified in this SAP will be carried out according to the intention-to-treat (ITT) principle, i.e. according to randomised group allocation, regardless of compliance with morning or evening dosing.

Following completion of the analyses specified in this SAP, alternative analyses will be considered to estimate the effect of evening vs. morning dosing amongst those individuals who are compliant with their assigned regimen.

Data will be summarised as a whole and by randomised group. The number of observations and the number of missing values will be reported. Continuous measures will be summarised using mean, standard deviation, median, quartiles, and range. Categorical measures will be reported using frequencies and percentages. Survival data will be summarised as the number of first events, crude percentage with events, and rates of events per 100 person-years of follow-up.

1.7.3. Study Protocol

The current study protocol at the time of writing is Version 12.0, dated 10th February 2021.

1.7.4. Deviations to Those Specified in Protocol

None

1.7.5. Software

Analyses will be carried out using R for Windows v3.4.1 or SAS for Windows v9.3, or higher versions of these programs.

2. Analysis

2.1. Analysis Population

All analyses will include all randomised patients and all available follow-up time to estimate the expected effect of dosing time.

Patients will be censored in the survival analysis at the earliest of the following dates:

- Date of withdrawal of all consent to participate further in the study including withdrawal from record-linkage follow up (note that some patients can withdraw from email follow up but allow record-linkage follow up and these will not be censored).
- Date of death (if it is not part of the endpoint under consideration)
- Censoring date for analysis, taken as 31/03/2021

2.2. Baseline characteristics

The following baseline characteristics will be tabulated:

- Demographics
    - Age
    - Sex

- Cardiovascular Risk Factors
    - Family History of cardiovascular disease
    - Systolic blood pressure
    - Diastolic blood pressure
    - Total cholesterol
    - BMI
    - Smoking status

- Medical History
    - Prior heart attack
    - Prior stroke
    - Impaired kidney function
    - Peripheral vascular disease
    - Any CVD (MI, CVA, PVD)
    - Diabetes
    - COPD
    - Chronic arthritis

- Medication use
    - Number of antihypertensive medications
    - Diuretic
    - ACE inhibitor
    - ARB
    - CCB
    - Beta blocker
    - Alpha-blocker
    - Other antihypertensive medication

2.3. Efficacy Outcomes

2.3.1. Primary Outcome

The primary outcome is the time to first event for the composite of vascular death, hospitalisation for non-fatal MI or non-fatal stroke.

A Cox Proportional Hazards model will be used to estimate the hazard ratio for evening vs morning dosing. The primary analysis will be unadjusted. The proportional hazards assumption will be assessed graphically using diagnostic plots. The estimated hazard ratio, 95% confidence interval and p-value will be reported. A cumulative incidence plot of events by treatment group will be produced.

2.3.2. Primary Outcome – Subgroup Analyses

Subgroup analyses for the primary outcome will be performed for the following baseline characteristics:

- Age (above/below median)
- Sex (male/female)
- BMI (above/below median)
- Smoking status (current/former/never)
- Prior heart attack (yes/no)
- Prior stroke (yes/no)
- Cardiovascular disease (MI, CVA, PVD) (yes/no)
- Diabetes (yes/no)
- Number of antihypertensive medications (≤3/>3)
- Use of ACE inhibitor (yes/no)
- Use of ARB (yes/no)
- Use of ACE inhibitor or ARB (yes/no)
- Use of CCB (yes/no)
- Use of beta blocker (yes/no)
- Use of alpha-blocker (yes/no)

For each subgroup analysis, the primary analysis model will be extended to include the subgroup variable, plus the interaction between the subgroup variable and randomised group. Within-subgroup intervention effect estimates will be reported with 95% confidence intervals and p-values, and a likelihood ratio test p-value will be reported as a test of the interaction.

2.4. Secondary Outcomes

Secondary outcomes are the times to first event classified as:

- CV death
- Hospitalisation for non-fatal stroke
- Hospitalisation for non-fatal MI
- All-cause mortality
- Hospitalisation or death from congestive heart failure.

Statistical methods used will be as per the primary outcome. No pre-specified sub-group analyses will be performed for the secondary outcomes.

2.5. Exploratory Outcomes

Exploratory outcomes will include:

- Stroke, by sub-type (ischaemic stroke, haemorrhagic stroke, subarachnoid haemorrhage, and other or non-specified)
- MI, by sub-type (total MI as well as the subgroups of STEMI and NSTEMI).
- Time to first reported non-adherence.

- Last known adherence at withdrawal, death, or end of trial (whichever is earliest).
- Home blood pressure readings submitted by a subset of participants will be averaged for each individual and treated as continuous variables (systolic blood pressure (SBP), diastolic blood pressure (DBP)). If times of readings are available, these data will also be graphically presented by time. We will test the following hypothesis:
    - Night-time dosing of antihypertensive medications will result in a lower Morning-Evening (ME) difference (morning BP - evening BP) when compared to morning-dosing.
- Antihypertensive medication expressed as the number of medications used will be compared between the randomised time of treatment groups at 3 monthly intervals.

2.6. Safety Outcomes

All pre-specified adverse events recorded by participants will be summarised by treatment group.

A table summarising pre-specified safety events (falls and fractures) by treatment group will be reported.

During the study, concern was expressed that night-time dosing of antihypertensive medications might increase the rate of diagnosis and progression of glaucomatous eye disease. We will therefore also report hospitalisations for glaucoma treatment by treatment group. Hospitalisations will be identified by either a) first diagnosis glaucoma, or b) glaucoma diagnosis in any position AND glaucoma-related surgical procedure.

3. Tables and Figures

The layout of the tables and figures will be agreed based on a report using dummy treatment codes prior to database lock. Approval of the format of this report will be documented separately.

4. Listings

Not applicable. As this is not a CTIMP there will not be a listing of all adverse events.

5. Document History

This is version 1.0 of the SAP for the TIME Study, dated 28th June 2022.

[1] Rorie DA, Rogers A, Mackenzie IS, Ford I, Webb DJ, Willams B, Brown M, Poulter N, Findlay E, Saywood W, MacDonald TM. Methods of a large prospective, randomised, open-label, blinded end-point study comparing morning versus evening dosing in hypertensive patients: the Treatment In Morning versus Evening (TIME) study. BMJ Open. 2016 Feb 9;6(2):e010313. doi: 10.1136/bmjopen-2015-010313. PMID: 26861939; PMCID: PMC4762112.
